# Use of Repeated Blood Pressure and Cholesterol Measurements to Improve Cardiovascular Disease Risk Prediction: An Individual-Participant-Data Meta-Analysis

**DOI:** 10.1093/aje/kwx149

**Published:** 2017-06-13

**Authors:** Ellie Paige, Jessica Barrett, Lisa Pennells, Michael Sweeting, Peter Willeit, Emanuele Di Angelantonio, Vilmundur Gudnason, Børge G. Nordestgaard, Bruce M Psaty, Uri Goldbourt, Lyle G Best, Gerd Assmann, Jukka T Salonen, Paul J Nietert, W. M. Monique Verschuren, Eric J Brunner, Richard A Kronmal, Veikko Salomaa, Stephan J L Bakker, Gilles R Dagenais, Shinichi Sato, Jan-Håkan Jansson, Johann Willeit, Altan Onat, Agustin Gómez de la Cámara, Ronan Roussel, Henry Völzke, Rachel Dankner, Robert W Tipping, Tom W Meade, Chiara Donfrancesco, Lewis H Kuller, Annette Peters, John Gallacher, Daan Kromhout, Hiroyasu Iso, Matthew Knuiman, Edoardo Casiglia, Maryam Kavousi, Luigi Palmieri, Johan Sundström, Barry R Davis, Inger Njølstad, David Couper, John Danesh, Simon G Thompson, Angela Wood

**Keywords:** cardiovascular disease, longitudinal measurements, repeated measurements, risk factors, risk prediction

## Abstract

The added value of incorporating information from repeated blood pressure and cholesterol measurements to predict cardiovascular disease (CVD) risk has not been rigorously assessed. We used data on 191,445 adults from the Emerging Risk Factors Collaboration (38 cohorts from 17 countries with data encompassing 1962–2014) with more than 1 million measurements of systolic blood pressure, total cholesterol, and high-density lipoprotein cholesterol. Over a median 12 years of follow-up, 21,170 CVD events occurred. Risk prediction models using cumulative mean values of repeated measurements and summary measures from longitudinal modeling of the repeated measurements were compared with models using measurements from a single time point. Risk discrimination (C-index) and net reclassification were calculated, and changes in C-indices were meta-analyzed across studies. Compared with the single-time-point model, the cumulative means and longitudinal models increased the C-index by 0.0040 (95% confidence interval (CI): 0.0023, 0.0057) and 0.0023 (95% CI: 0.0005, 0.0042), respectively. Reclassification was also improved in both models; compared with the single-time-point model, overall net reclassification improvements were 0.0369 (95% CI: 0.0303, 0.0436) for the cumulative-means model and 0.0177 (95% CI: 0.0110, 0.0243) for the longitudinal model. In conclusion, incorporating repeated measurements of blood pressure and cholesterol into CVD risk prediction models slightly improves risk prediction.

***Editor's note:****An invited commentary on this article appears on page 908*.

Established risk factors for cardiovascular disease (CVD), such as high systolic blood pressure (SBP) and elevated blood cholesterol levels, are routinely measured in general practice to predict CVD risk through the use of CVD risk scores (1, 2) and are also targets of treatments designed to modify risk (3). However, existing CVD risk scores are based on assessment of risk factors measured at only a single time point, and the added value of incorporating information from repeated measurements of risk predictors for prediction of CVD risk has not been thoroughly evaluated.

Repeated measurements of risk factors provide valuable information on an individual's risk trajectory over time and his or her within-person variability. The use of repeated measurements of risk predictors in the prediction of CVD risk has been mostly focused on correcting for regression dilution (4–6), using the change between 2 measurements (7), examining the use of updated measurements versus historical measurements (8, 9), and investigating the predictive ability of SBP variability (10, 11). However, there are modeling approaches available that allow modeling of longitudinal changes estimated from all available repeated measurements, including regression calibration approaches (12) that estimate “usual” risk factor levels. Another approach is longitudinal modeling that offers the advantage of being able to model intraindividual differences over time while still accounting for the correlation in repeated measurements (13). This approach was utilized in a population study to incorporate information on blood pressure trajectories into CVD risk prediction models (14) and in a recent simulation study that examined the potential of joint longitudinal and survival models for modeling systolic and diastolic blood pressure trajectories (15). We have previously compared alternative methods of including repeated measurements of SBP in a CVD risk prediction model using a single study, but found no benefit of joint models over simpler methods (16). However, to our knowledge, no previous studies have quantified the improvement in predictive ability that can be gained when repeated measurements of several risk predictors are included in CVD risk prediction algorithms as compared with the usual approach of using data from a single time point.

We conducted a large-scale individual-participant-data meta-analysis of 191,445 adults without a history of CVD at baseline to investigate the utilization of repeated measurements of continuous conventional risk factors, including SBP, total cholesterol, and high-density lipoprotein (HDL) cholesterol, in CVD risk prediction models. We used 2 methods of increasing complexity to model repeated measurements: 1) cumulative mean values and 2) individual-specific intercepts and slopes from mixed-effects linear regression models. Our aim was to quantify the change in risk discrimination and stratification of people according to their predicted 5-year CVD risk when the information from repeated measurements of risk predictors was added to the assessment of single measurements of risk factor levels used in standard risk scores.

## METHODS

### Study design

We used data from 38 prospective studies included in the Emerging Risk Factors Collaboration (17), encompassing the years 1962–2014. Studies were included in the current analysis if they met all of the following criteria: 1) at least 10 CVD deaths or vascular events (nonfatal myocardial infarction or stroke) or both were recorded during follow-up; 2) there were 5 or more years of follow-up; and 3) at least 1% of participants had repeated measurements of SBP, total cholesterol, and HDL cholesterol. Of the studies that met the inclusion criteria, participants were only included in the analysis if they did not have a recorded baseline history of CVD (i.e., myocardial infarction, angina, or stroke), were aged 40–79 years at baseline, and had complete information on baseline age, sex, smoking status, history of diabetes, SBP, and total and HDL cholesterol levels (i.e., the “conventional risk factors” included in standard clinical risk scores). Since the people who had repeated measurements were not a random sample of the original cohort, we aimed to minimize selection bias by including all participants in the analysis regardless of whether they had postbaseline repeated measurements of SBP, total cholesterol, and HDL cholesterol.

A list of study investigators and contributors is given in Web Appendix 1 (available at https://academic.oup.com/aje). Details of the included studies, including information on acronyms, study design, population source, and country, are provided in Web Appendix 2. The study was approved by Cambridgeshire Research Ethics Committee.

### Statistical analysis

The outcome in our analysis was a first CVD event, defined as nonfatal myocardial infarction or any stroke using well-defined study-specific criteria, or any fatal CVD. Participants were followed up until their first CVD event, their death, the end of the study, or loss to follow-up, whichever came first. We censored individuals at the end of follow-up or if they died from non-CVD causes. In registering fatal outcomes, the majority of contributing studies used the *International Classification of Diseases* (Eighth–Tenth Revisions), coding to at least 3 digits, and ascertainment was based on death certificates, with 31 of 38 studies also involving medical records, autopsy findings, and other supplementary sources.

Study-specific regression dilution ratios (18) were estimated for SBP, total cholesterol, and HDL cholesterol to summarize the within-person variability in the measurements over time.

Each study was randomly split into two 50% samples. Derivation of the longitudinal models included all available data from the first sample and data from the second sample censored at the prediction time (see schematic in Web Figure 1 (parts A and B)). Derivation of the Cox proportional hazards models included all available data from the first sample. Model validation was done using data from the second sample.

CVD risk prediction models were derived using Cox proportional hazards models, stratified by study and sex, and adjusting for baseline levels of conventional risk factors (age, smoking status, and history of diabetes) to estimate log hazard ratios. We compared 3 different models for incorporating available measurements of SBP, total cholesterol, and HDL cholesterol as predictors in the Cox models:

1. Model 1 used baseline measurements of SBP, total cholesterol, and HDL cholesterol.
2. Model 2 used cumulative mean values of SBP, total cholesterol, and HDL cholesterol, estimated from all previous repeated measurements and included in the model as time-varying covariates.
3. Model 3 used individual-specific random intercept and slope terms estimated from univariate mixed-effects linear regression models of repeated measurements of SBP, total cholesterol, and HDL cholesterol. Single mixed-effects models using data from all studies were fitted to the longitudinal data separately for SBP, total cholesterol, and HDL cholesterol, and results were adjusted for baseline levels of conventional risk factors, treating study as a random effect.

For the purposes of putting our results into context, we also generated a basic model including only age, sex, smoking status, and history of diabetes as predictors, and we examined the change in risk discrimination when single measurements of SBP, total cholesterol, and HDL cholesterol were each added separately to this model. The proportional hazards assumption was tested using previously described methods (19).

Among individuals contributing data to the model validation, 5-year CVD risk predictions were made at the time of the last repeat measurement taken prior to 5 years before the end of follow-up. For studies of long duration (>20 years), this was the time of the last repeat measurement taken prior to 15 years. No future data from individuals contributing data to the validation was used in model derivation or risk prediction. The time at which CVD risk prediction was estimated differed across individuals but was consistent across the 3 models being compared. In model 1, risk prediction was done using the last observation carried forward—that is, the latest measurement of blood pressure and cholesterol levels.

To quantify the change in risk prediction performance between the models using repeated measurements (models 2 and 3) and the model using single measurements (model 1), we compared discrimination (using the C-index, a measure of how well the model discriminates between individuals with and without CVD (20)) and risk reclassification using the validation data. The C-index and its changes were calculated within each study separately before pooling of results using meta-analysis weighted by the number of events occurring in individuals contributing to the validation in each study (21). Heterogeneity in C-indices between studies was quantified using the *I*^2^ statistic (22). Univariate meta-regression analyses were used to explore possible sources of heterogeneity in the changes in C-indices, including mean age, mean number of repeats, and maximum follow-up time in each study. We constructed reclassification tables using data from all studies combined to examine the movement of participants among predicted 5-year CVD risk categories (“low risk,” <2.5%; “medium risk,” 2.5%–3.74%; “high risk,” ≥3.75%). The 5-year risk cutpoints were chosen by halving the current US 10-year CVD treatment thresholds (23). Since treatment thresholds vary between countries and over time, we also calculated a continuous (“category-free”) net reclassification improvement (NRI) measure (24). Bootstrapping was used to obtain 95% confidence intervals for the overall NRI and its components.

Four sensitivity analyses were undertaken. First, we examined whether the study results varied when fitting separate survival models for each study and, for model 3, when study was treated as a fixed effect in the longitudinal models. For model 3, we also examined the effects of fitting mixed-effects longitudinal models separately for each study. Second, we examined whether the change in C-index was sensitive to the number of repeats available by restricting the data to participants with 2 or more postbaseline repeated measurements of SBP. Third, we investigated whether the change in C-index varied between younger (age <70 years) and older (age ≥70 years) participants. Finally, we restricted the analyses to studies with 10 years of follow-up and estimated 10-year CVD risk to examine the impact of modeling repeated measurements on longer-term risk prediction.

Analyses were performed using Stata statistical software, version 13.1 (StataCorp LP, College Station, Texas), and 95% confidence intervals were generated for all effect sizes.

## RESULTS

Among the 191,445 participants, the mean age at baseline was 55 years (standard deviation, 9.5; range, 40–79); 106,773 participants (56%) were men, and 62,519 (33%) were smokers. Table 1 shows the baseline characteristics of participants contributing data to the derivation and validation components, as well as the distribution of repeated measurements in the derivation data set. During a total of 2.5 million person-years of follow-up among all participants (median, 12.2 years; interquartile range, 7.0–17.7), there were 21,170 CVD events.

**Table 1.** Baseline Characteristics of Participants in the Derivation and Validation Data Sets and Distribution of Repeated Measurements in a Study of Cardiovascular Disease Risk Prediction, Emerging Risk Factors Collaboration, 1962–2014

Characteristic	Baseline						No. of Repeated Measurements in the Derivation Data							
	Derivation Data (*n* = 191,445)			Validation Data (*n* = 95,731)			0		1		2–4		≥5	
	No. of Persons	%	Mean (SD)	No. of Persons	%	Mean (SD)	No. of Persons	%	No. of Persons	%	No. of Persons	%	No. of Persons	%
Age, years			55.2 (9.5)			55.3 (9.5)								
Male sex	106,773	56		53,631	56									
Current smoker	62,519	33		31,488	33									
History of diabetes	16,311	9		8,218	9									
SBP, mm Hg			133.9 (20.9)			133.8 (20.9)	79,293	43	36,969	20	50,872	28	16,987	9
Total cholesterol, mmol/L			5.9 (1.2)			5.9 (1.2)	79,304	43	50,933	28	44,723	24	9,161	5
HDL cholesterol, mmol/L			1.3 (0.4)			1.3 (0.4)	90,816	49	49,566	27	37,022	20	6,717	4

Abbreviations: HDL, high-density lipoprotein; SBP, systolic blood pressure; SD, standard deviation.

Overall, there were 340,280 postbaseline measurements for SBP (an average of 1.8 per person), 266,361 for total cholesterol (an average of 1.4 per person), and 222,610 for HDL cholesterol (an average of 1.2 per person). Compared with those with no repeated measurements, participants with at least 1 postbaseline measurement were slightly older (mean age = 56.5 years vs. 54.7 years), more likely to be male (59% vs. 51%), less likely to be current smokers (29% vs. 38%), and more likely to have diabetes (10% vs. 6%); *P* values from χ^2^ tests were less than 0.001 for all comparisons. In the data used to derive the longitudinal models, 60% of participants had at least 1 postbaseline repeated measurement, and there were on average 2.3 years, 2.6 years, and 2.7 years between repeated measurements for SBP, total cholesterol, and HDL cholesterol, respectively. In the data used to estimate CVD risk prediction, there were on average 0.5 years, 0.5 years, and 0.8 years between the time of last observation and prediction time for SBP, total cholesterol, and HDL cholesterol, respectively.

Summary statistics, the distributions of repeated measurements, baseline characteristics, and scatterplots of repeated measurements and numbers of CVD events occurring over time among all participants are given in Web Tables 1–3 and Web Figure 2 for each included study. The regression dilution ratio for SBP was 0.52 (95% confidence interval (CI): 0.50, 0.55), indicating greater within-person variability than for total cholesterol (regression dilution ratio = 0.60, 95% CI: 0.58, 0.62) and HDL cholesterol (regression dilution ratio = 0.69, 95% CI: 0.67, 0.70) (Web Figure 3). Results from the longitudinal models of SBP, total cholesterol, and HDL cholesterol are shown in Web Table 4.

Across all risk prediction models, hazard ratios were statistically significant for all conventional risk factors (Table 2). The hazard ratios for the intercept of SBP and total cholesterol were slightly greater in models 2 and 3 than in model 1. The slopes of total cholesterol and HDL cholesterol, but not SBP, were associated with CVD risk in model 3.

**Table 2.** Hazard Ratios^a^ for Cardiovascular Disease in the Derivation Data for Each Model in a Study of Cardiovascular Disease Risk Prediction, Emerging Risk Factors Collaboration, 1962–2014

Risk Factor	Model 1^b^		Model 2^c^		Model 3^d^	
	HR	95% CI	HR	95% CI	HR	95% CI
Baseline age, years	1.08	1.08, 1.08	1.08	1.08, 1.08	1.09	1.09, 1.10
Current smoking (yes vs. no)	1.73	1.66, 1.80	1.73	1.66, 1.81	1.74	1.67, 1.81
History of diabetes (yes vs. no)	1.89	1.66, 1.80	1.90	1.78, 2.02	2.10	1.98, 2.24
SBP						
Intercept^e^	1.35	1.33, 1.38	1.40	1.37, 1.43	1.42	1.40, 1.45
Slope^f^					1.02	0.98, 1.07
Total cholesterol						
Intercept	1.19	1.16, 1.21	1.21	1.19, 1.24	1.26	1.23, 1.29
Slope					1.09	1.04, 1.15
HDL cholesterol						
Intercept	0.85	0.83, 0.87	0.84	0.82, 0.86	0.85	0.83, 0.88
Slope					0.94	0.88, 1.00

Abbreviations: CI, confidence interval; CVD, cardiovascular disease; HDL, high-density lipoprotein; HR, hazard ratio; SBP, systolic blood pressure; SD, standard deviation.

^a^ HRs from CVD risk models stratified by study and sex. Where appropriate, results were adjusted for baseline age, smoking status, history of diabetes, SBP, total cholesterol, and HDL cholesterol.

^b^ Model 1 used baseline measures of SBP, total cholesterol, and HDL cholesterol.

^c^ Model 2 used cumulative mean values of SBP, total cholesterol, and HDL cholesterol.

^d^ Model 3 used individual-level random intercepts and slopes for SBP, total cholesterol, and HDL cholesterol.

^e^ HRs for SBP, total cholesterol, and HDL cholesterol are given per SD increase. For model 3, these are the SD increases in the random intercept.

^f^ HRs for slopes are given per SD increase (using the SD of the random-effects slopes).

Of the 95,731 participants contributing data to the validation data set, 5,004 had a CVD event during the 5-year period after the time at which the CVD risk prediction was estimated. The distributions of absolute CVD risk predictions were similar across the 3 models (Web Figure 4).

### Changes in CVD risk discrimination

**Figure 1.**
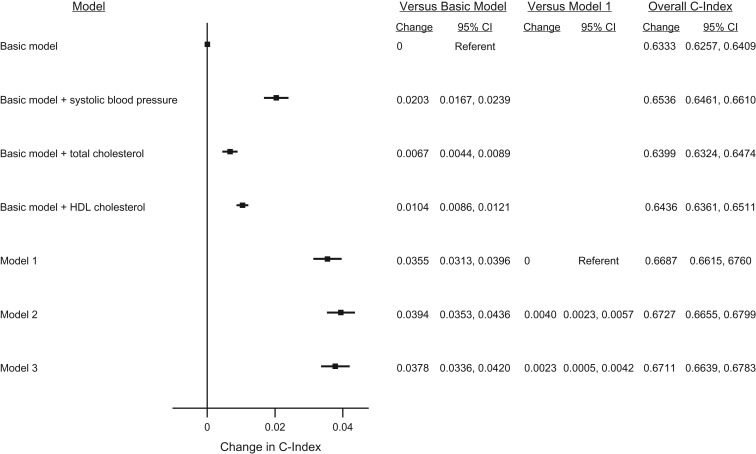
Change in cardiovascular disease (CVD) risk discrimination between the models in the validation data set in a study of CVD risk prediction, Emerging Risk Factors Collaboration, 1962–2014. A total of 66,353 people from 38 studies contributed to the estimation of 5-year CVD risk (i.e., contributed to the validation data and were alive at the time of CVD risk prediction). Of these, 2,667 people experienced a CVD event during the 5-year CVD risk estimation period. Models were stratified by sex and adjusted, where appropriate, for baseline conventional CVD risk factors: age, smoking status, history of diabetes, and baseline systolic blood pressure (SBP), total cholesterol, and high-density lipoprotein (HDL) cholesterol. Point estimates on the right-hand side of the graph relate to the improvement in risk prediction. Model 1 included variables from the basic model and baseline measures of SBP, total cholesterol, and HDL cholesterol. Model 2 included variables from the basic model and cumulative mean values of previous measures of SBP, total cholesterol, and HDL cholesterol. Model 3 included variables from the basic model and summary information from the longitudinal mixed-effects model of repeated measurements of SBP, total cholesterol, and HDL cholesterol.

The basic risk prediction model that included only age, sex, smoking status, and history of diabetes had an overall C-index of 0.6333 (95% CI: 0.6257, 0.6409). Adding single measurements of SBP, total cholesterol, and HDL cholesterol separately improved the C-index by 0.0203 (95% CI: 0.0167, 0.0239), 0.0067 (95% CI: 0.0044, 0.0089), and 0.0104 (95% CI: 0.0086, 0.0121), respectively (Figure 1). The addition of single measurements of these combined risk factors (model 1) to the basic risk prediction model improved risk discrimination by 0.0355 (95% CI: 0.0313, 0.0396). In comparison with model 1, using cumulative mean values and summary statistics from longitudinal models improved the overall C-index by 0.0040 (95% CI: 0.0023, 0.0057) and 0.0023 (95% CI: 0.0005, 0.0042), respectively. The change in C-index in model 3 was statistically significantly smaller than that in model 2 (*P* < 0.001). There was considerable between-study heterogeneity in C-indices (*I*^2^ = 99% for all models (*P* < 0.001); Web Figure 5) but less for the changes in C-indices (for model 2 vs. model 1, *I*^2^ = 67% (*P* < 0.001), and for model 3 vs. model 1, *I*^2^ = 74% (*P* < 0.001); Web Figure 6). There was no evidence that heterogeneity was explained by mean age, mean number of postbaseline repeated measurements, or the maximum follow-up time of the studies (Web Table 5).

### Changes in CVD risk classification

Incorporating repeated measurements of SBP, total cholesterol, and HDL cholesterol into the risk models improved their sensitivity (for model 2 vs. model 1, event NRI = 1.54% (95% CI: 0.84, 2.24), and for model 3 vs. model 1, event NRI = 2.14% (95% CI: 1.48, 2.79); Table 3). Only model 2 significantly improved specificity in comparison with model 1 (nonevent NRI = 2.15%, 95% CI: 1.97, 2.34; Table 3). The overall net reclassification of people according to 5-year CVD risk category was improved in both model 2 and model 3, with overall NRIs of 0.0369 (95% CI: 0.0303, 0.0436) and 0.0177 (95% CI: 0.0110, 0.0243), respectively. The overall NRI was improved in model 2 compared with model 3 (*P* < 0.001), primarily due to greater specificity in model 2 compared with model 3 (*P* < 0.001). Model 3 showed greater sensitivity than model 2 (*P* = 0.007).

**Table 3.** Change in Cardiovascular Disease Classification Using Repeated Measurements of Systolic Blood Pressure, Total Cholesterol, and High-Density Lipoprotein Cholesterol as Predictors in a Study of Cardiovascular Disease Risk Prediction, Emerging Risk Factors Collaboration, 1962–2014

NRI Measure and Model^a^	NRI					
	Event NRI	95% CI	Nonevent NRI	95% CI	Overall NRI^b^	95% CI
Categorical NRI						
Model 1	0	Referent	0	Referent	0	Referent
Model 2	0.0154	0.0084, 0.0224	0.0215	0.0197, 0.0234	0.0369	0.0303, 0.0436
Model 3	0.0214	0.0148, 0.0279	−0.0037	−0.0058, −0.0016	0.0177	0.0110, 0.0243
Continuous NRI						
Model 1	0	Referent	0	Referent	0	Referent
Model 2	0.0979	0.0686, 0.1272	0.3583	0.3521, 0.3645	0.4562	0.4256, 0.4867
Model 3	0.2234	0.2003, 0.2466	−0.1410	−0.1488, 0.1333	0.0824	0.0583, 0.1065

Abbreviations: CI, confidence interval; HDL, high-density lipoprotein; NRI, net reclassification improvement; SBP, systolic blood pressure.

^a^ Model 1: baseline measures of SBP, total cholesterol, and HDL cholesterol. Model 2: cumulative mean values of previous measurements of SBP, total cholesterol, and HDL cholesterol. Model 3: longitudinal mixed-effects model of repeated measurements of SBP, total cholesterol, and HDL cholesterol.

^b^ The overall NRI could range from −2 to +2.

Of the 5,004 people who contributed data to the validation data set and experienced a CVD event during the risk prediction period, 46 extra people (0.9%) were correctly identified as being at high risk under model 2 compared with model 1, while an extra 62 people (1.2%) were identified under model 3 compared with model 1 (Web Table 6). Of those who did not have an event during the risk prediction period (*n* = 59,122), an extra 745 people (1.3%) were reclassified as being at low risk under model 2 as compared with model 1. The results of the category-free NRI were consistent; compared with model 1, the overall NRI was 0.4562 (95% CI: 0.4256, 0.4867) for model 2 and 0.0824 (95% CI: 0.0583, 0.1065) for model 3 (Table 3).

### Sensitivity analyses

Changes in C-indices were similar when separate survival models were fitted for each study and stratified by sex (Web Figure 7). The overall change in C-index (0.0014, 95% CI: −0.0010, 0.0039) was slightly smaller than the change in C-index in the main results when separate longitudinal models were fitted for each study for model 3. When the analysis was restricted to participants with 2 or more postbaseline repeated measurements of SBP, the overall change in C-index (relative to model 1) increased for both model 2 (0.0072, 95% CI: 0.0030, 0.0115) and model 3 (0.0059, 95% CI: 0.0016, 0.0101), although the 95% confidence intervals were wide and overlapped with those observed in the main analysis. However, less heterogeneity was observed (for model 2 compared with model 1, *I*^2^ = 44% (*P* = 0.014), and for model 3 compared with model 1, *I*^2^ = 39% (*P* = 0.032); Web Figure 8). There was no evidence that the change in C-index varied between younger (age 40–69 years) and older (age ≥70 years) participants, although the available sample size was limited, since only 22 of the 38 studies could be included (Web Figure 9 (parts A and B)). In the final sensitivity analysis restricted to studies with 10 years of follow-up and estimating 10-year CVD risk, the changes in C-indices relative to model 1 were 0.0053 (95% CI: 0.0039, 0.0066) for model 2 and 0.0034 (95% CI: 0.0018, 0.0049) for model 3 (Web Figure 10).

## DISCUSSION

The current analysis of 829,251 postbaseline repeated measurements from 191,445 people across 38 cohorts reliably assessed the added benefit of incorporating repeated measurements of blood pressure and cholesterol into CVD risk prediction algorithms. Using cumulative mean values of repeated measurements of these risk predictors improved discrimination by a similar magnitude as when total cholesterol, a conventional risk predictor, was added to a basic risk prediction model including age, sex, smoking status, and history of diabetes. Stratification of patients according to risk is used to guide clinical treatment decisions, and interpretation of the clinical benefit of adding a predictor to a risk model typically takes into account both improvements in discrimination and risk reclassification, as well as the cost of obtaining the risk factor data.

In our study, both methods for modeling repeated measurements improved the sensitivity of the model, and the cumulative-means model produced slight gains in specificity compared with the use of single measurements of risk predictors. The results of our study suggest that an extra 0.9% and 1.2% of people would have been correctly identified as being at high risk under models 2 and 3, respectively, and could be identified as potential targets for treatment to reduce absolute risk levels if the models were applied in practice. Overall improvements in reclassification were greater in model 2 than in model 3, driven by gains in specificity. An extra 1.3% of people who did not experience CVD events were correctly identified as being at low risk under model 2, and thus adverse events related to unnecessary treatment in those individuals could potentially be avoided. Furthermore, improvement in risk prediction performance may be greater with the inclusion of more repeated measurements, as evidenced by the results of our sensitivity analysis. Although the 95% confidence intervals were wide and overlapped with those in the main analysis, there was some evidence of greater improvement in C-index, particularly for the longitudinal model (point estimates were 1.8 and 2.6 times those for models 2 and 3, respectively), when data were restricted to people with at least 2 postbaseline repeated measurements of SBP.

Two methods for modeling repeated data were compared: first, a simple cumulative-means approach, and second, a more complex longitudinal modeling approach used to generate a time-independent usual risk predictor level and a slope trajectory. Both methods offer the advantage of handling within-person variability in risk factors, while the second also allows for estimation of risk associations with slope trajectories. Thus, greater improvements in risk prediction ability will be more likely for risk factors with greater within-person variation and more variable trajectories over time. The hazard ratios for the intercepts of SBP and total cholesterol were slightly stronger under the longitudinal model than under the cumulative-means model, which likely reflects improvements in accounting for measurement error. There was little gained by adding slopes for these variables into our model. However, given the relatively short time of approximately 2 years on average between repeated measurements, incorporating slopes as well as intercepts from longitudinal modeling may result in greater improvements in risk prediction when there are more repeated measurements taken over a longer time period.

Our results support earlier findings by Paynter et al. (12), who used regression calibration to estimate long-term “usual” levels of SBP, total cholesterol, and HDL cholesterol as risk predictors for coronary heart disease. Although their approach showed no statistically significant improvement in C-indices compared with the use of single measurements of risk factors—possibly due to the smaller sample size of 12,834 participants—the changes in C-indices (0.003 for men and 0.005 for women) were similar to those seen in the present study.

This was the first study, to our knowledge, to quantify the improvement in CVD risk prediction gained from using repeated measurements of multiple risk predictors. The high number of CVD events and the large sample size meant that we could more precisely quantify improvements in predictive ability, and the inclusion of participants from 17 countries enhances the reliability and generalizability of the results. In addition to these major strengths, our study had some limitations. Firstly, in order to maximize the availability of repeated measurements for the model derivation and the length of follow-up in the validation data set, we estimated 5-year CVD risk rather than the more commonly used 10-year CVD risk, although we assessed 10-year CVD risk in the subset of studies with 10 or more years of follow-up. Secondly, we restricted our investigation to incorporating repeated measurements of continuous conventional risk factors, but in future research, investigators could examine the use of repeated measurements of other biochemical factors or conventional categorical risk factors, such as smoking status. Thirdly, we were unable to adjust the results for changes in medication use over time, as this was recorded in too few of the included studies. However, we included only participants without CVD at baseline. Hypertension was not well managed at the time many of the studies were conducted, and the trajectories of total and HDL cholesterol remained mostly stable over time, suggesting that medication use was unlikely to have affected the main results. Fourthly, there was considerable heterogeneity in the C-indices. This is probably explained in part by the inclusion of participants without postbaseline measurements in the main analysis; less heterogeneity was observed in the sensitivity analysis restricted to participants with 2 or more postbaseline measurements of SBP. Finally, the inclusion of participants without postbaseline repeated measurements in the main analysis also probably diluted the results. This was evidenced in the sensitivity analysis restricted to participants with 2 or more postbaseline measurements of SBP, which showed some evidence of greater improvements in discrimination than were observed in the main analysis.

Our aim in this study was to compare 2 different methods of utilizing already-available data from repeated measurements of continuous conventional risk predictors, but this study does not provide evidence on whether such extra measurements of risk factors should be taken in practice or how frequently the factors should be measured. Instead, the results of this study suggest that, in principle, the inclusion of repeated risk factor measurements in CVD risk prediction models improves prediction accuracy and allows predictions to be updated over time. The methods outlined in this paper may provide a useful approach to utilizing available repeated-measures data, such as those readily available in electronic health records, to achieve small gains in risk prediction. Application of these methods to existing data sets, such as primary care records that are more complex than cohort data, and assessment of their clinical utility and cost-effectiveness in these contexts is needed.

In conclusion, incorporating on average 1 postbaseline repeat measurement of SBP, total cholesterol, and HDL cholesterol into CVD risk prediction models can result in slight improvements in risk discrimination and reclassification.

## Supplementary Material

Web Material
