# Supplementary material for: Use of Repeated Blood Pressure and Cholesterol Measurements to Improve Cardiovascular Disease Risk Prediction: An Individual-Participant-Data Meta-Analysis
Source: Am J Epidemiol. 2017 Jun 13;186(8):899–907. doi: 10.1093/aje/kwx149 (PMC5860526; doi:10.1093/aje/kwx149)
Supplement: Web Material [file kwx149paigewebmaterialfinal.pdf]

USE OF REPEATED BLOOD PRESSURE AND CHOLESTEROL MEASUREMENTS TO IMPROVE PREDICTION OF CARDIOVASCULAR DISEASE RISK: AN INDIVIDUAL-PARTICIPANT-DATA META-ANALYSIS

Web Material

Contents

Web Appendix 1: List of study investigators and contributors ... 3

Web Appendix 2: Study names, acronyms, study design and country ... 4

Web Figure 1A: Schematic diagram of the use of the study data in model derivation and validation ... 5

Web Figure 1B: Flow-chart showing the use of the study data in model derivation and validation ... 5

Web Table 1: Summary statistics of included studies ... 6

Web Table 2: Distribution of repeated measurements among included studies ... 7

Web Table 3: Baseline characteristics of participants in the included studies ... 8

Web Figure 2: Scatter plots of repeated blood pressure and cholesterol measures in each study and the cumulative percentage of CVD and censoring events over time ... 9

Web Figure 3: Regression dilution ratio graphs for systolic blood pressure, total cholesterol, and HDL cholesterol ... 22

Web Table 4: Results from the longitudinal models of systolic blood pressure, total cholesterol and HDL cholesterol ... 24

Web Figure 4: Distribution of absolute cardiovascular disease risk scores ... 25

Web Figure 5: Measures of cardiovascular disease risk discrimination in each study ... 26

Web Figure 6: Changes in cardiovascular disease risk discrimination in each study ... 27

Web Table 5: Changes in risk discrimination difference according to mean age, number of repeats and follow-up time in a study ... 28

Web Table 6: Reclassification of events and non-events in the validation data of the models ... 28

Web Figure 7: Changes in cardiovascular disease risk discrimination in each study, using study-specific survival models ... 29

Web Figure 8: Changes in cardiovascular disease risk discrimination in each study, restricted to participants with two or more repeat measures of systolic blood pressure 30

Web Figure 9A: Changes in cardiovascular disease risk discrimination in each study in participants aged 40-69 years... 31

Web Figure 9B: Changes in cardiovascular disease risk discrimination in each study in participants aged ≥70 years... 32

Web Figure 10: Changes in 10-year cardiovascular disease risk discrimination in each study, restricted to studies with 10 years of follow-up... 33

Web Appendix 1. List of study investigators and contributors

Investigators—AFTCAPS: Robert W Tipping; **ALLHAT:** Barry R Davis, Lara Simpson and Sara Pressel; **ARIC:** David Couper, Vijay Nambi, Alvaro Alonso and Seth Martin; **BHS:** Matthew Knuiman; **BRUN:** Johann Willeit, Stefan Kiechl, Peter Santer and Agnes Mayr; **CAPS:** John Gallacher; **CASTEL**: Edoardo Casiglia and Valérie Tikhonoff; **CHARL:** Paul J Nietert, Susan E Sutherland; **CHS:** Bruce M Psaty, Russ Tracy and Mary Cushman; **COPEN:** Børge G Nordestgaard; **CUORE:** Simona Giampaoli, Luigi Palmieri, Salvatore Panico and Diego Vanuzzo; **DESIR:** Ronan Roussel; **DRECE:** Agustin Gómez de la Cámara; **FIA:** Jan-Håkan Jansson, Jonas Andersson and Patrik Wennberg; **FINE_IT:** Chiara Donfrancesco and Simona Giampaoli; **FINRISK:** Veikko Salomaa, Erkki Vartiainen, Pekka Jousilahti and Tiina Laatikainen; **GOH:** Rachel Dankner; **IKNS:** Hiroyasu Iso, Masahiko Kiyama, Akihiko Kitamura and Kazumasa Yamagishi; **ISRAEL:** Uri Goldbourt; **KIHD:** Jukka Salonen, Jussi Kauhanen, Sudhir Kurl and Tomi-Pekka Tuomainen; **LEADER:** Tom W Meade and Jackie A Cooper; **MCVDRFP:** WM Monique Verschuren; **MESA:** Richard A Kronmal, Steven J Shea and Colin Wu; **MGERAUG3:** Annette Peters, Barbara Thorand, Christa Meisinger and Wolfgang Koenig; **MRFIT:** Lewis H Kuller; **OSAKA:** Shinichi Sato, Hiroyasu Iso, Akihiko Kitamura and Kazumasa Yamagishi; **PREVEND:** Stephan J L Bakker, Ron T Gansevoort, Pim van der Harst and Hans L Hillege; **PROCAM:** Gerd Assmann and Helmut Schulte; **QUEBEC:** Gilles R Dagenais, Jean-Pierre Després, Benoît Lamarche and Bernard Cantin; **REYK:** Vilmundur Gudnason, Thor Aspelund, Bolli Thorsson and Gunnar Sigurdsson; **ROTT:** Maryam Kavousi, Oscar H Franco, Albert Hofman and Oscar L. Rueda-Ochoa; **SHIP:** Henry Völzke and André Werner; **SHS:** Lyle G Best, Momotaz Begum and Barbara V Howard; **TARFS**: Altan Onat, Günay Can and Evin Ademoğlu; **TROMSØ:** Inger Njølstad; **ULSAM:** Johan Sundström; **WHITE2:** Eric J Brunner; **ZUTE:** Daan Kromhout and Marianne Geleijnse.

Data Management Team: Thomas Bolton, Sarah Spackman and Matthew Walker.

Coordinating Centre: N Bansal, T Bolton, S Burgess, A S Butterworth, E Di Angelantonio, E Harshfield, S Kaptoge, L Pennells, S Spackman, S G Thompson, M Walker, P Willeit, A M Wood and J Danesh (principal investigator).

Web Appendix 2. Study names, acronyms, study design and country

Acronym	Study Name	Design	Population Source	Location
AFTCAPS	Air Force/Texas Coronary Atherosclerosis Prevention Study	RCT	Popln. Screening	USA
ALLHAT	Antihypertensive and Lipid-Lowering Treatment to Prevent Heart Attack Trial	RCT	Popln. Register	US/Ca/PR/VI
ARIC	Atherosclerosis Risk in Communities Study	Cohort study	Household listings	USA
BHS	Busselton Health Study	Population-based surveys	Electoral roll	Australia
BRUN	Bruneck Study	Cohort study	Popln. Register	Italy
CaPS	Caerphilly Prospective Study	Cohort study	Electoral rolls	UK
CASTEL	Cardiovascular Study in the Elderly	Cohort study	Popln. Screening	Italy
CHARL	Charleston Heart Study	Cohort study	Household listing	USA
CHS	Cardiovascular Health Study	Cohort study	Medicare lists	USA
COPEN	Copenhagen City Heart Study	Cohort study	Popln. Register	Denmark
CUORE	Progetto CUORE	Series of cohort studies	Electoral roll	Italy
DESIR	Data from an Epidemiological Study on the Insulin Resistance Syndrome	Cohort study	Health check-up	France
DRECE	Diet and Risk of Cardiovascular Disease in Spain	Cohort study	General	Spain
FIA	First Myocardial Infarction in Northern Sweden	Cohort study	Popln. Register	Sweden
FINE_IT	Finland, Italy and Netherlands Elderly Study – Italian cohort	Cohort study	Survivors of existing cohort	Italy
FINRISK	Finrisk Cohort	Cohort study	Popln. Register	Finland
GOH	Glucose Intolerance, Obesity and Hypertension Study	Cohort study	Popln. Register	Israel
IKNS	Ikawa, Kyowa, Noichi Study	Cohort study	Popln. Screening	Japan
ISRAEL	Israeli Ischaemic Heart Disease Study	Cohort study	Occupational	Israel
KIHD	Kuopio Ischaemic Heart Disease Study	Cohort study	Popln. Register	Finland
LEADER	Lower Extremity Arterial Disease Event Reduction Trial	RCT	GP listings	UK
MCVDRFP	Monitoring of CVD Risk Factors Project	Cohort study	General	The Netherlands
MESA	Multi-Ethnic Study of Atherosclerosis	Cohort study	General	USA
MOGERAUG3	MONICA/KORA Augsburg Survey 3	Cohort study	Popln. Register	Germany
MRFIT	Multiple Risk Factor Intervention Trial	RCT	Popln. Screening	USA
OSAKA	Osaka Study	Cohort study	Occupational & Popln. Register	Japan
PREVEND	Prevention of Renal and Vascular End Stage Disease Study	Cohort study	Popln. Register	The Netherlands
PROCAM	Prospective Cardiovascular Münster Study	Cohort study	Occupational	Germany
QUEBEC	Quebec Cardiovascular Study	Cohort study	Popln. Register	Canada
REYK	Reykjavik Study	Cohort study	Popln. Register	Iceland
ROTT/RS-I	Rotterdam Study	Cohort study	Popln. Register	The Netherlands
SHIP	Study of Health in Pomerania	Cohort study	General	Germany
SHS	Strong Heart Study	Cohort study	Tribal rolls	USA
TARFS	Turkish Adult Risk Factor Study	Cohort study	Household listings	Turkey
TROMSØ	Tromsø Study	Cohort study	Household listings	Norway
ULSAM	Uppsala Longitudinal Study of Adult Men	Cohort study	Popln. Register	Sweden
WHITEII	Whitehall II Study	Cohort study	Civil servant	UK
ZUTE	Zutphen Elderly Study	Cohort study	Popln. Register	The Netherlands

Web Figure 1A. Schematic diagram of the use of the study data in model derivation and validation

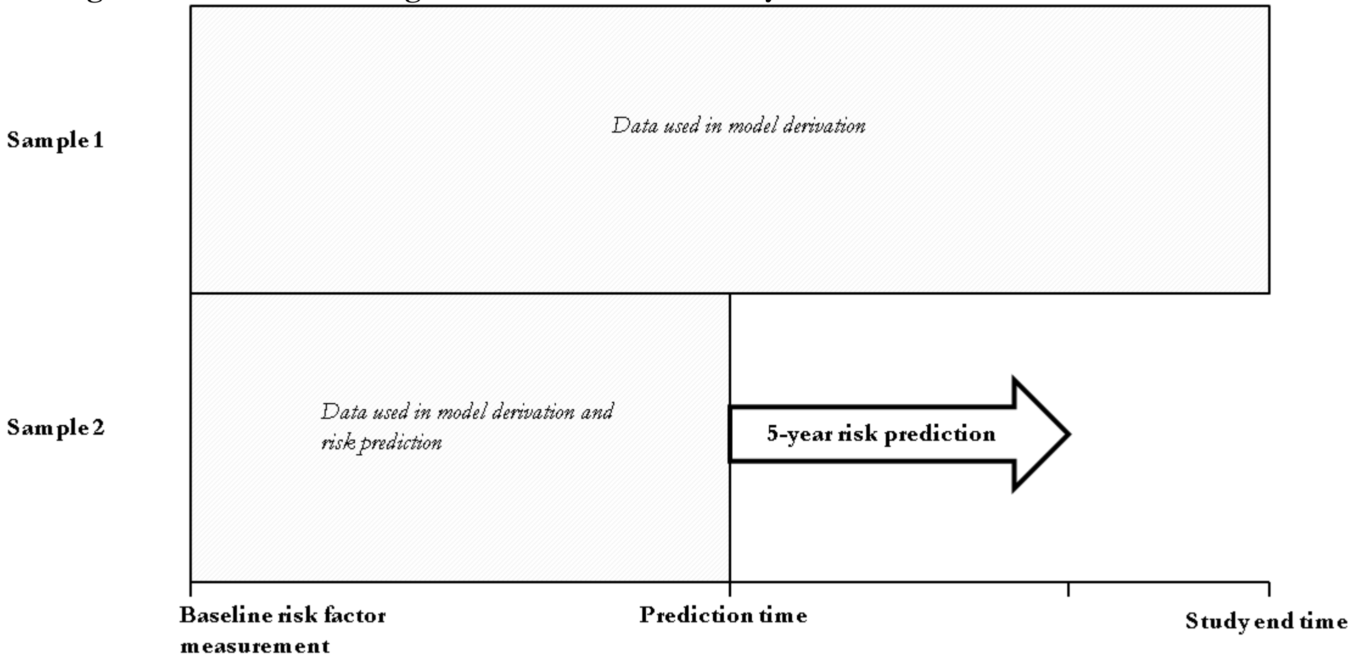

Notes: Data from studies were split into two 50% samples, with data from the second sample censored at the prediction time. Data from both samples were used to derive the longitudinal models while data from the first sample was used to derive the Cox proportional hazards models. Risk prediction and validation were done using data from the second sample only.

Web Figure 1B. Flow-chart showing the use of the study data in model derivation and validation

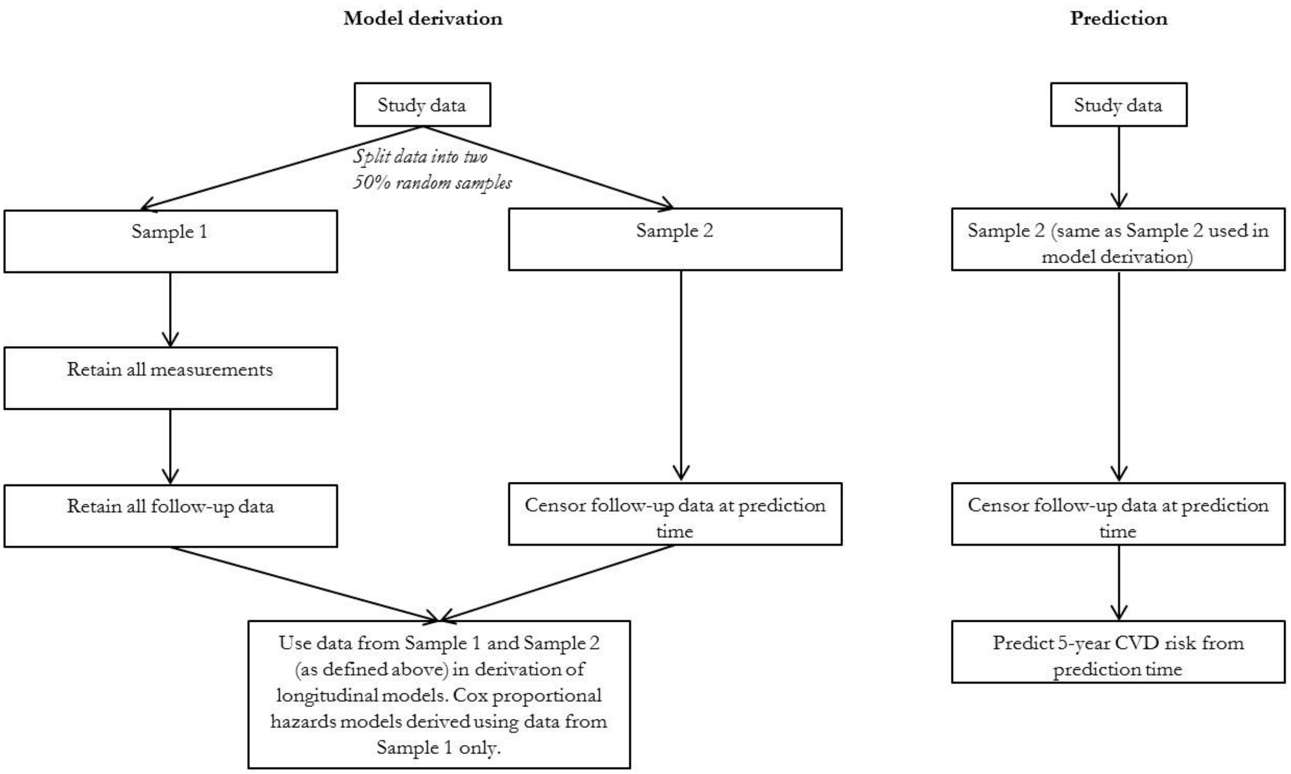

Web Table 1. Summary statistics of included studies

	No.	No. of CVD Events	Total Events per 1,000 Person-Years	Follow-up Time, years	
				Mean	Max
AFTCAPS	5,439	155	5.4	5.3	7.3
ALLHAT	14,098	813	14.0	4.1	8.0
ARIC	13,161	2,342	9.4	19.0	25.1
BHS	2,915	431	8.7	16.9	24.2
BRUN	794	143	10.6	17.0	20.5
CaPS	2,107	285	11.6	11.7	13.5
CASTEL	2,066	361	16.8	10.4	14.0
CHARL	964	444	21.5	21.5	47.0
CHS	3,769	972	26.1	9.9	13.1
COPEN	6,559	1,479	16.9	13.4	18.8
CUORE	12,605	733	5.3	10.9	19.5
DESIR	2,859	36	1.4	8.8	10.4
DRECE	1,357	36	1.4	18.3	19.7
FIA	660	156	38.9	6.1	13.7
FINE_IT	428	190	39.6	11.2	21.5
FINRISK	3,580	268	5.2	14.5	17.0
GOH	1,186	68	3.1	18.8	29.4
IKNS	7,211	385	5.1	10.5	18.6
ISRAEL	6,548	855	6.5	20.1	24.0
KIHD	2,036	578	15.9	17.9	25.8
LEADER	865	161	46.4	4.0	7.8
MCVDRFP	13,218	410	1.9	16.1	20.0
MESA	6,467	252	5.1	7.7	10.9
MOGERAUG3	3,178	275	7.1	12.2	15.2
MRFIT	3,115	248	11.2	7.1	8.3
OSAKA	10,242	173	1.8	9.6	18.8
PREVEND	5,325	278	5.3	9.8	11.3
PROCAM	13,062	685	4.9	10.7	26.8
QUEBEC	1,592	239	10.0	15.0	19.0
REYK	14,168	3,932	12.0	23.1	37.2
ROTT/RS-I	4,112	527	11.5	11.1	14.7
SHIP	1,391	54	3.6	10.8	14.6
SHS	2,643	502	17.8	10.7	14.6
TARFS	1,812	60	4.9	6.8	14.4
TROMSO	9,413	1,280	9.0	15.1	19.4
ULSAM	1,794	776	19.4	22.3	38.7
WHITEII	8,076	327	3.4	11.9	17.3
ZUTE	630	261	43.0	9.6	15.3
TOTAL	191,445	21,170	8.6	12.8	47.0

Web Table 2. Distribution of repeated measurements among included studies

	Systolic Blood Pressure					Total Cholesterol					HDL Cholesterol				
	Post-Baseline Repeats			Years Between Repeats		Post-Baseline Repeats			Years Between Repeats		Post-Baseline Repeats			Years Between Repeats	
	# with ≥1	Mean	Max	Mean (SD)	Range	# with ≥1	Mean	Max	Mean (SD)	Range	# with ≥1	Mean	Max	Mean (SD)	Range
AFTCAPS	5,431	4	6	1.0 (0.1)	0.6-2.3	5,427	1	1	0.9 (0.1)	0.7-1.2	5,427	1	1	0.9 (0.1)	0.7-1.2
ALLHAT	12,492	3	7	0.9 (0.2)	0.1-1.0	10,073	1	4	1.0 (0.1)	0.2-1.0	866	0	3	1.0 (0.1)	0.4-1.0
ARIC	12,205	2	3	3.0 (0.5)	0.6-11.1	12,182	2	3	3.0 (0.5)	0.6-11.1	12,180	2	3	3.0 (0.5)	0.6-11.1
BHS	1,826	1	3	6.3 (4.0)	3.0-17.0	1,838	1	3	6.3 (4.0)	3.0-17.0	1,537	1	2	9.2 (3.5)	3.0-17.0
BRUN	731	2	3	5.0 (0.4)	4.8-10.1	731	2	3	5.1 (0.4)	4.8-10.1	731	2	3	5.1 (0.4)	4.8-10.1
CaPS	1,652	1	2	5.0 (0.4)	3.4-11.4	1,618	1	2	5.0 (0.3)	3.2-7.8	1,556	1	1	5.0 (0.2)	3.8-7.7
CASTEL	1,046	0	1	5.4 (1.0)	3.8-7.0	1,058	0	1	5.4 (1.0)	3.8-7.0	1,001	0	1	5.4 (1.0)	3.8-7.0
CHARL	588	1	4	9.4 (3.6)	0.1-22.5	574	1	3	7.0 (4.7)	0.1-12.2	302	0	2	5.5 (3.7)	0.1-11.3
CHS	3,608	6	8	1.0 (0.1)	0.1-7.0	3,114	1	2	1.0 (0.2)	0.1-4.0	2,746	1	1	1.0 (0.2)	0.2-3.8
COPEN	3,309	1	1	9.4 (0.4)	7.8-11.5	3,248	0	1	9.4 (0.4)	7.8-11.5	3,244	0	1	9.4 (0.4)	7.8-11.5
CUORE	2,624	0	2	6.0 (2.5)	2.6-12.3	2,614	0	2	6.0 (2.5)	2.6-12.3	2,612	0	2	6.0 (2.5)	2.6-12.3
DESIR	2,747	2	3	3.0 (0.2)	1.9-6.1	2,751	2	3	3.0 (0.2)	1.9-6.1	2,743	2	3	3.0 (0.2)	1.9-6.1
DRECE	367	0	1	14.6 (0.4)	13.8-15.1	402	0	1	14.6 (0.4)	13.8-15.1	395	0	1	14.6 (0.4)	13.8-15.1
FIA	42	0	1	5.6 (3.1)	1.0-10.1	42	0	1	5.6 (3.1)	1.0-10.1	10	0	1	6.6 (2.1)	5.1-10.0
FINE_IT	264	1	3	5.6 (1.7)	3.6-15.2	262	1	3	5.5 (1.6)	3.6-15.0	262	1	3	5.5 (1.6)	3.6-15.0
FINRISK	428	0	1	3.1 (0.1)	3.0-3.8	428	0	1	3.1 (0.1)	3.0-3.4	428	0	1	3.1 (0.1)	3.0-3.4
GOH	362	0	1	22.1 (2.5)	17.2-28.4	359	0	1	22.2 (2.4)	17.4-28.4	359	0	1	22.2 (2.4)	17.4-28.4
IKNS	5,717	4	18	1.8 (1.3)	0.8-17.0	5,854	4	18	1.8 (1.4)	0.8-17.0	5,675	3	17	1.9 (1.4)	0.85-17.0
ISRAEL	6,415	2	2	2.4 (0.5)	0.8-3.8	4,715	1	1	2.0 (0.3)	0.8-3.4	4,364	1	1	2.0 (0.3)	0.8-3.2
KIHD	798	1	2	5.4 (1.4)	3.6-9.9	797	1	2	5.4 (1.4)	3.6-9.9	797	1	2	5.4 (1.4)	3.6-9.9
LEADER	669	2	4	0.6 (0.2)	0.2-3.1	671	2	4	0.6 (0.2)	0.2-3.1	669	2	4	0.6 (0.2)	0.2-3.1
MCVDRFP	236	0	1	2.8 (0.8)	0.2-4.2	236	0	1	2.8 (0.8)	0.2-4.2	236	0	1	2.8 (0.8)	0.2-4.2
MESA	6,040	3	4	1.6 (0.4)	0.4-6.6	6,029	3	4	1.6 (0.4)	0.4-6.6	6,029	3	4	1.6 (0.4)	0.4-6.6
MOGERAUG3	1,853	1	1	9.9 (0.2)	9.4-10.2	1,859	1	1	9.9 (0.2)	9.5-10.2	1,859	1	1	9.9 (0.2)	9.5-10.2
MRFIT	3,115	7	8	0.9 (0.4)	0.1-7.4	3,035	6	7	1.0 (0.3)	0.2-7.4	2,938	3	3	1.0 (0.3)	0.3-7.1
OSAKA	9,188	5	18	1.1 (0.6)	0.1-16.0	9,189	5	18	1.1 (0.6)	0.1-16.0	9,038	5	16	1.1 (0.6)	0.1-16.0
PREVEND	4,444	4	21	1.6 (1.6)	0.0-7.3	4,438	3	10	2.3 (1.6)	0.0-7.3	4,427	3	10	2.3 (1.6)	0.0-7.3
PROCAM	3,105	1	32	3.1 (2.6)	0.2-17.2	3,105	1	32	3.1 (2.6)	0.2-17.2	3,105	1	32	3.1 (2.6)	0.2-17.2
QUEBEC	686	0	1	3.6 (0.3)	3.1-4.7	612	0	1	3.5 (0.2)	3.1-4.4	612	0	1	3.5 (0.2)	3.1-4.4
REYK	2,405	0	5	5.6 (2.2)	1.1-26.8	2,450	0	5	5.6 (2.2)	1.1-26.8	842	0	5	6.0 (2.2)	2.5-26.8
ROTT/RS-I	2,891	1	1	6.5 (0.3)	3.5-9.5	2,801	1	1	6.5 (0.3)	5.3-9.5	2,753	1	1	6.4 (0.3)	5.3-9.5
SHIP	1,322	1	1	5.1 (0.4)	4.4-8.0	1,321	1	1	5.1 (0.4)	4.4-8.0	1,321	1	1	5.1 (0.4)	4.4-8.0
SHS	2,178	1	2	4.1 (1.0)	1.6-10.1	2,173	1	2	4.1 (1.0)	1.6-9.8	2,173	1	2	4.1 (1.0)	1.6-9.8
TARFS	1,478	2	6	1.9 (0.5)	1.0-2.3	1,412	2	5	1.9 (0.4)	1.0-2.3	1,412	2	5	1.9 (0.4)	1.0-2.3
TROMSO	6,606	1	1	8.1 (0.2)	7.3-8.8	6,597	1	1	8.1 (0.2)	7.4-8.8	6,591	1	1	8.1 (0.2)	7.4-8.8
ULSAM	1,357	1	4	9.1 (3.0)	3.1-28.5	1,070	1	4	8.5 (3.5)	3.1-28.5	970	1	4	8.2 (3.7)	3.1-28.5
WHITEII	1,280	0	1	4.6 (0.3)	3.7-5.8	6,198	1	2	2.6 (1.0)	1.3-5.8	5,671	1	2	2.6 (1.1)	1.3-5.8
ZUTE	419	1	1	5.0 (0.1)	4.6-5.2	419	1	1	5.0 (0.1)	4.6-5.2	419	1	1	5.0 (0.1)	4.6-5.2
TOTAL	111,924	1.8	32	2.3 (2.3)	0.01-28.5	111,702	1.4	32	2.6 (2.4)	0.0-28.5	98,300	1.2	32	2.7 (2.4)	0.0-28.5

Web Table 3. Baseline characteristics of participants in the included studies

	Males (%)	Mean Age, years (SD)	Current Smokers (%)	History of Diabetes (%)	Mean Systolic Blood Pressure, mm Hg (SD)	Mean Total Cholesterol, mmol/L (SD)	Mean HDL Cholesterol, mmol/L (SD)
AFTCAPS	4,624 (85)	58.2 (7.2)	638 (12)	121 (2)	137.9 (17.1)	5.7 (0.5)	1.0 (0.1)
ALLHAT	7,102 (50)	64.7 (6.4)	3,966 (28)	6,395 (45)	145.9 (14.9)	5.6 (1.1)	1.2 (0.4)
ARIC	5,924 (45)	54.4 (5.7)	3,671 (28)	1,407 (11)	120.8 (18.5)	5.5 (1.1)	1.4 (0.4)
BHS	1,357 (47)	57.4 (10.2)	625 (21)	132 (5)	134.7 (20.9)	6.1 (1.2)	1.5 (0.4)
BRUN	387 (49)	57.4 (11.2)	193 (24)	28 (4)	145.1 (21.6)	5.7 (1.0)	1.5 (0.4)
CaPS	2,107 (100)	52.1 (4.6)	1,153 (55)	30 (1)	141.0 (19.3)	5.7 (1.1)	1.1 (0.3)
CASTEL	819 (40)	71.9 (3.6)	294 (14)	268 (13)	159.4 (24.3)	5.7 (1.1)	1.5 (0.4)
CHARL	493 (51)	56.5 (10.3)	442 (46)	92 (10)	140.5 (24.0)	6.3 (1.3)	1.4 (0.4)
CHS	1,398 (37)	71.0 (3.8)	499 (13)	526 (14)	135.3 (21.1)	5.5 (1.0)	1.4 (0.4)
COPEN	2,756 (42)	61.1 (10.3)	3,318 (51)	214 (3)	141.0 (22.0)	6.3 (1.2)	1.6 (0.5)
CUORE	3,894 (31)	52.2 (7.5)	3,880 (31)	458 (4)	139.7 (22.1)	6.0 (1.2)	1.5 (0.4)
DESIR	1,394 (49)	52.3 (7.2)	432 (15)	150 (5)	133.1 (15.8)	5.9 (0.9)	1.7 (0.4)
DRECE	655 (48)	49.6 (6.1)	360 (27)	145 (11)	126.9 (18.7)	5.6 (1.1)	1.4 (0.4)
FIA	494 (75)	55.2 (7.4)	195 (30)	20 (3)	137.9 (19.1)	6.7 (1.2)	1.3 (0.4)
FINE_IT	428 (100)	71.7 (3.8)	117 (27)	36 (8)	166.7 (22.0)	5.9 (1.1)	1.3 (0.3)
FINRISK	1,661 (46)	51.7 (7.0)	932 (26)	89 (2)	140.8 (20.2)	5.9 (1.1)	1.4 (0.4)
GOH	578 (49)	56.2 (9.8)	292 (25)	158 (13)	136.1 (21.8)	5.7 (1.3)	1.2 (0.3)
IKNS	2,959 (41)	58.8 (10)	1,643 (23)	550 (8)	134.6 (18.9)	5.2 (0.9)	1.5 (0.4)
ISRAEL	6,548 (100)	49.8 (6.8)	2,689 (41)	311 (5)	135.7 (20.4)	5.3 (1.0)	1.0 (0.3)
KIHD	2,036 (100)	52.5 (5.3)	621 (31)	89 (4)	130.7 (17.1)	5.9 (1.1)	1.3 (0.3)
LEADER	865 (100)	66.4 (8.1)	360 (42)	137 (16)	149.1 (21.8)	5.7 (0.9)	1.2 (0.4)
MCVDRFP	6,106 (46)	49.5 (5.8)	5,700 (43)	184 (1)	123.4 (16.7)	5.9 (1.1)	1.3 (0.3)
MESA	3,062 (47)	61.3 (9.6)	996 (15)	805 (12)	125.7 (21.1)	5.0 (0.9)	1.3 (0.4)
MOGERAUG3	1,566 (49)	56.5 (9.7)	663 (21)	197 (6)	136.7 (19.8)	6.2 (1.1)	1.4 (0.4)
MRFIT	3,115 (100)	48.4 (4.8)	1,868 (60)	96 (3)	139.8 (14.5)	6.4 (0.9)	1.1 (0.3)
OSAKA	6,770 (66)	54.3 (8.6)	3,765 (37)	604 (6)	124.6 (17.9)	5.4 (0.9)	1.5 (0.4)
PREVEND	2,678 (50)	54.2 (9.8)	1,783 (33)	241 (5)	132.1 (20.9)	5.9 (1.1)	1.3 (0.4)
PROCAM	9,704 (74)	49.6 (6.0)	4,541 (35)	369 (3)	132.8 (19.1)	5.9 (1.1)	1.3 (0.4)
QUEBEC	1,592 (100)	54.4 (7.3)	595 (37)	98 (6)	129.8 (16.5)	5.8 (1.0)	1.0 (0.3)
REYK	6,686 (47)	53.7 (7.5)	6,479 (46)	309 (2)	139.7 (21.9)	6.5 (1.2)	1.4 (0.2)
ROTT/RS-I	1,625 (40)	65.7 (6.4)	828 (20)	319 (8)	137.4 (21.6)	6.7 (1.2)	1.4 (0.4)
SHIP	673 (48)	54.1 (8.9)	324 (23)	117 (8)	138.3 (19.9)	6.0 (1.2)	1.5 (0.4)
SHS	1,077 (41)	56.5 (8.1)	1,079 (41)	1,022 (39)	126.1 (18.9)	5.1 (1.0)	1.2 (0.4)
TARFS	875 (48)	53.2 (9.1)	455 (25)	179 (10)	134.0 (24.7)	5.0 (1.0)	1.1 (0.3)
TROMSO	4,804 (51)	51.0 (9.8)	4,246 (45)	125 (1)	134.6 (19.2)	6.4 (1.2)	1.5 (0.4)
ULSAM	1,794 (100)	54.2 (8.7)	984 (55)	128 (7)	135.8 (19.0)	6.8 (1.4)	1.3 (0.4)
WHITEII	5,537 (69)	49.0 (5.5)	1,684 (21)	102 (1)	120.7 (13.8)	6.4 (1.2)	1.4 (0.4)
ZUTE	630 (100)	70.5 (3.9)	209 (33)	60 (10)	150.7 (20.9)	6.1 (1.1)	1.1 (0.3)
TOTAL	106,773 (56)	55.2 (9.5)	62,519 (33)	16,311 (9)	133.9 (20.9)	5.9 (1.2)	1.3 (0.4)

Web Figure 2. Scatter plots of repeated blood pressure and cholesterol measures in each study and the cumulative percentage of CVD and censoring events over time

AFTCAPS

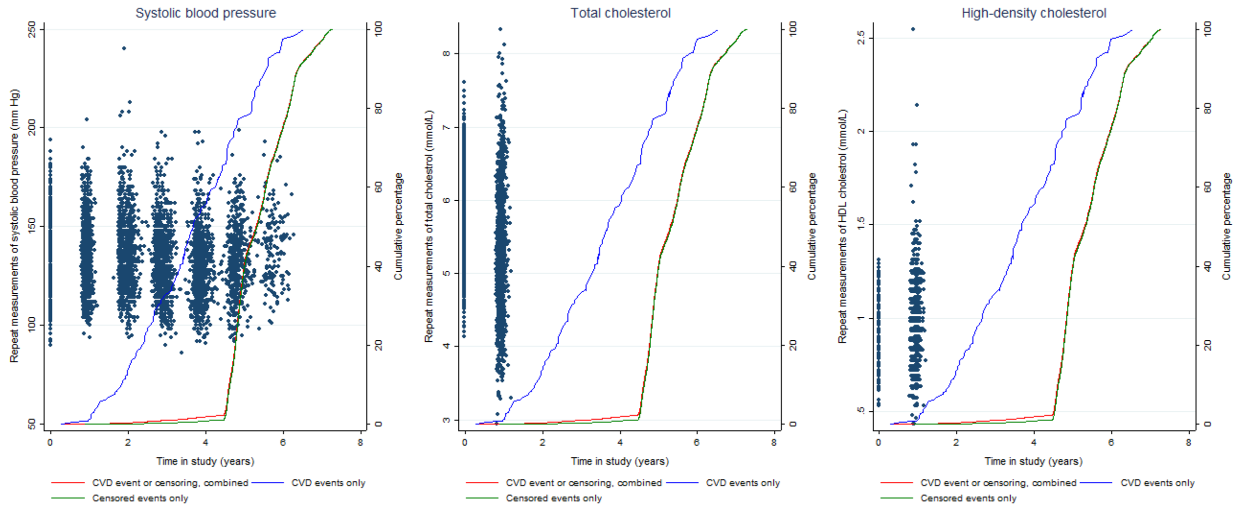

ALLHAT

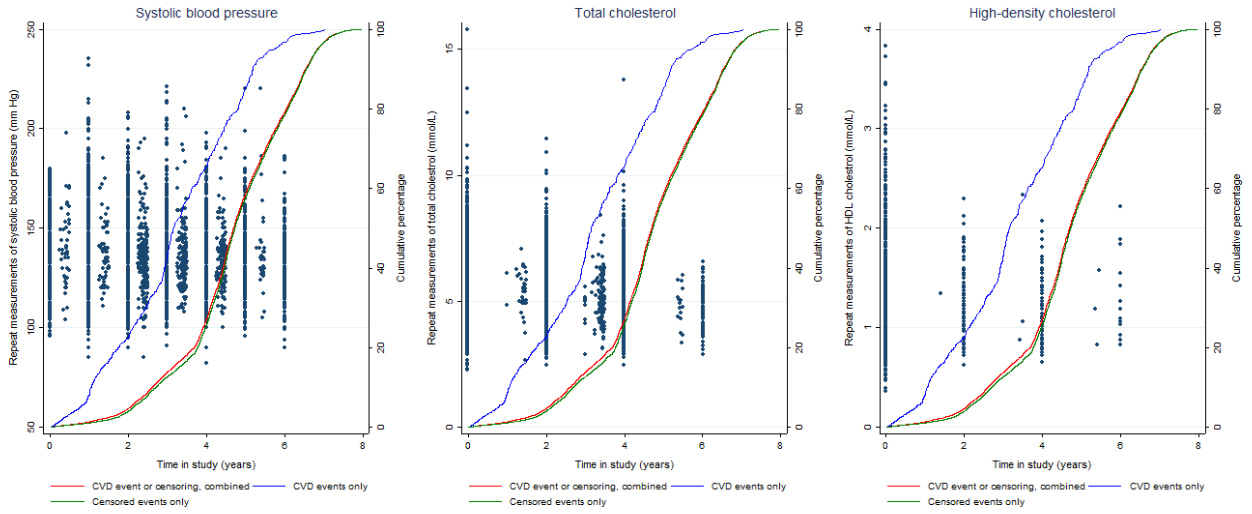

ARIC

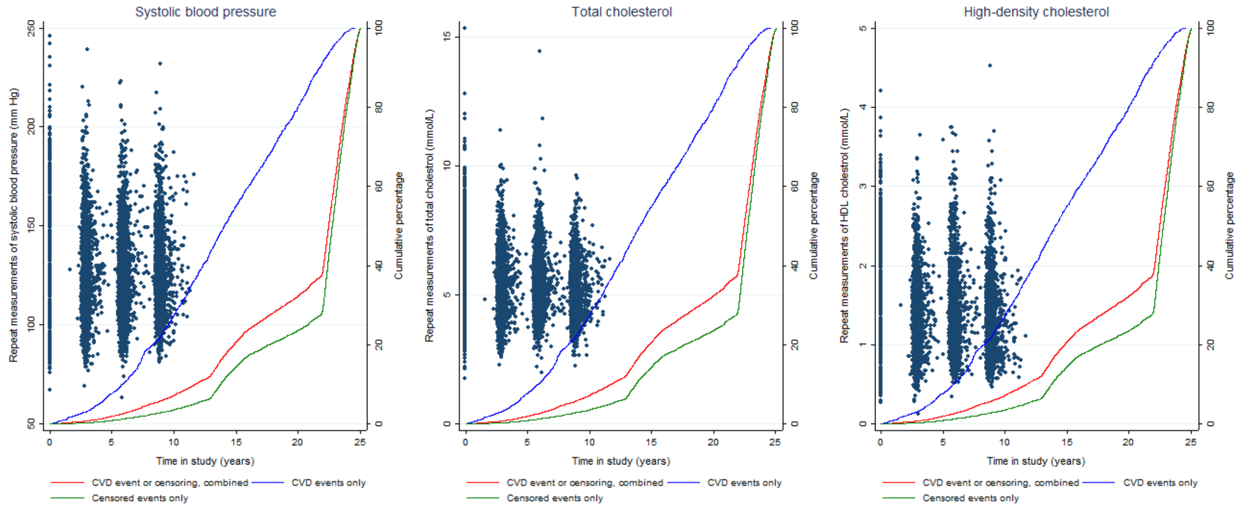

BHS

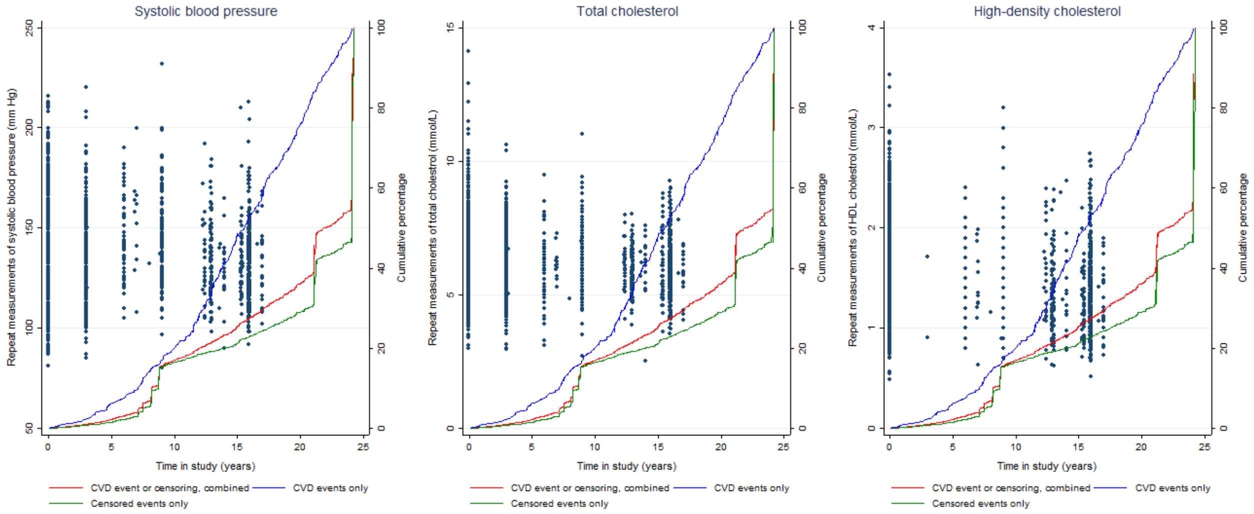

BRUN

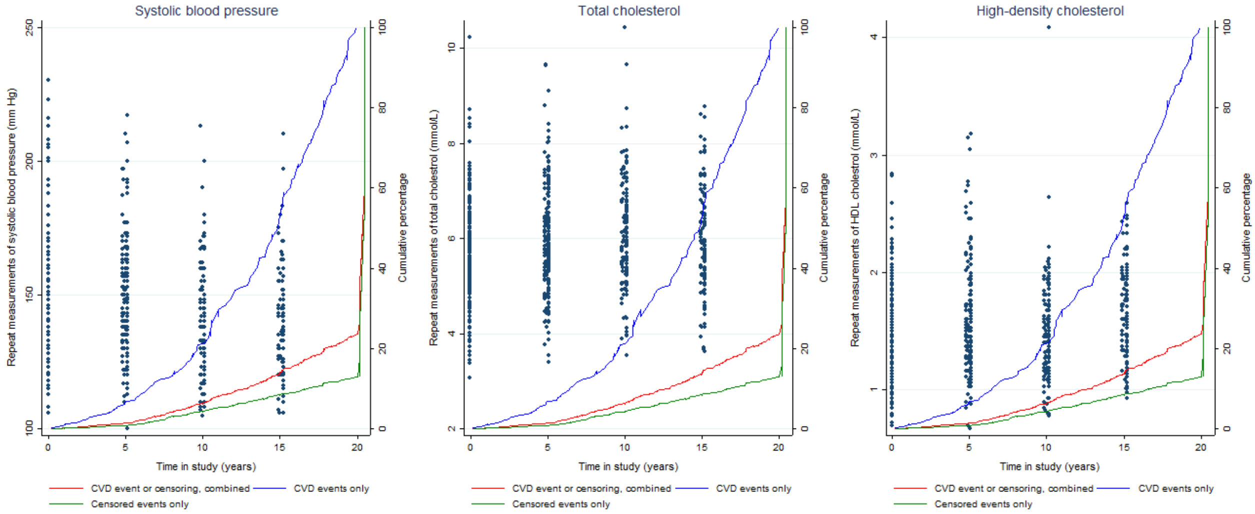

CaPS

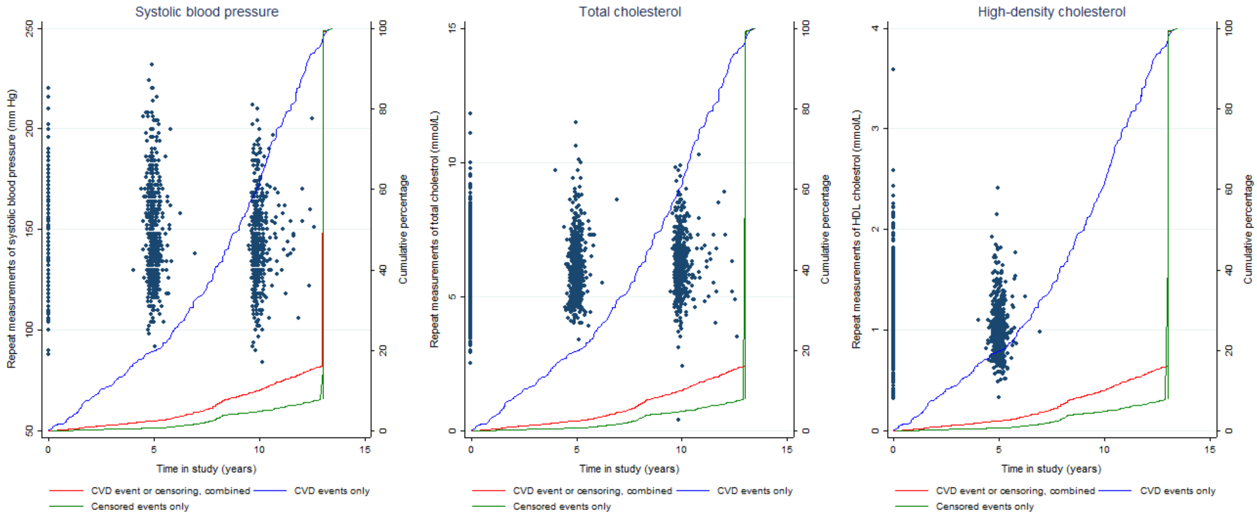

CASTEL

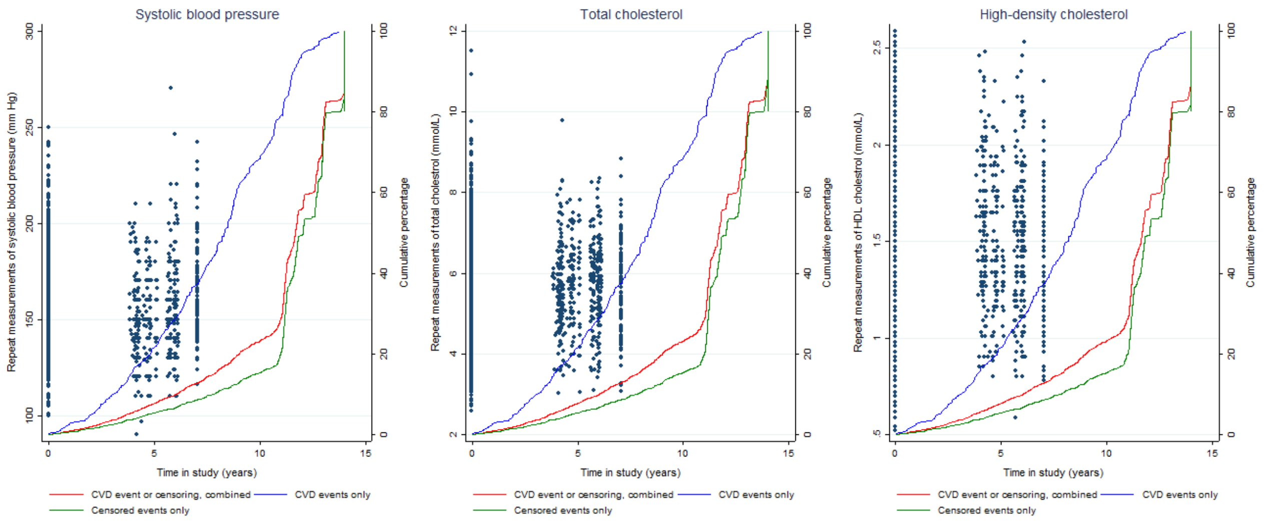

CHARL

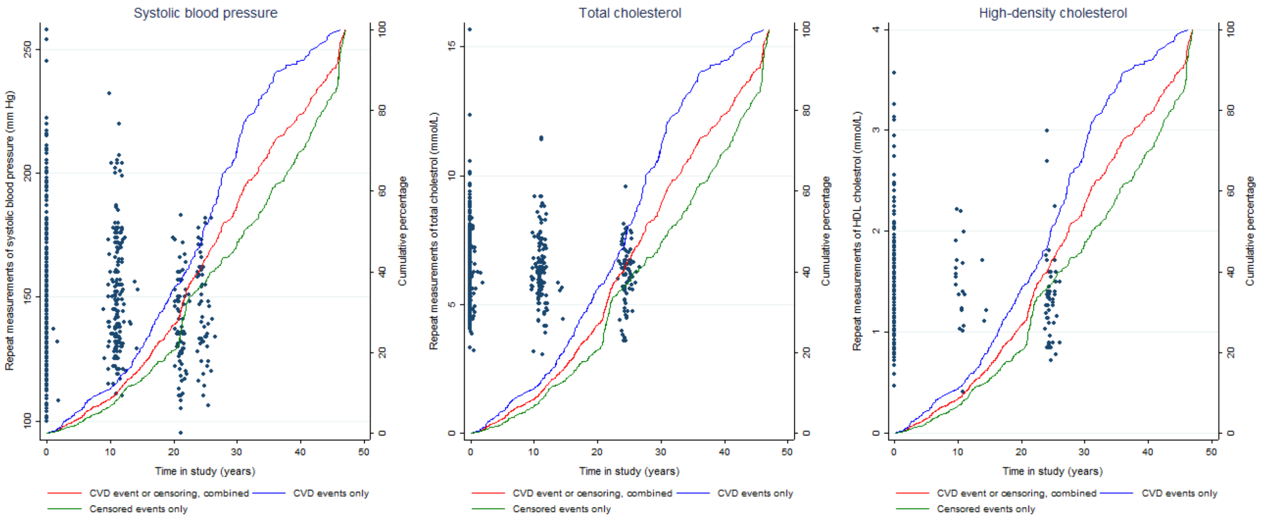

CHS

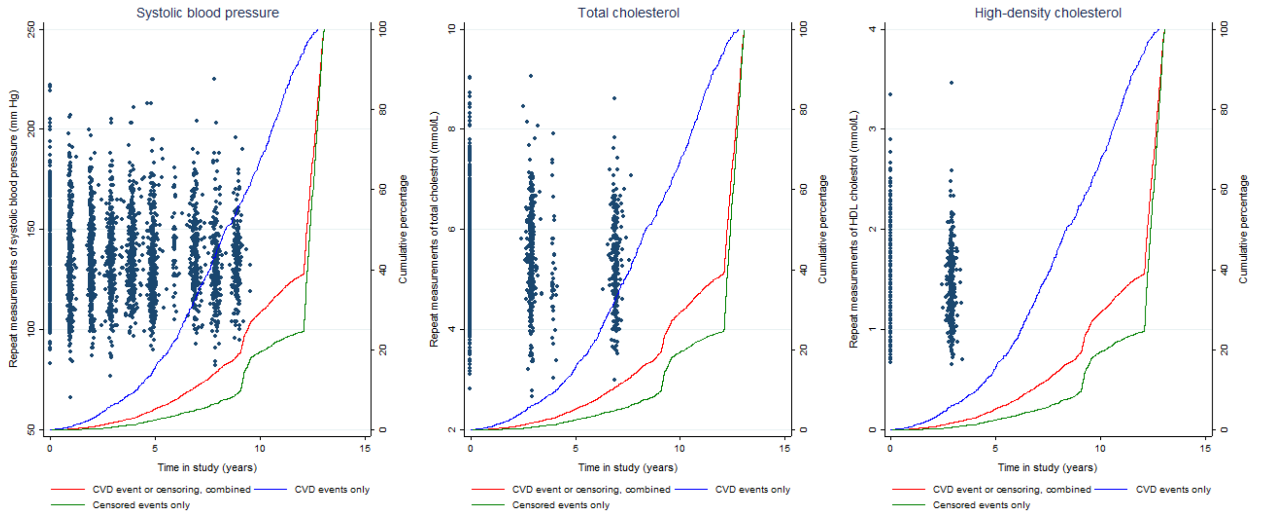

COPEN

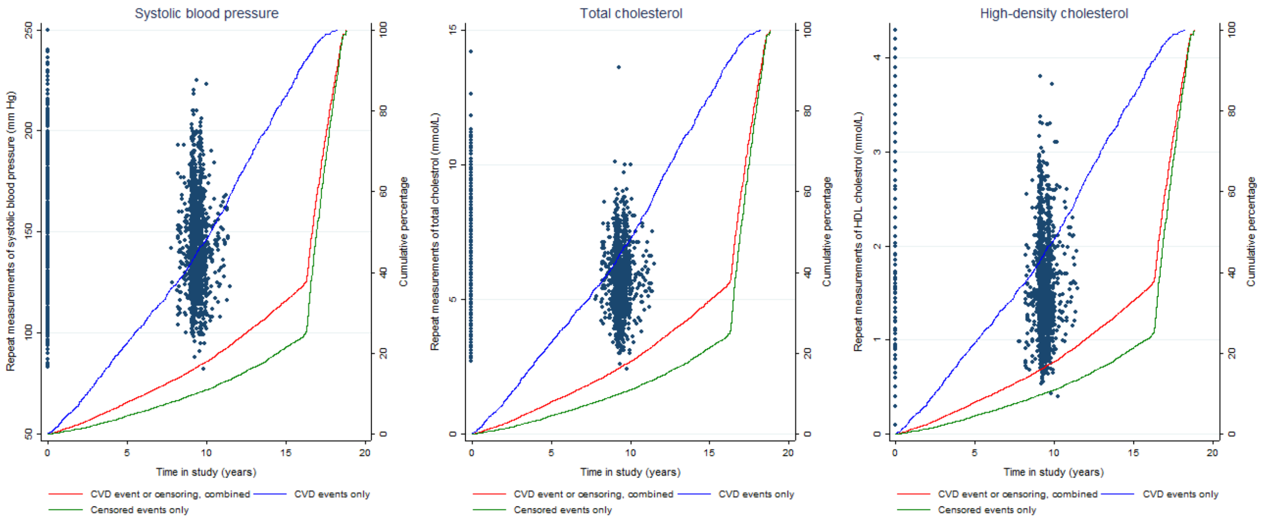

CUORE

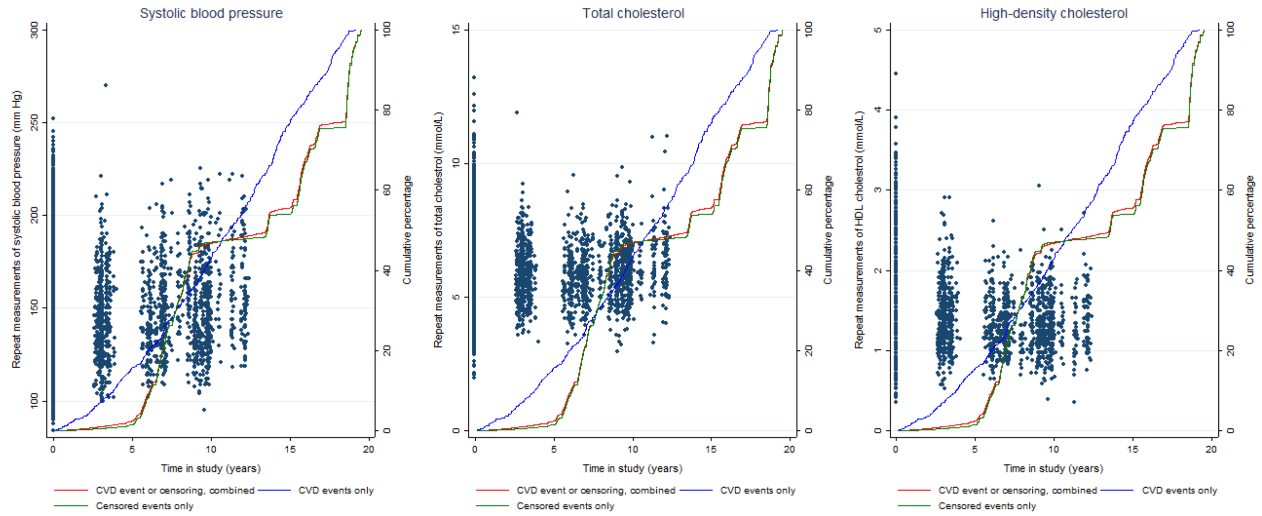

DESIR

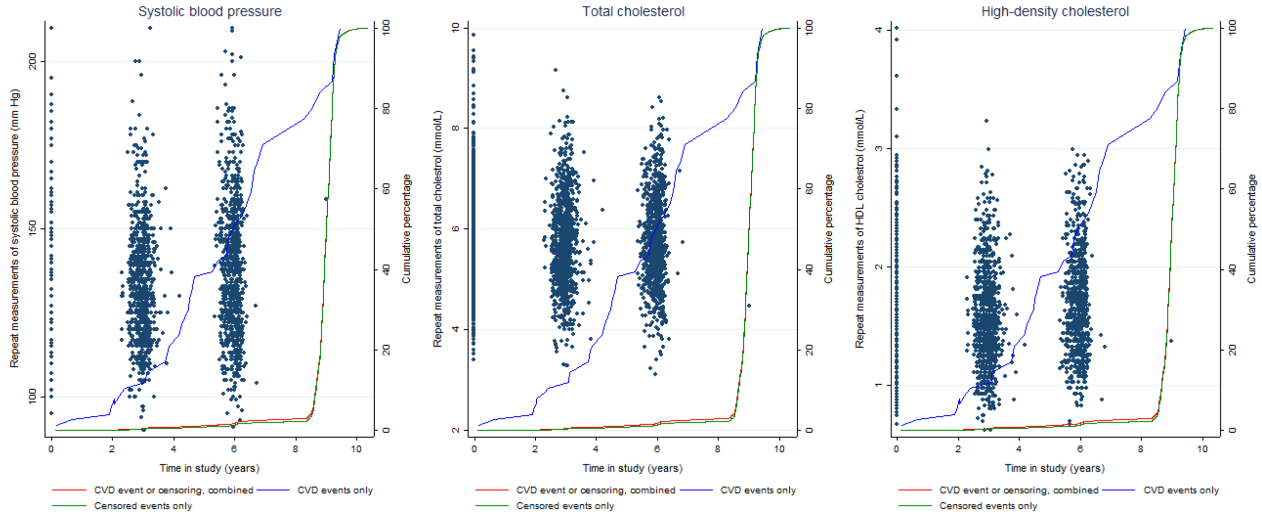

DRECE

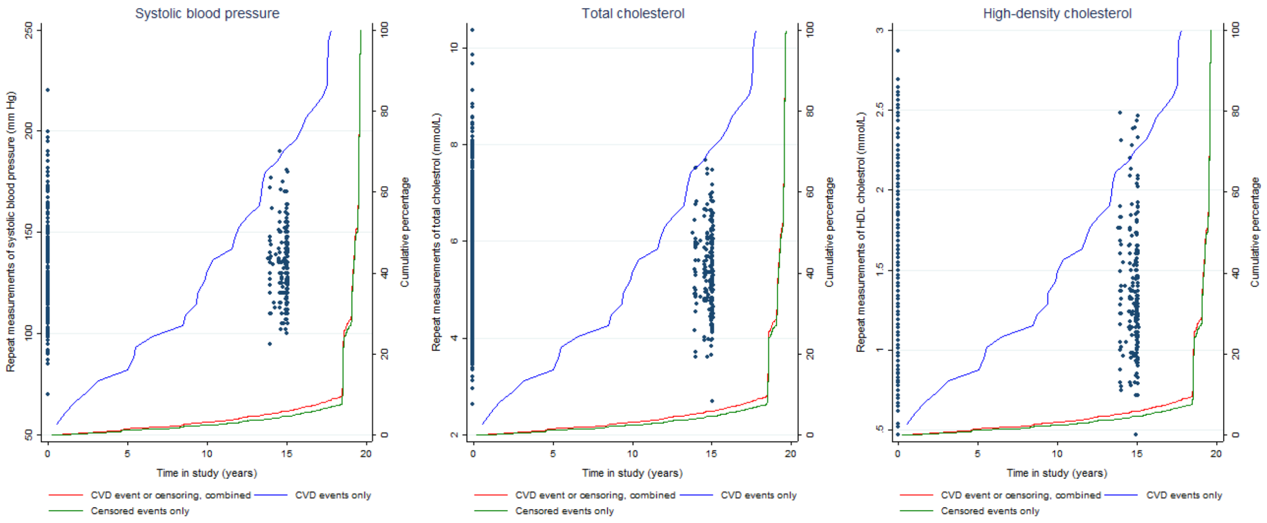

FIA

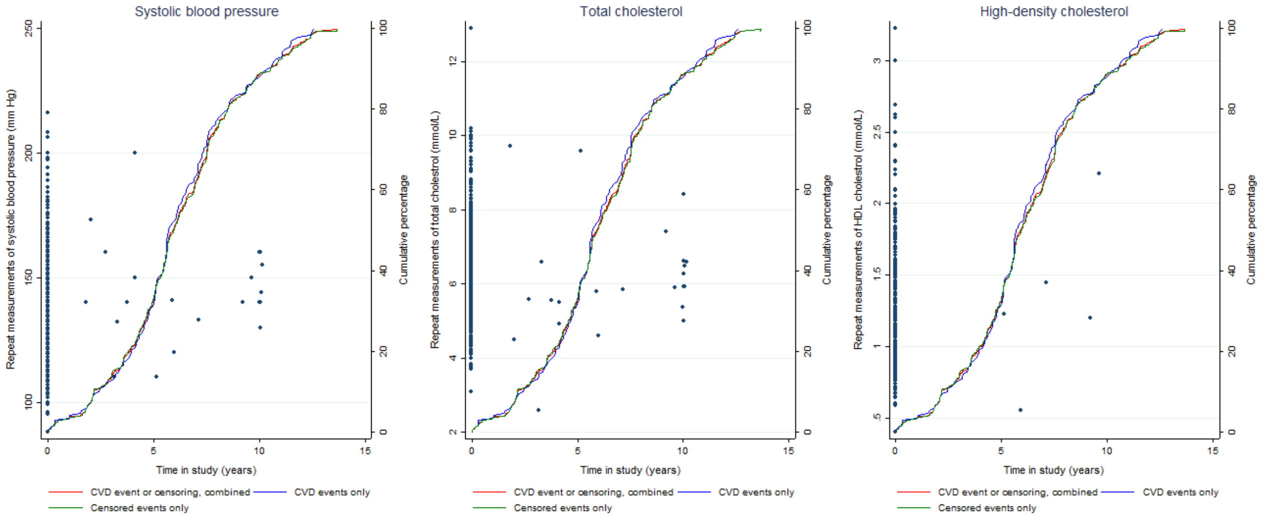

FINE_IT

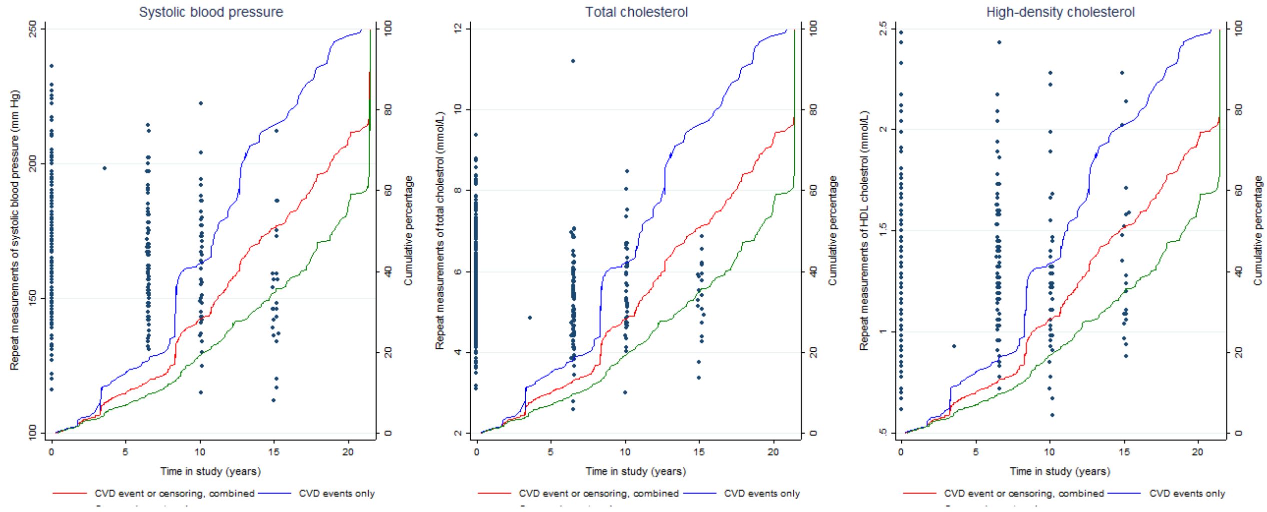

FINRISK

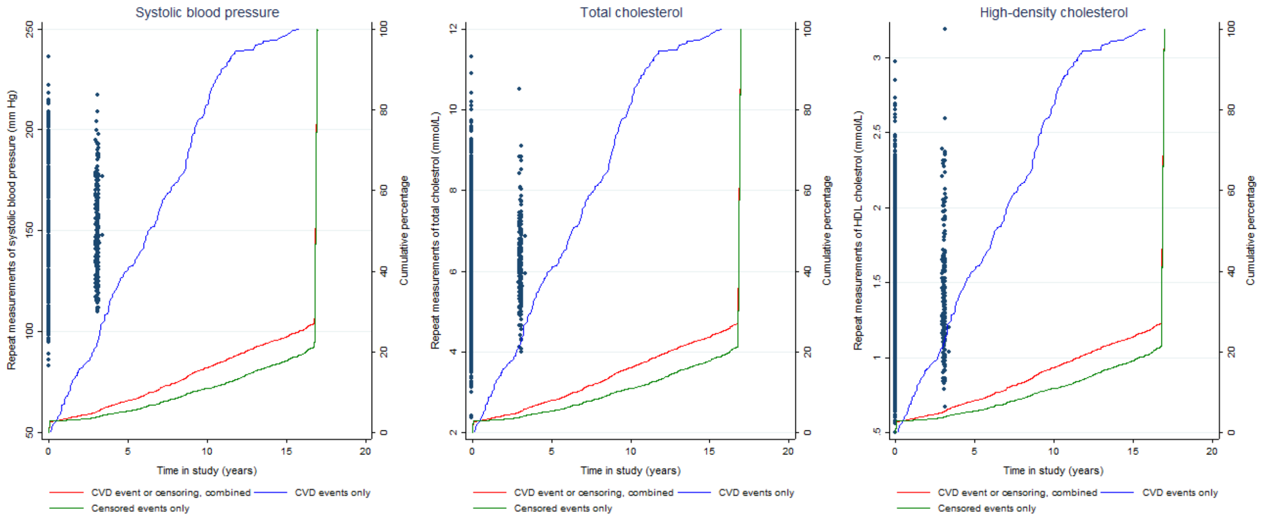

GOH

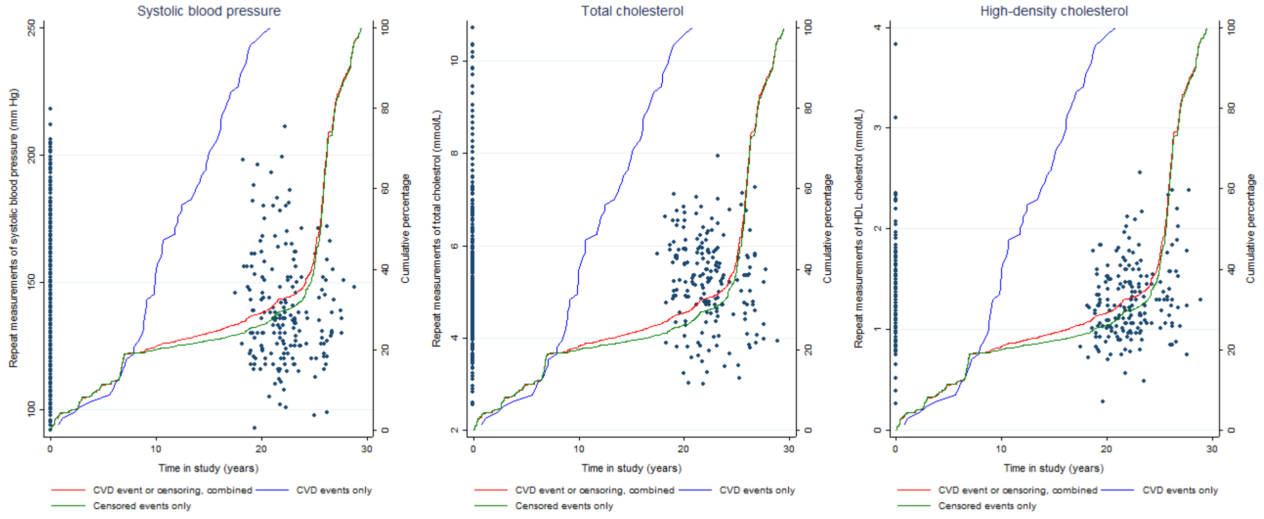

IKNS

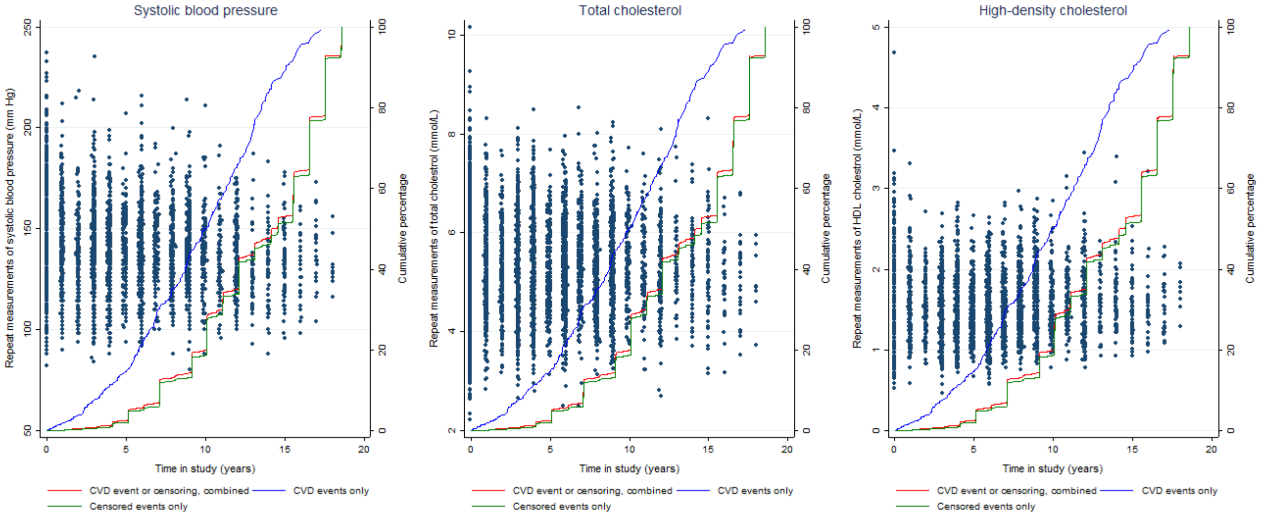

ISRAEL

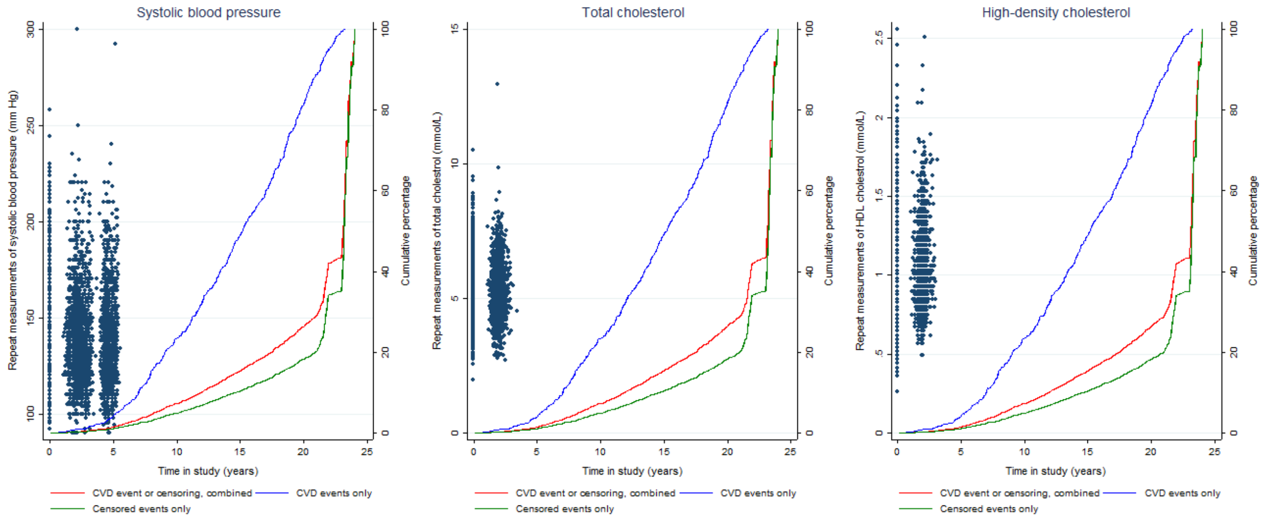

KIHD

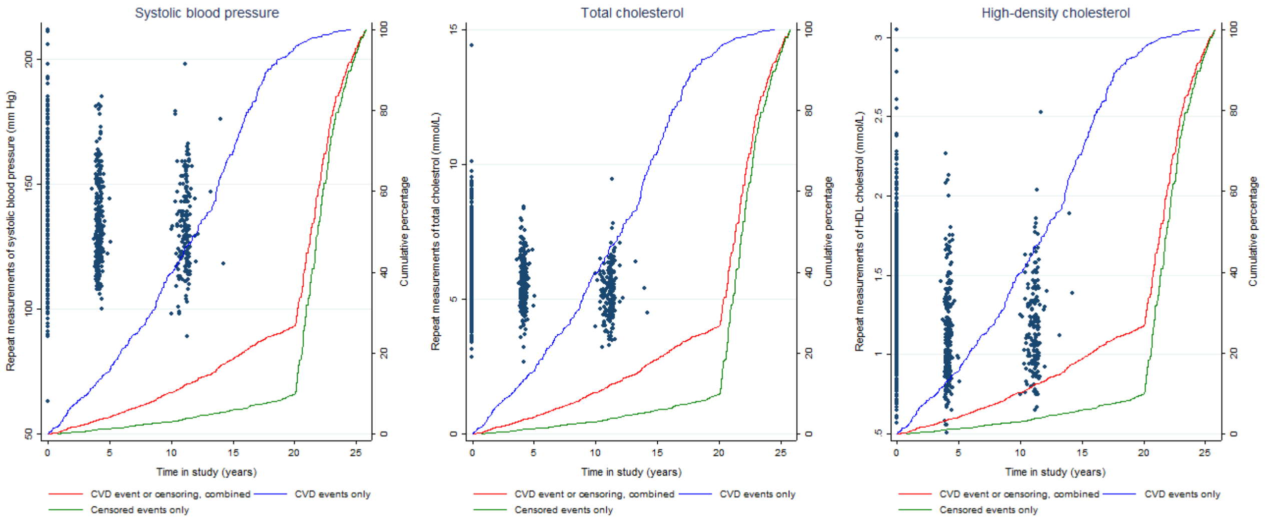

LEADER

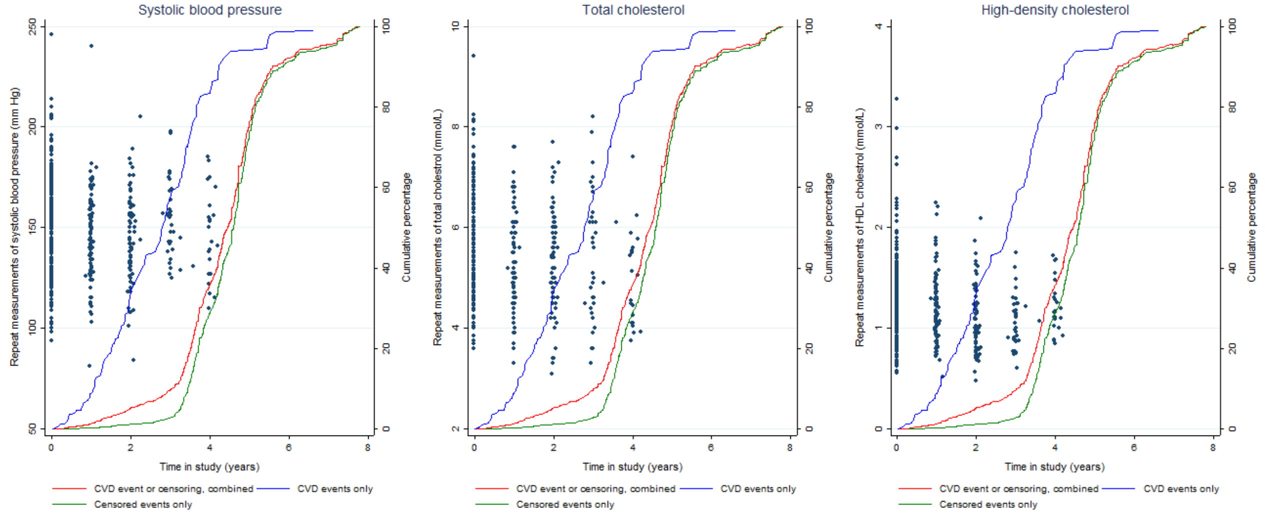

MCVDRFP

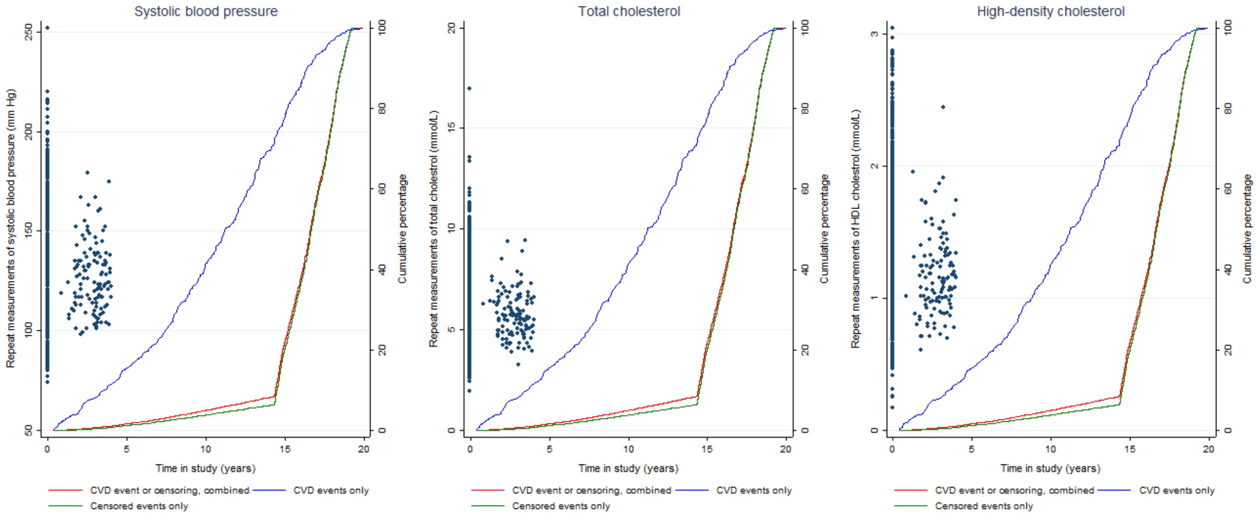

MESA

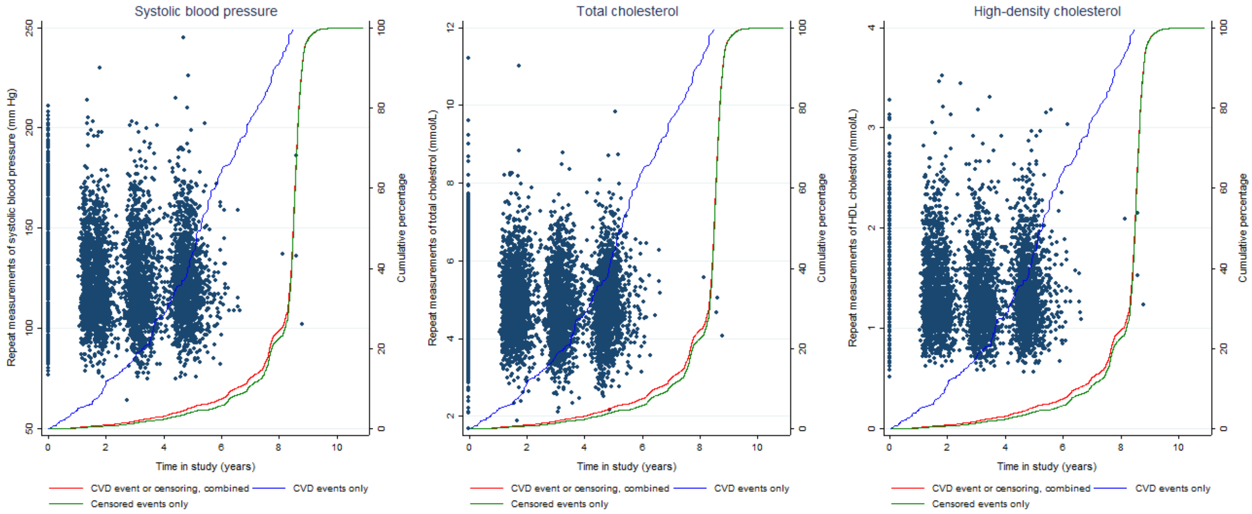

MOGERAUG3

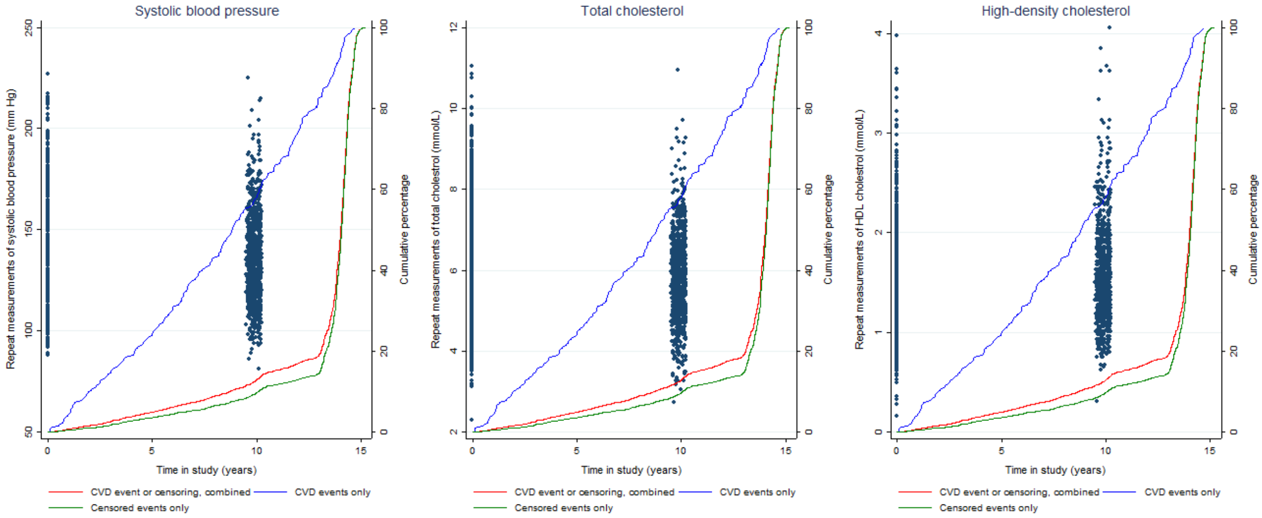

MRFIT

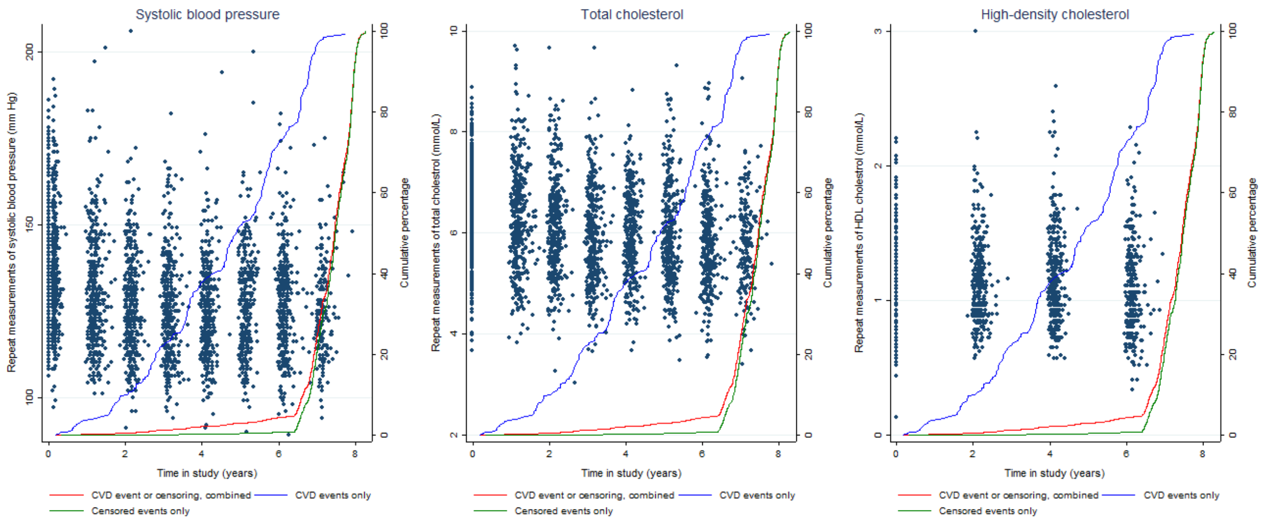

OSAKA

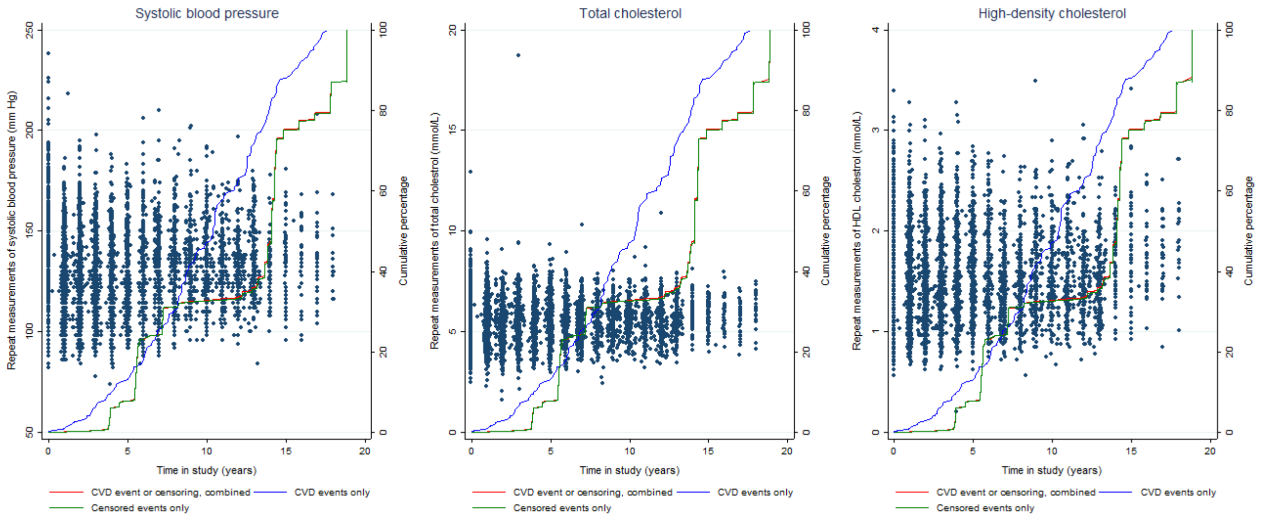

PREVEND

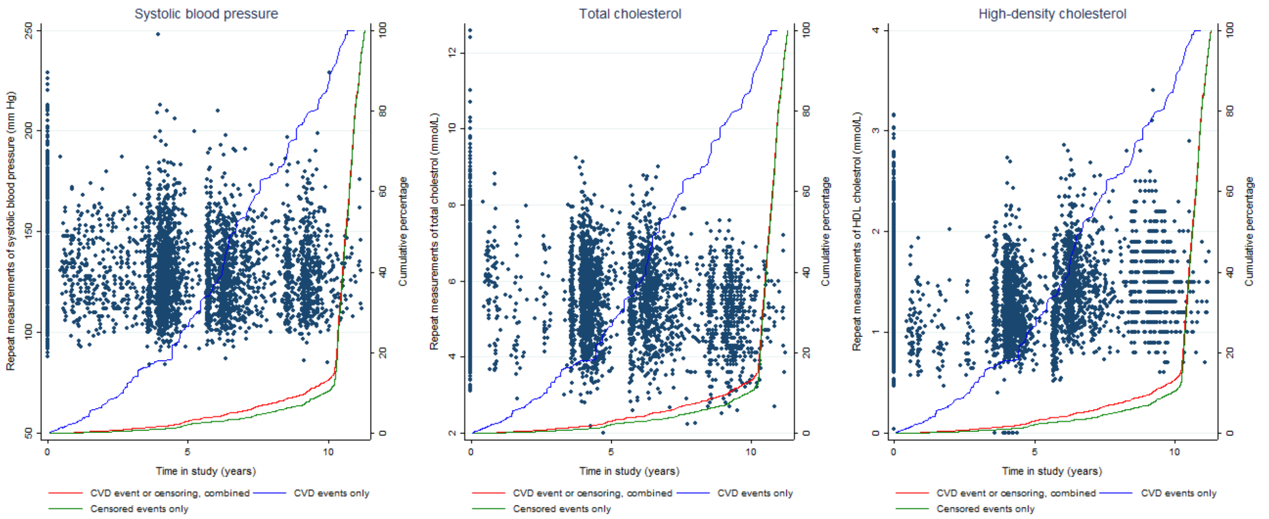

PROCAM

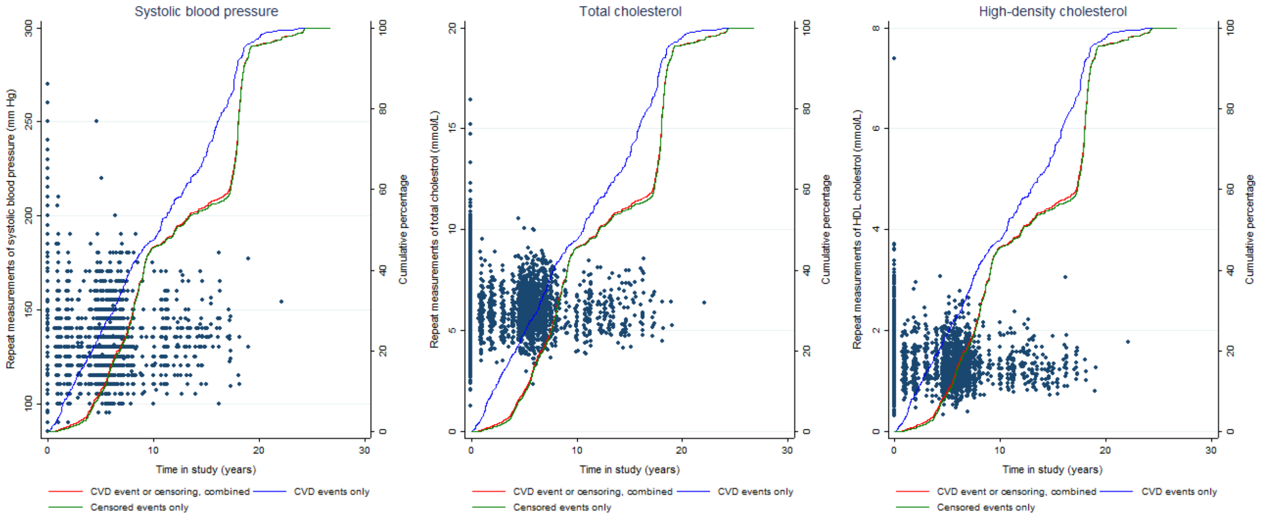

QUEBEC

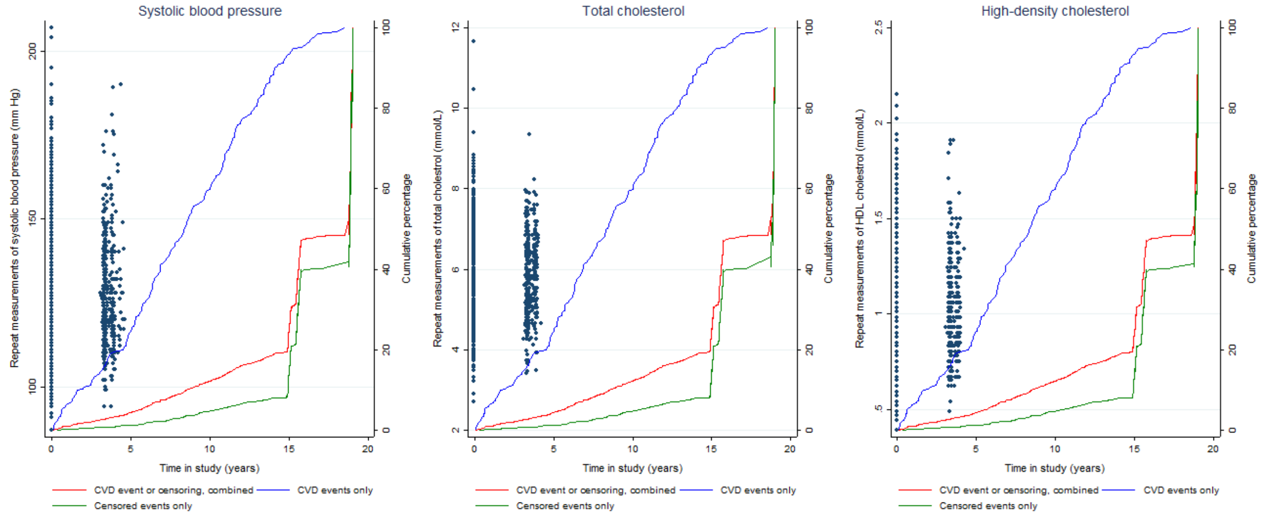

REYK

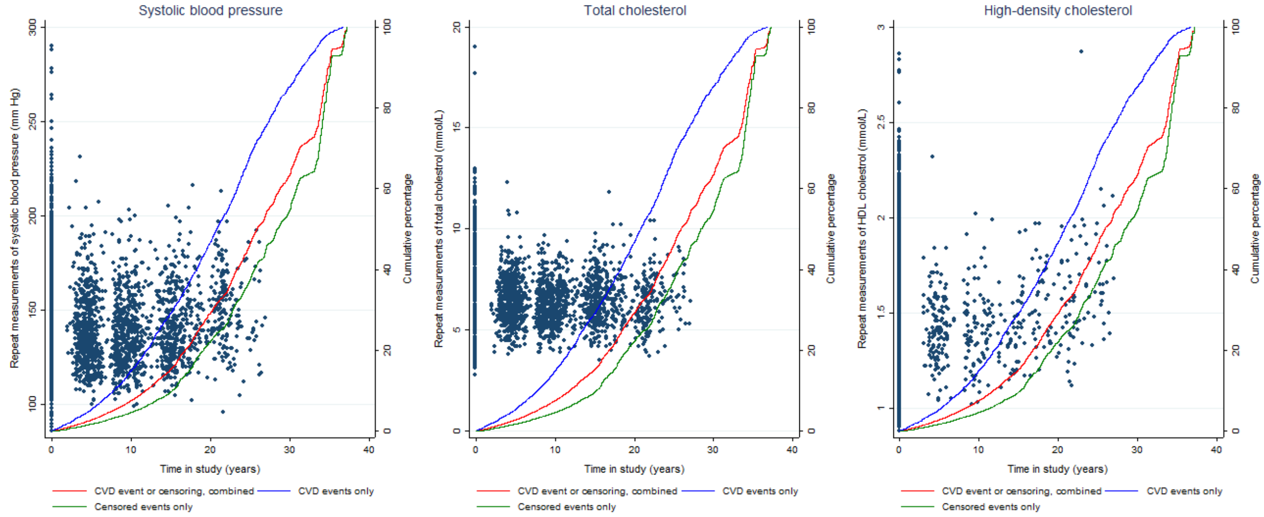

ROTT/RS-I

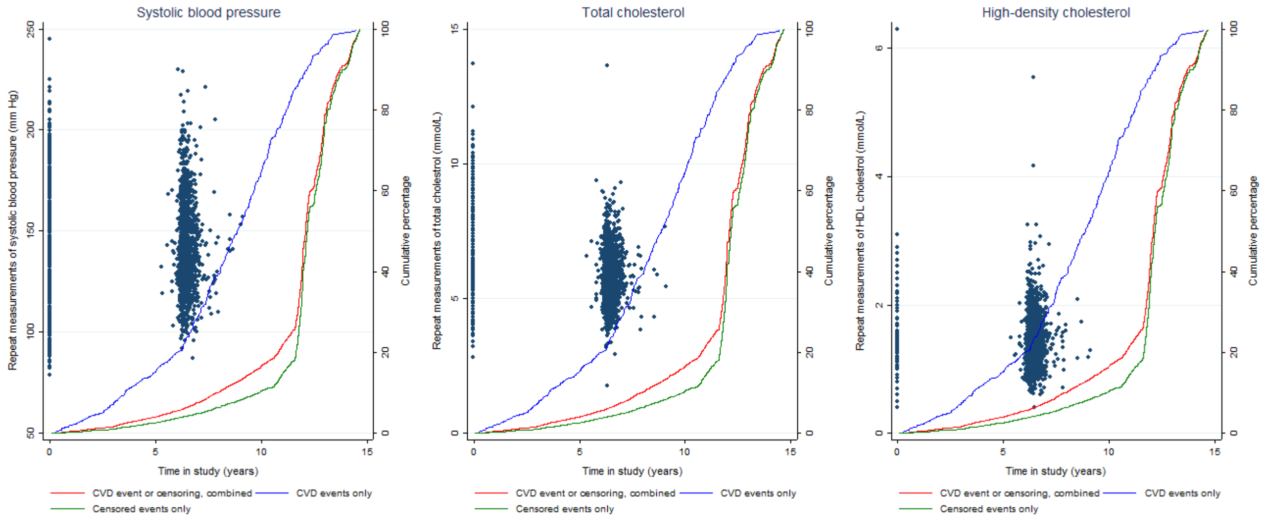

SHIP

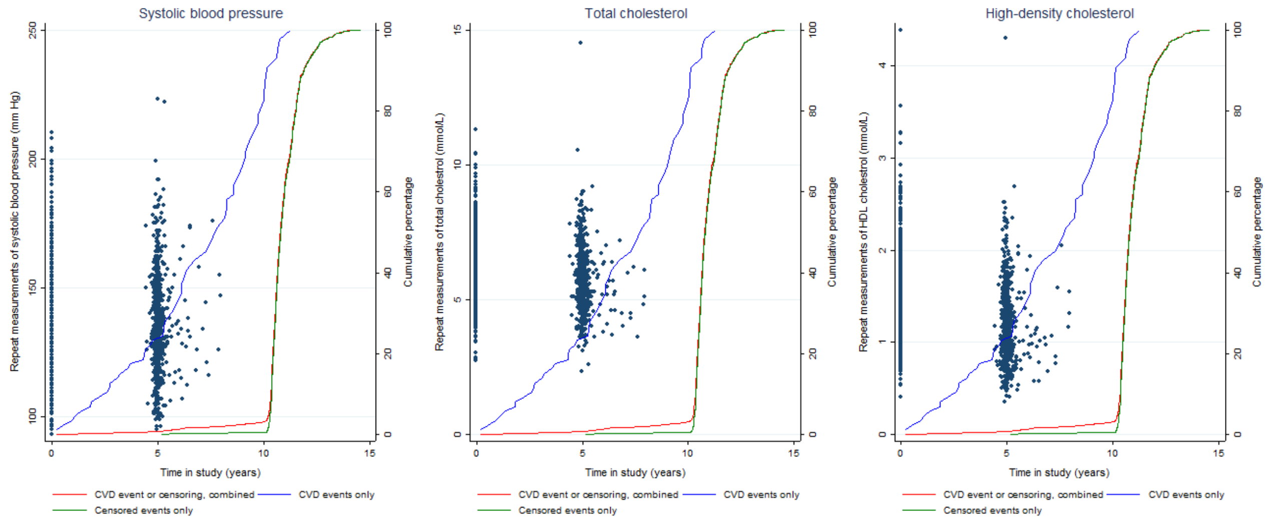

SHS

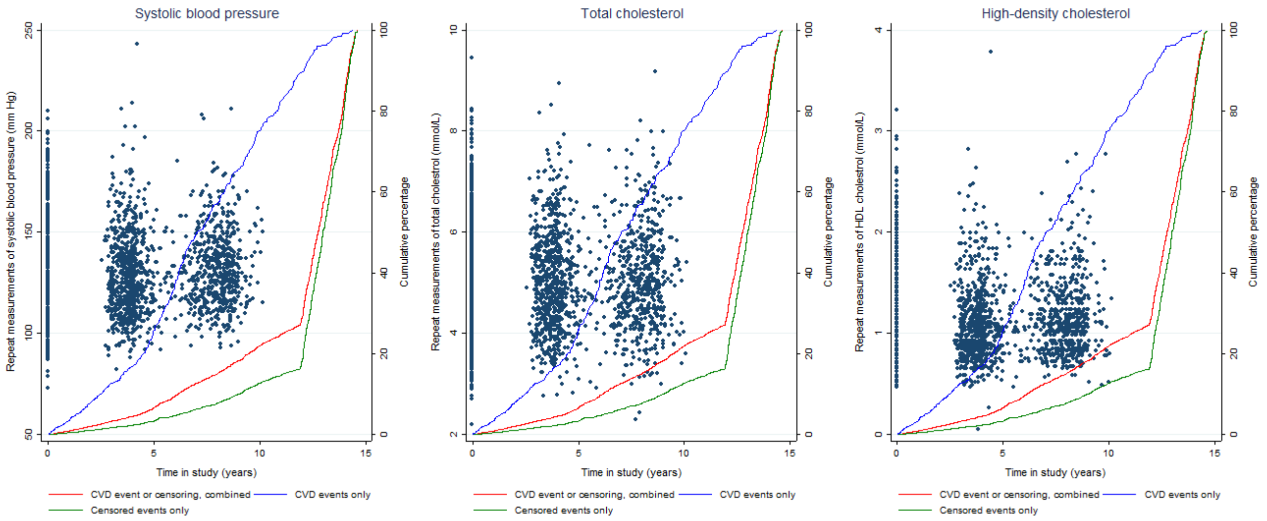

TARFS

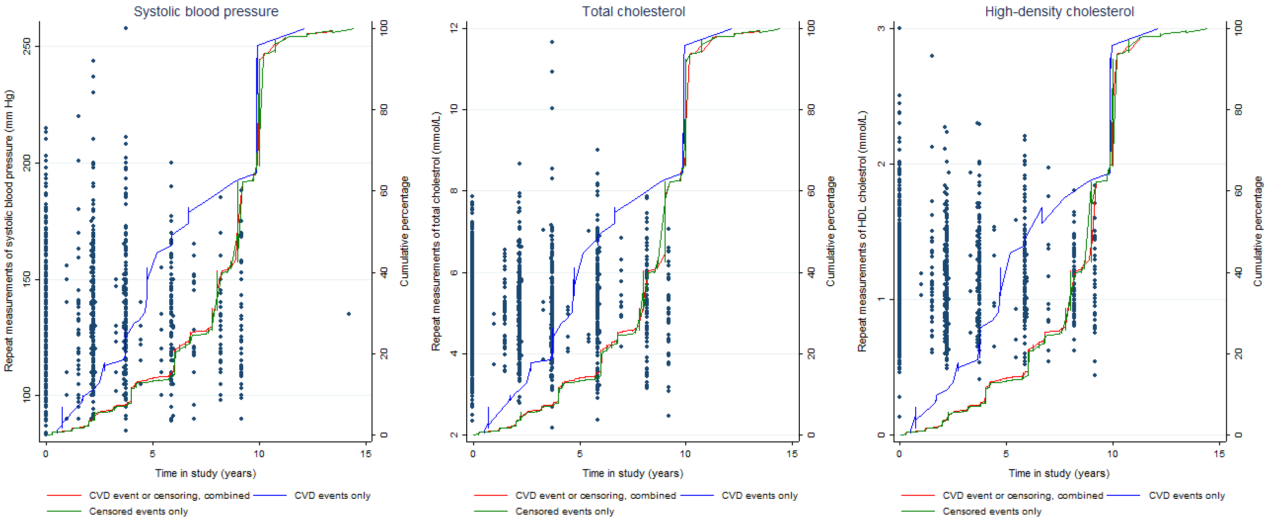

TROMSØ

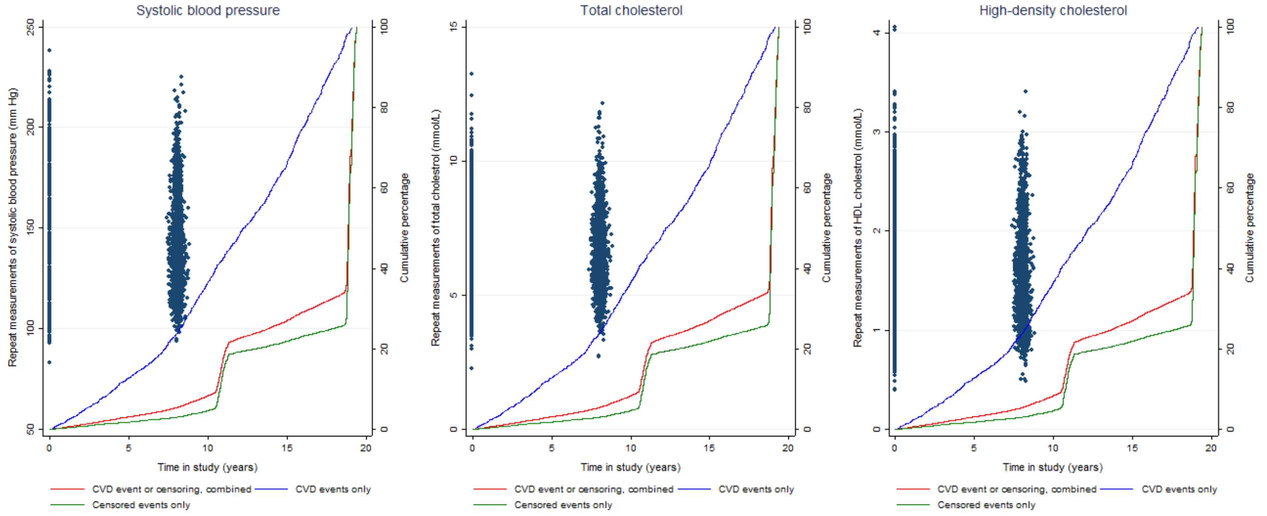

ULSAM

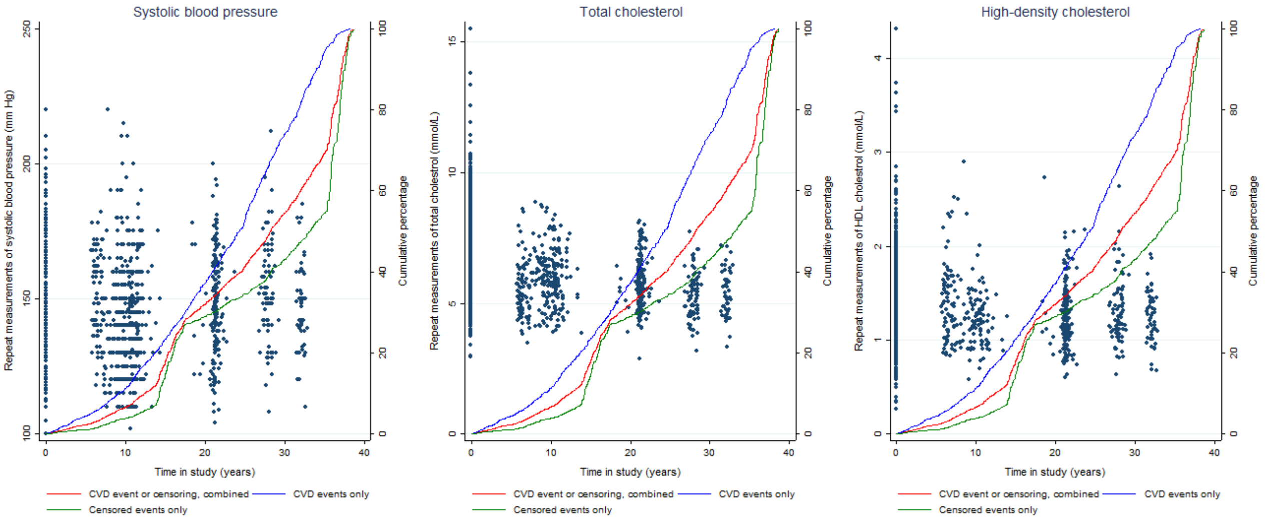

WHITEII

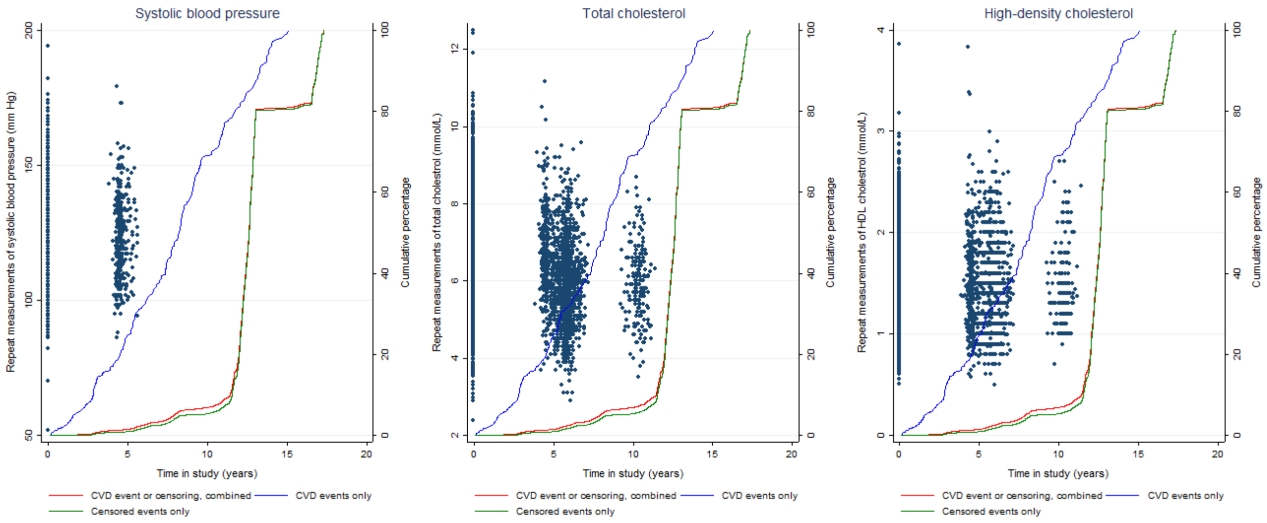

ZUTE

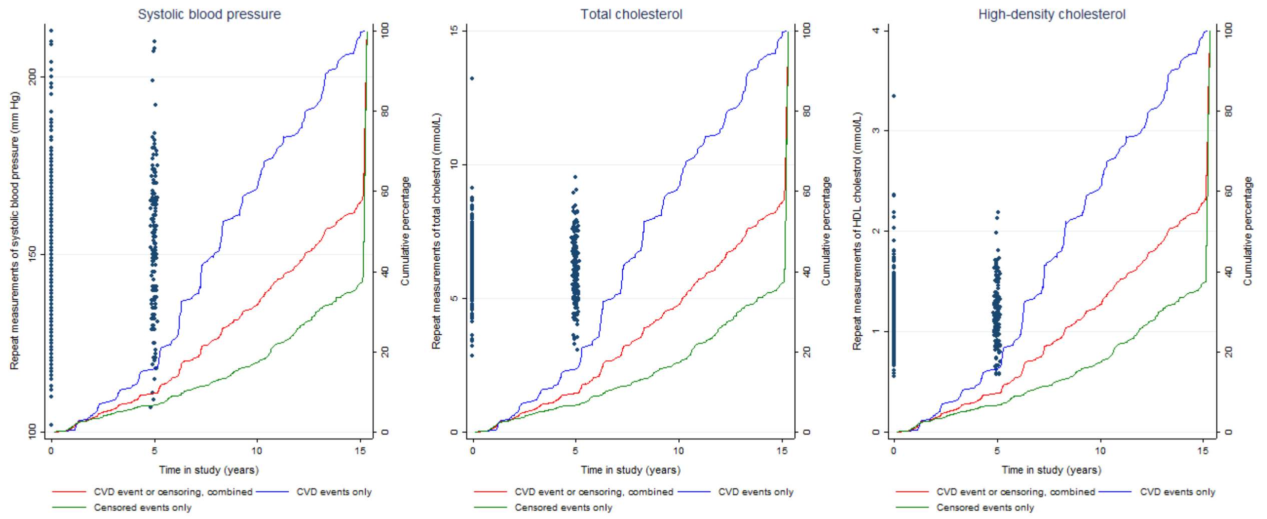

Web Figure 3. Regression dilution ratio graphs for systolic blood pressure, total cholesterol, and HDL cholesterol

Systolic blood pressure

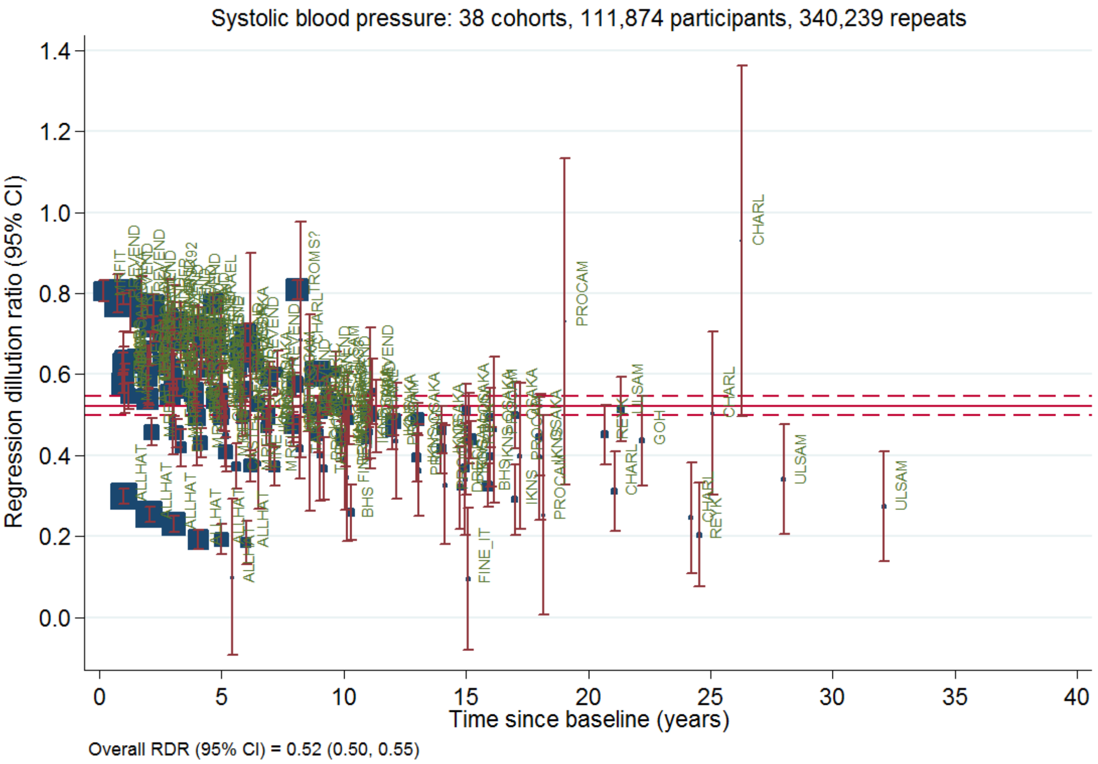

Adjusted for: sex, age, smoking status, history of diabetes, baseline total cholesterol, and baseline HDL cholesterol.

Total cholesterol

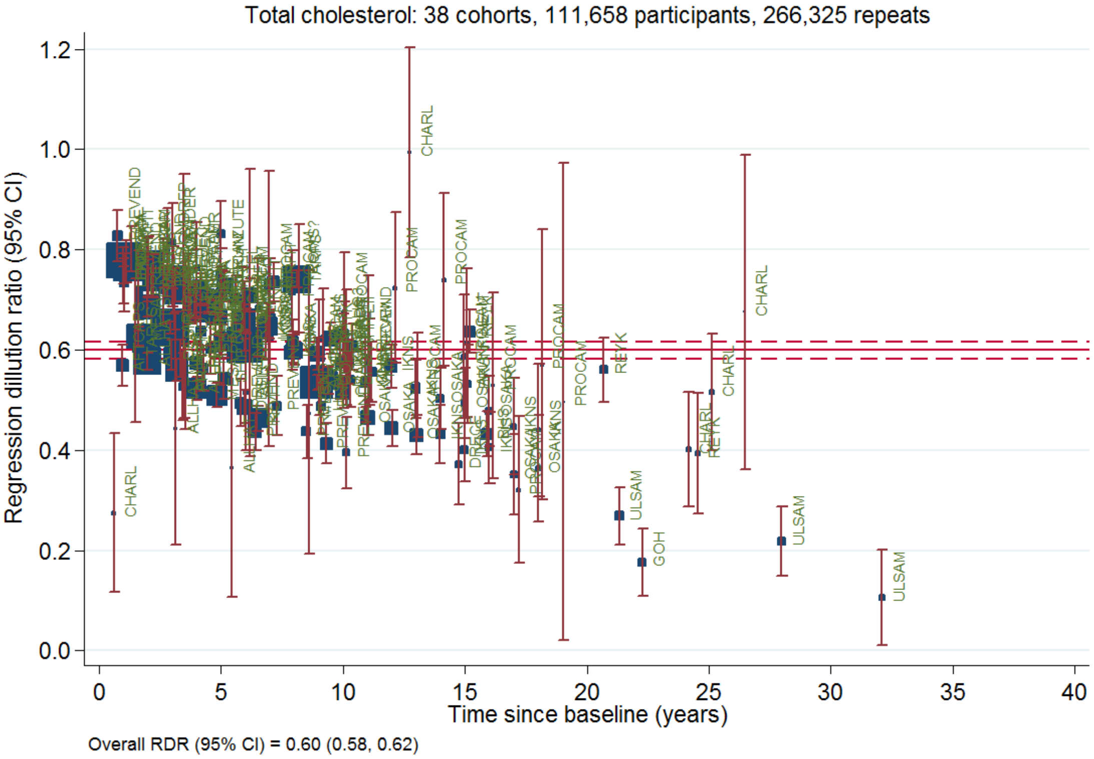

Adjusted for: sex, age, smoking status, history of diabetes, baseline systolic blood pressure, and baseline HDL cholesterol.

(Web Figure 3 continued: Regression dilution ratio graphs for systolic blood pressure, total cholesterol, and HDL cholesterol)

High-density lipoprotein (HDL) cholesterol

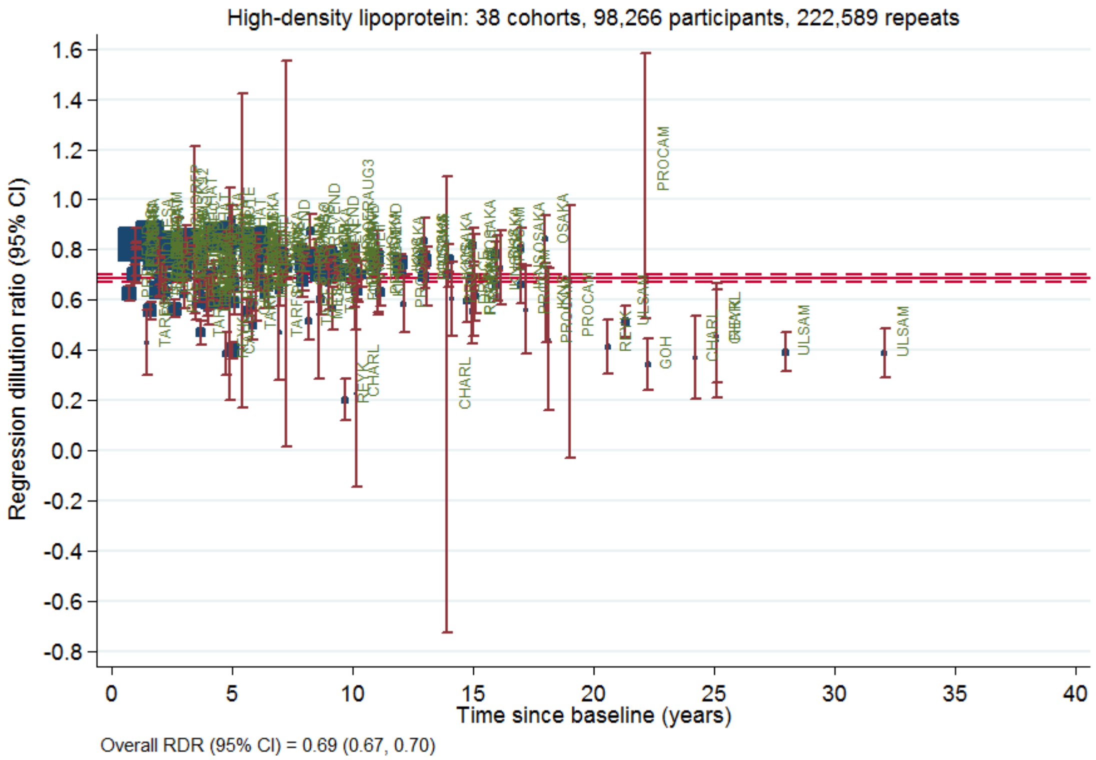

Adjusted for: sex, age, smoking status, history of diabetes, baseline systolic blood pressure, and baseline total cholesterol.

Web Table 4. Results from the longitudinal models of systolic blood pressure, total cholesterol, and HDL cholesterol

	Change in Systolic Blood Pressure, (β) (95% CI)	Change in Total Cholesterol, (β) (95% CI)	Change in HDL Cholesterol, (β) (95% CI)
**Sex**			
Male	Ref	Ref	Ref
Female	-1.63 (-2.81, -0.46)	0.21 (0.15, 0.28)	0.23 (0.20, 0.26)
**Smoking status**			
Other	Ref	Ref	Ref
Current	-1.86 (-2.02, -1.69)	0.07 (0.06, 0.08)	-0.05 (-0.05, -0.04)
**Diabetes**			
Other	Ref	Ref	Ref
Definite diabetic	4.17 (3.88, 4.46)	-0.08 (-0.10, -0.06)	-0.11 (-0.11, -0.10)
**Baseline systolic blood pressure**	N/A	0.00 (0.00, 0.00)	0.00 (0.00, 0.00)
**Baseline total cholesterol**	1.38 (1.31, 1.45)	N/A	0.02 (0.02, 0.02)
**Baseline HDL cholesterol**	-1.21 (-1.42, -1.00)	0.19 (0.18, 0.20)	N/A
**Time**	0.18 (-0.08, 0.45)	-0.03 (-0.06, -0.01)	0.00 (-0.01, 0.00)
**Random effects parameters - study**			
Random slope standard deviation	0.84 (0.66, 1.06)	0.08 (0.06, 0.10)	0.08 (0.06, 0.11)
Random sex parameter standard deviation	3.20 (2.47, 4.14)	0.18 (0.14, 0.24)	0.02 (0.01, 0.02)
Random intercept standard deviation	8.12 (6.42, 10.27)	0.59 (0.46, 0.74)	0.17 (0.13, 0.21)
**Random effects parameters - individual**			
Random slope standard deviation	1.10 (1.08, 1.12)	0.06 (0.06, 0.06)	0.02 (0.02, 0.02)
Random intercept standard deviation	14.65 (14.58, 14.72)	0.91 (0.91, 0.91)	0.30 (0.29, 0.30)
**Residual standard deviation**	11.19 (11.16, 11.23)	0.54 (0.54, 0.54)	0.19 (0.19, 0.19)

CI=confidence interval; HDL=high density lipoprotein

Web Figure 4. Distribution of absolute cardiovascular disease risk scores

Model 1: single time point model

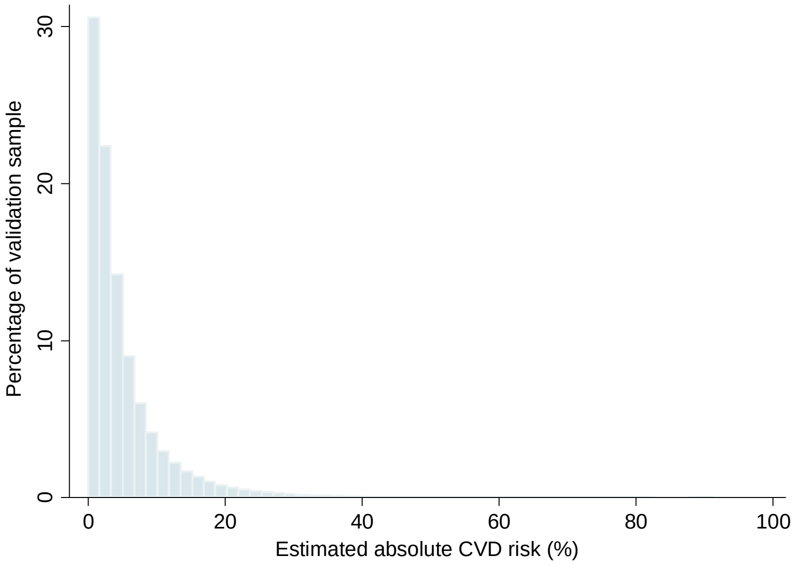

Model 2: cumulative means model

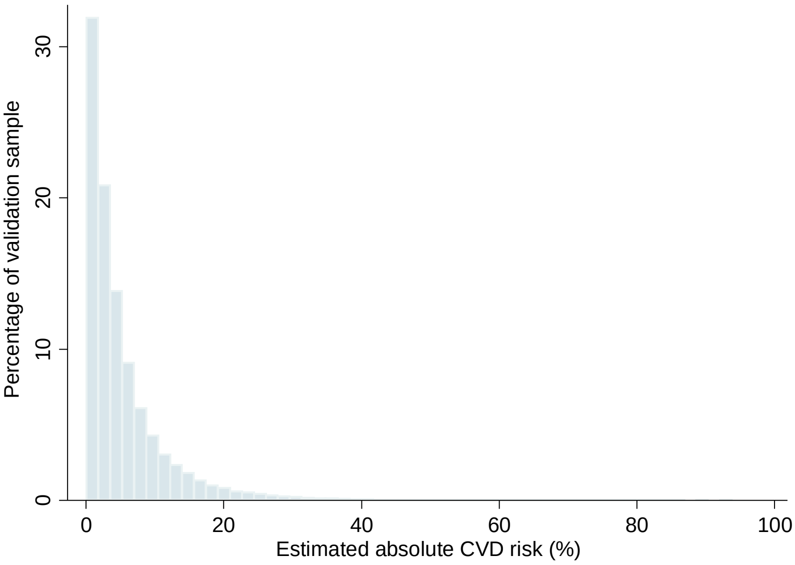

Model 3: longitudinal models

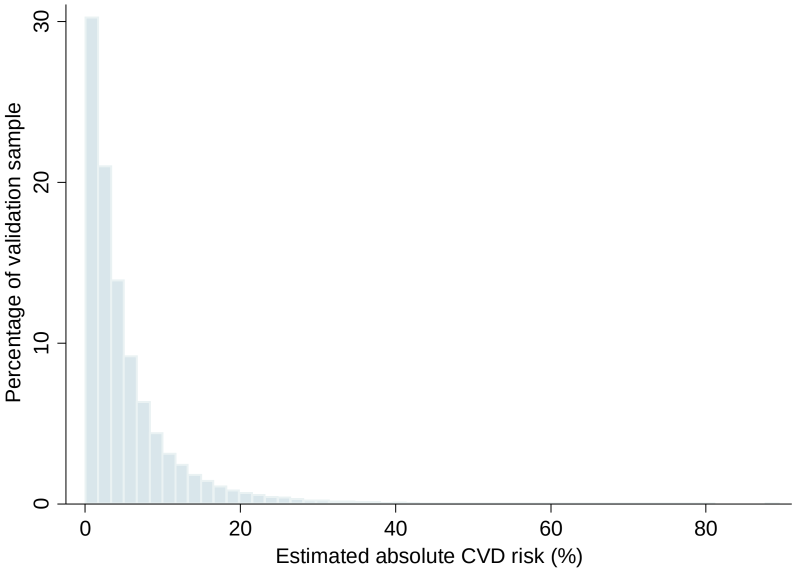

Web Figure 5. Measures of cardiovascular disease risk discrimination in each study

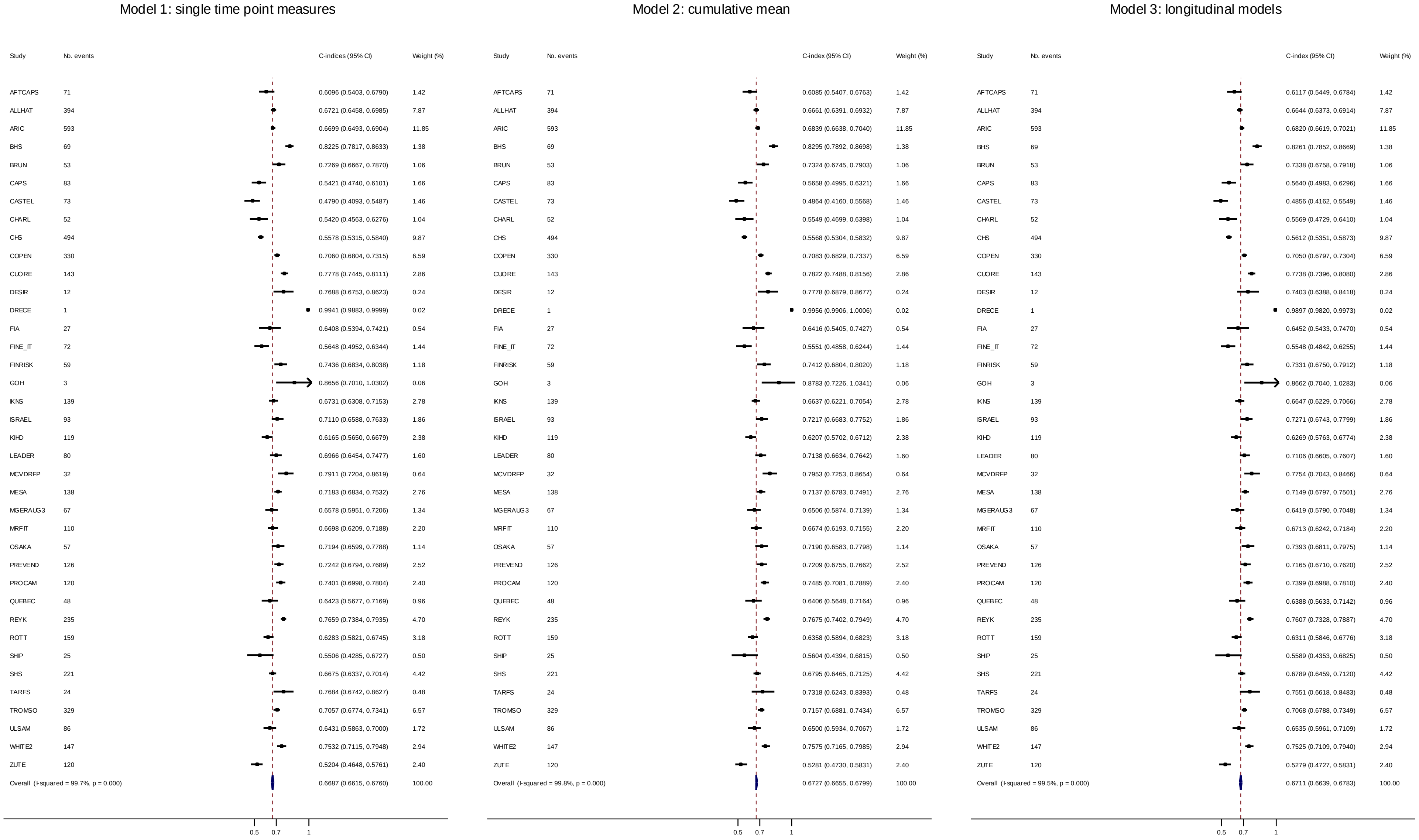

Model 1 used baseline measures of systolic blood pressure, total cholesterol and HDL cholesterol. Model 2 used cumulative means of systolic blood pressure, total cholesterol and HDL cholesterol. Model 3 used individual-level random intercepts and slopes for systolic blood pressure, total cholesterol and HDL cholesterol.

Web Figure 6. Changes in cardiovascular disease risk discrimination in each study

Model 2 compared to Model 1

Study	No. events	Change in C-index (95% CI)	Weight (%)
AFTCAPS	71	-0.0011 (-0.0148, 0.0125)	1.42
ALLHAT	394	-0.0060 (-0.0124, 0.0004)	7.87
ARIC	593	0.0140 (0.0088, 0.0193)	11.85
BHS	69	0.0070 (-0.0003, 0.0142)	1.38
BRUN	53	0.0055 (-0.0097, 0.0208)	1.06
CAPS	83	0.0237 (0.0095, 0.0379)	1.66
CASTEL	73	0.0074 (-0.0047, 0.0195)	1.46
CHARL	52	0.0129 (-0.0035, 0.0293)	1.04
CHS	494	-0.0010 (-0.0101, 0.0081)	9.87
COPEN	330	0.0023 (-0.0014, 0.0061)	6.59
CUORE	143	0.0044 (-0.0012, 0.0099)	2.86
DESIR	12	0.0090 (-0.0054, 0.0234)	0.24
DRECE	1	0.0015 (-0.0014, 0.0044)	0.02
FIA	27	0.0008 (-0.0064, 0.0080)	0.54
FINE_IT	72	-0.0097 (-0.0354, 0.0160)	1.44
FINRISK	59	-0.0024 (-0.0069, 0.0022)	1.18
GOH	3	0.0128 (0.0021, 0.0235)	0.06
IKNS	139	-0.0093 (-0.0196, 0.0009)	2.78
ISRAEL	93	0.0107 (0.0009, 0.0206)	1.86
KIHD	119	0.0042 (-0.0067, 0.0152)	2.38
LEADER	80	0.0172 (0.0020, 0.0325)	1.60
MCVDRFP	32	0.0042 (0.0008, 0.0076)	0.64
MESA	138	-0.0046 (-0.0133, 0.0041)	2.76
MGERAUG3	67	-0.0072 (-0.0160, 0.0016)	1.34
MRFIT	110	-0.0024 (-0.0174, 0.0125)	2.20
OSAKA	57	-0.0003 (-0.0137, 0.0131)	1.14
PREVEND	126	-0.0033 (-0.0089, 0.0023)	2.52
PROCAM	120	0.0084 (0.0003, 0.0165)	2.40
QUEBEC	48	-0.0017 (-0.0099, 0.0065)	0.96
REYK	235	0.0016 (-0.0014, 0.0046)	4.70
ROTT	159	0.0076 (-0.0001, 0.0152)	3.18
SHIP	25	0.0099 (-0.0092, 0.0289)	0.50
SHS	221	0.0120 (0.0052, 0.0189)	4.42
TARFS	24	-0.0367 (-0.0611, -0.0122)	0.48
TROMSO	329	0.0100 (0.0060, 0.0139)	6.57
ULSAM	86	0.0069 (-0.0043, 0.0181)	1.72
WHITE2	147	0.0043 (-0.0028, 0.0115)	2.94
ZUTE	120	0.0077 (-0.0048, 0.0201)	2.40
Overall (I-squared = 66.8%, p = 0.000)		0.0040 (0.0023, 0.0057)	100.00

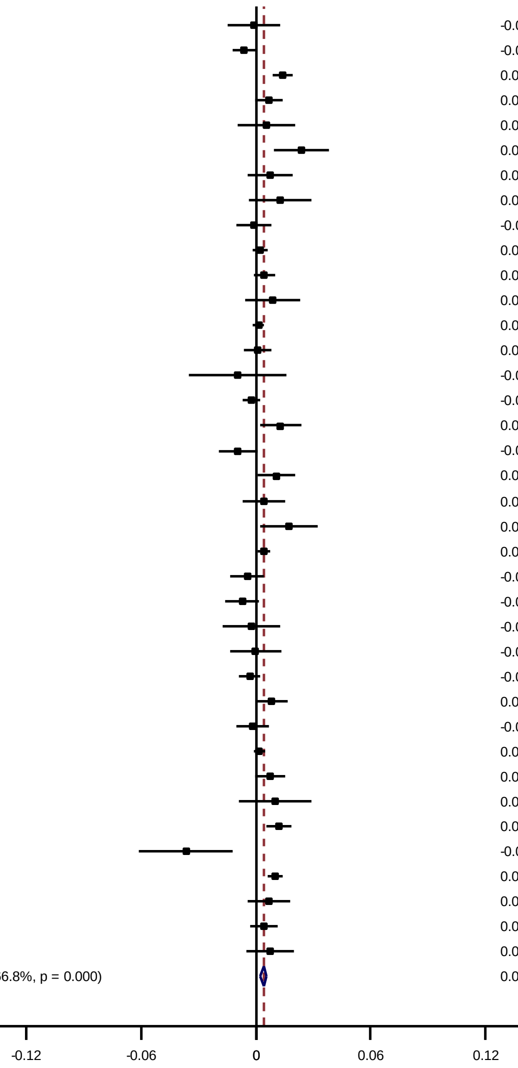

Model 3 compared to Model 1

Study	No. events	Change in C-index (95% CI)	Weight (%)
AFTCAPS	71	0.0020 (-0.0117, 0.0157)	1.42
ALLHAT	394	-0.0078 (-0.0141, -0.0014)	7.87
ARIC	593	0.0122 (0.0061, 0.0182)	11.85
BHS	69	0.0036 (-0.0052, 0.0123)	1.38
BRUN	53	0.0069 (-0.0118, 0.0257)	1.06
CAPS	83	0.0219 (0.0089, 0.0348)	1.66
CASTEL	73	0.0065 (-0.0069, 0.0200)	1.46
CHARL	52	0.0150 (-0.0047, 0.0346)	1.04
CHS	494	0.0034 (-0.0068, 0.0136)	9.87
COPEN	330	-0.0009 (-0.0058, 0.0039)	6.59
CUORE	143	-0.0041 (-0.0108, 0.0027)	2.86
DESIR	12	-0.0285 (-0.0464, -0.0106)	0.24
DRECE	1	-0.0044 (-0.0109, 0.0020)	0.02
FIA	27	0.0044 (-0.0053, 0.0141)	0.54
FINE_IT	72	-0.0099 (-0.0396, 0.0197)	1.44
FINRISK	59	-0.0105 (-0.0185, -0.0025)	1.18
GOH	3	0.0006 (-0.0035, 0.0047)	0.06
IKNS	139	-0.0083 (-0.0213, 0.0047)	2.78
ISRAEL	93	0.0161 (0.0061, 0.0260)	1.86
KIHD	119	0.0104 (-0.0024, 0.0232)	2.38
LEADER	80	0.0140 (-0.0004, 0.0285)	1.60
MCVDRFP	32	-0.0157 (-0.0265, -0.0049)	0.64
MESA	138	-0.0034 (-0.0122, 0.0054)	2.76
MGERAUG3	67	-0.0160 (-0.0269, -0.0050)	1.34
MRFIT	110	0.0014 (-0.0142, 0.0171)	2.20
OSAKA	57	0.0199 (0.0046, 0.0352)	1.14
PREVEND	126	-0.0077 (-0.0147, -0.0007)	2.52
PROCAM	120	-0.0002 (-0.0076, 0.0072)	2.40
QUEBEC	48	-0.0036 (-0.0110, 0.0039)	0.96
REYK	235	-0.0052 (-0.0090, -0.0014)	4.70
ROTT	159	0.0028 (-0.0057, 0.0113)	3.18
SHIP	25	0.0083 (-0.0192, 0.0359)	0.50
SHS	221	0.0114 (0.0039, 0.0189)	4.42
TARFS	24	-0.0133 (-0.0384, 0.0117)	0.48
TROMSO	329	0.0011 (-0.0032, 0.0054)	6.57
ULSAM	86	0.0104 (-0.0045, 0.0252)	1.72
WHITE2	147	-0.0007 (-0.0096, 0.0082)	2.94
ZUTE	120	0.0075 (-0.0051, 0.0200)	2.40
Overall (I-squared = 73.7%, p = 0.000)		0.0023 (0.0005, 0.0042)	100.00

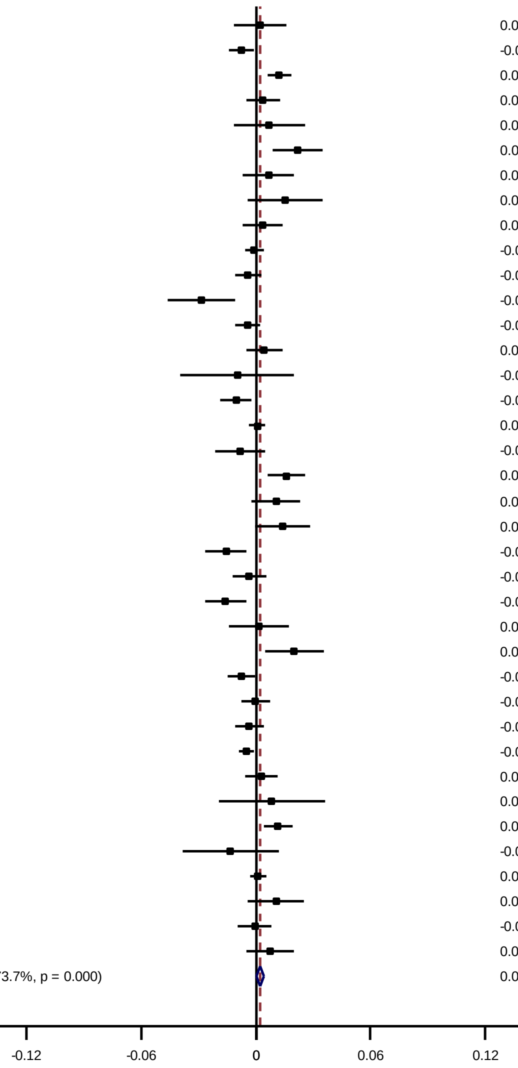

Model 1 used baseline measures of systolic blood pressure, total cholesterol and HDL cholesterol. Model 2 used cumulative means of systolic blood pressure, total cholesterol and HDL cholesterol. Model 3 used individual-level random intercepts and slopes for systolic blood pressure, total cholesterol and HDL cholesterol.

Web Table 5. Changes in risk discrimination difference according to mean age, number of repeats and follow-up time in a study

	Change in C-index difference between Model 2 and Model 1 (95% CI)	Change in C-index difference between Model 3 and Model 1 (95% CI)
**Mean age (years)**	**-0.0004 (-0.0007, -0.0000)**)	-0.0001 (-0.0005, 0.0003)
**Mean number of repeats**		
Systolic blood pressure	-0.0014 (-0.0031, 0.0004)	0.0009 (-0.0010, 0.0028)
Total cholesterol	-0.0002 (-0.0032, 0.0027)	0.0027 (-0.0003, 0.0056)
HDL cholesterol	0.0011 (-0.0019, 0.0040)	**0.0032 (0.0003, 0.0061)**
**Follow-up time (years)**		
Maximum	0.0001 (-0.0003, 0.0006)	0.0001 (-0.0003, 0.0006)

Results are weighted by the number of CVD events in each study. Interpretation: e.g. for every one unit increase in the mean age of participants in a study, the difference in risk discrimination between Model 2 and Model 1 decreased by 0.0004 (-0.0007, 0.0000). Statistically significant results in bold.

Web Table 6. Reclassification of events and non-events in the validation data of the models

	Model 1		Model 2		Model 3	
**Risk category**	**Non-event no. (%)**	**Event no. (%)**	**Non-event no. (%)**	**Event no. (%)**	**Non-event no. (%)**	**Event no. (%)**
0-<2.5%	31,120 (53)	597 (12)	31,865 (54)	564 (11)	30,986 (52)	549 (11)
2.5-<3.75%	7,939 (13)	529 (11)	7,730 (13)	516 (10)	7,978 (13)	515 (10)
≥3.75%	20,063 (34)	3,878 (78)	19,527 (33)	3,924 (78)	20,158 (34)	3,940 (79)

Total number events=5,004 and total number non-events=59,122.

Web Figure 7. Changes in cardiovascular disease risk discrimination in each study, using study-specific survival models

Model 2 compared to Model 1

Study	No. events	Change in C-index (95% CI)	Weight (%)
AFTCAPS	71	0.0073 (-0.0084, 0.0230)	1.44
ALLHAT	394	-0.0069 (-0.0135, -0.0003)	7.97
ARIC	593	0.0169 (0.0110, 0.0228)	12.00
BHS	69	0.0043 (-0.0016, 0.0101)	1.40
BRUN	53	0.0078 (-0.0076, 0.0232)	1.07
CAPS	83	0.0219 (0.0070, 0.0368)	1.68
CASTEL	73	-0.0015 (-0.0058, 0.0027)	1.48
CHARL	52	0.0276 (0.0079, 0.0472)	1.05
CHS	494	0.0035 (-0.0053, 0.0124)	10.00
COPEN	330	0.0024 (-0.0012, 0.0060)	6.68
CUORE	143	0.0036 (-0.0004, 0.0076)	2.89
DESIR	12	0.0021 (-0.0146, 0.0189)	0.24
DRECE	1	-0.0015 (-0.0065, 0.0035)	0.02
FIA	27	0.0010 (-0.0066, 0.0085)	0.55
FINE_IT	72	-0.0188 (-0.0445, 0.0070)	1.46
IKNS	139	-0.0013 (-0.0103, 0.0076)	2.81
ISRAEL	93	0.0126 (0.0002, 0.0250)	1.88
KIHD	119	0.0117 (-0.0013, 0.0246)	2.41
LEADER	80	0.0276 (0.0033, 0.0518)	1.62
MCVDRFP	32	0.0002 (-0.0006, 0.0010)	0.65
MESA	138	-0.0079 (-0.0234, 0.0077)	2.79
MGERAUG3	67	-0.0040 (-0.0111, 0.0031)	1.36
MRFIT	110	0.0012 (-0.0224, 0.0248)	2.23
OSAKA	57	0.0080 (-0.0049, 0.0210)	1.15
PREVEND	126	-0.0004 (-0.0076, 0.0068)	2.55
PROCAM	120	0.0123 (-0.0003, 0.0248)	2.43
QUEBEC	48	-0.0004 (-0.0101, 0.0094)	0.97
REYK	235	0.0019 (-0.0005, 0.0042)	4.76
ROTT	159	0.0090 (0.0033, 0.0146)	3.22
SHIP	25	0.0110 (-0.0080, 0.0299)	0.51
SHS	221	0.0093 (0.0033, 0.0153)	4.47
TARFS	24	-0.0213 (-0.0617, 0.0192)	0.49
TROMSO	329	0.0141 (0.0090, 0.0191)	6.66
ULSAM	86	0.0167 (0.0018, 0.0316)	1.74
WHITE2	147	-0.0009 (-0.0104, 0.0086)	2.97
ZUTE	120	0.0068 (-0.0070, 0.0207)	2.43
Overall (I-squared = 89.1%, p = 0.000)		0.0059 (0.0040, 0.0078)	100.00

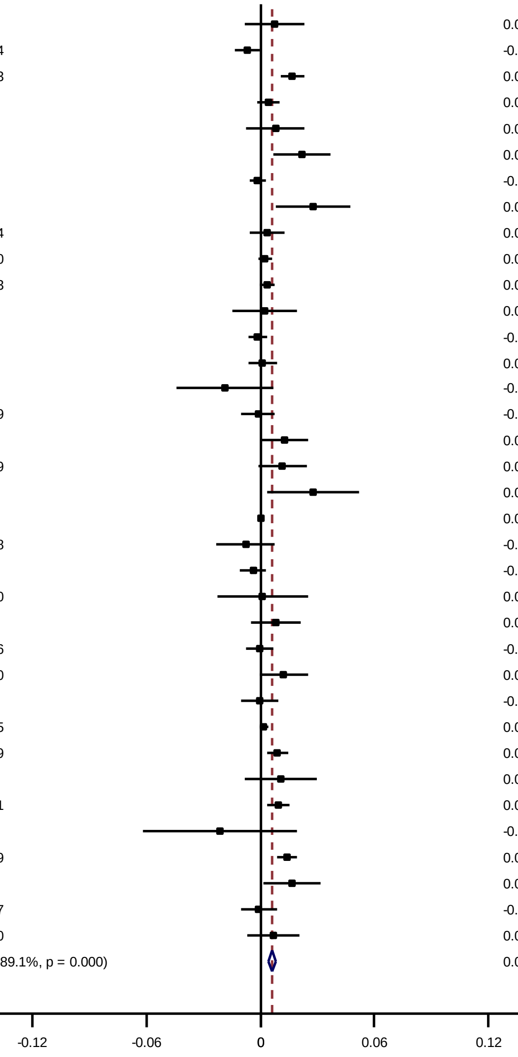

Model 3 compared to Model 1

Study	No. events	Change in C-index (95% CI)	Weight (%)
AFTCAPS	71	0.0005 (-0.0240, 0.0249)	1.44
ALLHAT	394	-0.0151 (-0.0253, -0.0049)	7.97
ARIC	593	0.0144 (0.0077, 0.0211)	12.00
BHS	69	0.0031 (-0.0048, 0.0110)	1.40
BRUN	53	-0.0013 (-0.0369, 0.0343)	1.07
CAPS	83	0.0158 (-0.0017, 0.0333)	1.68
CASTEL	73	0.0033 (-0.0118, 0.0185)	1.48
CHARL	52	0.0278 (0.0107, 0.0449)	1.05
CHS	494	0.0061 (-0.0037, 0.0160)	10.00
COPEN	330	0.0005 (-0.0054, 0.0063)	6.68
CUORE	143	-0.0010 (-0.0071, 0.0052)	2.89
DESIR	12	-0.0585 (-0.0882, -0.0287)	0.24
DRECE	1	-0.0015 (-0.0119, 0.0090)	0.02
FIA	27	-0.0040 (-0.0152, 0.0072)	0.55
FINE_IT	72	-0.0279 (-0.0552, -0.0007)	1.46
IKNS	139	0.0017 (-0.0108, 0.0141)	2.81
ISRAEL	93	0.0140 (0.0008, 0.0272)	1.88
KIHD	119	0.0342 (0.0171, 0.0513)	2.41
LEADER	80	0.0300 (-0.0028, 0.0629)	1.62
MCVDRFP	32	-0.0232 (-0.0401, -0.0064)	0.65
MESA	138	-0.0144 (-0.0305, 0.0016)	2.79
MGERAUG3	67	-0.0128 (-0.0230, -0.0026)	1.36
MRFIT	110	-0.0195 (-0.0551, 0.0161)	2.23
OSAKA	57	0.0163 (-0.0082, 0.0407)	1.15
PREVEND	126	-0.0148 (-0.0286, -0.0010)	2.55
PROCAM	120	-0.0001 (-0.0116, 0.0115)	2.43
QUEBEC	48	-0.0190 (-0.0343, -0.0037)	0.97
REYK	235	-0.0051 (-0.0085, -0.0017)	4.76
ROTT	159	0.0019 (-0.0069, 0.0107)	3.22
SHIP	25	0.0384 (0.0023, 0.0745)	0.51
SHS	221	0.0068 (0.0007, 0.0128)	4.47
TARFS	24	-0.0077 (-0.0481, 0.0326)	0.49
TROMSO	329	0.0044 (-0.0015, 0.0103)	6.66
ULSAM	86	0.0151 (0.0015, 0.0287)	1.74
WHITE2	147	-0.0079 (-0.0227, 0.0070)	2.97
ZUTE	120	0.0068 (-0.0209, 0.0345)	2.43
Overall (I-squared = 75.8%, p = 0.000)		0.0021 (-0.0004, 0.0045)	100.00

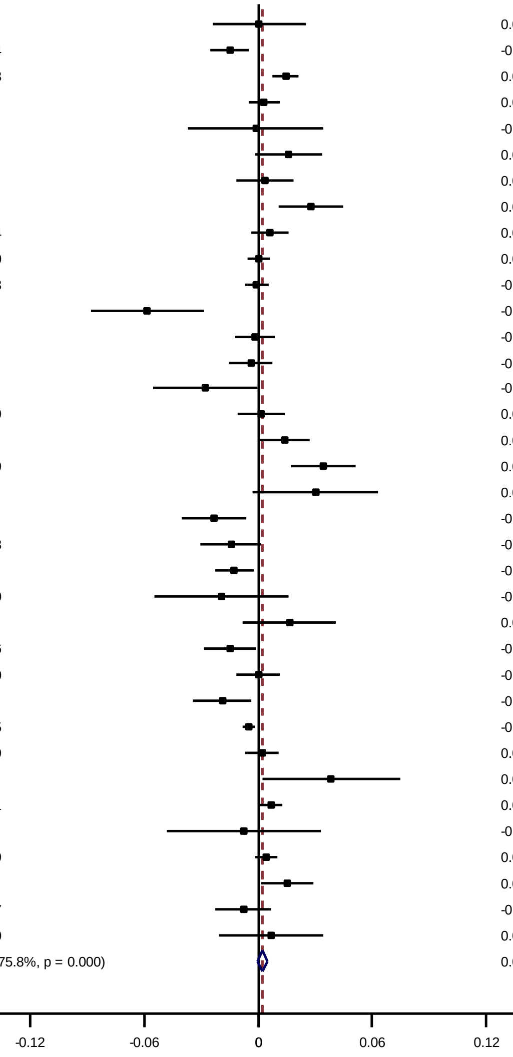

Model 1 used baseline measures of systolic blood pressure, total cholesterol and HDL cholesterol. Model 2 used cumulative means of systolic blood pressure, total cholesterol and HDL cholesterol. Model 3 used individual-level random intercepts and slopes for systolic blood pressure, total cholesterol and HDL cholesterol.

Web Figure 8. Changes in cardiovascular disease risk discrimination in each study, restricted to participants with two or more repeat measures of systolic blood pressure

Model 2 compared to Model 1

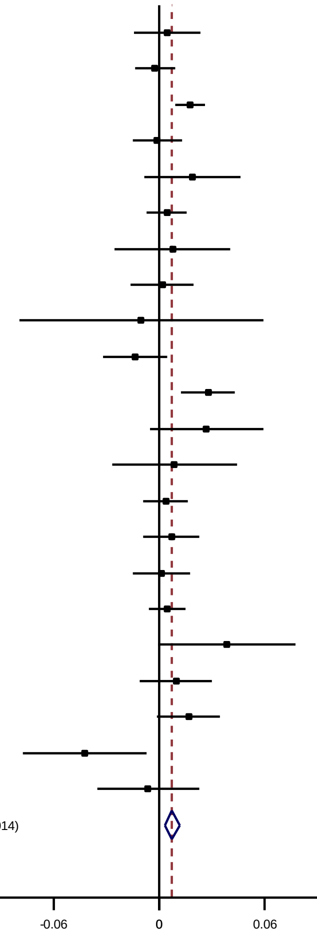

Study	No. events	Change in C-index (95% CI)	Weight (%)
AFTCAPS	53	0.0045 (-0.0146, 0.0235)	3.11
ALLHAT	206	-0.0024 (-0.0137, 0.0089)	12.09
ARIC	354	0.0174 (0.0090, 0.0259)	20.77
BHS	21	-0.0012 (-0.0154, 0.0129)	1.23
BRUN	30	0.0189 (-0.0086, 0.0463)	1.76
CHS	410	0.0042 (-0.0074, 0.0158)	24.06
CUORE	21	0.0075 (-0.0253, 0.0403)	1.23
DESIR	3	0.0016 (-0.0162, 0.0193)	0.18
FINE_IT	19	-0.0103 (-0.0800, 0.0594)	1.12
IKNS	81	-0.0136 (-0.0320, 0.0047)	4.75
ISRAEL	57	0.0277 (0.0124, 0.0431)	3.35
KIHD	33	0.0267 (-0.0056, 0.0591)	1.94
LEADER	26	0.0086 (-0.0268, 0.0441)	1.53
MESA	77	0.0036 (-0.0092, 0.0165)	4.52
MRFIT	100	0.0068 (-0.0092, 0.0228)	5.87
OSAKA	40	0.0012 (-0.0155, 0.0178)	2.35
PREVEND	46	0.0046 (-0.0060, 0.0152)	2.70
PROCAM	11	0.0386 (-0.0007, 0.0780)	0.65
REYK	50	0.0096 (-0.0109, 0.0302)	2.93
SHS	52	0.0167 (-0.0012, 0.0346)	3.05
TARFS	9	-0.0424 (-0.0778, -0.0071)	0.53
ULSAM	5	-0.0065 (-0.0356, 0.0225)	0.29
Overall (I-squared = 44.3%, p = 0.014)		0.0072 (0.0030, 0.0115)	100.00

Model 3 compared to Model 1

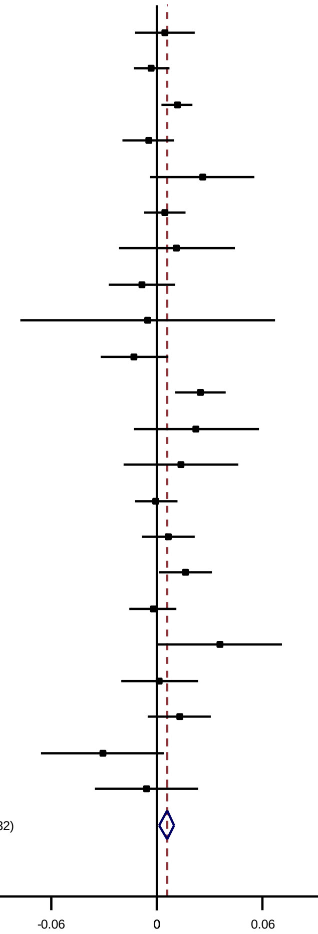

Study	No. events	Change in C-index (95% CI)	Weight (%)
AFTCAPS	53	0.0049 (-0.0121, 0.0220)	3.11
ALLHAT	206	-0.0029 (-0.0132, 0.0075)	12.09
ARIC	354	0.0116 (0.0028, 0.0203)	20.77
BHS	21	-0.0048 (-0.0197, 0.0101)	1.23
BRUN	30	0.0260 (-0.0038, 0.0558)	1.76
CHS	410	0.0047 (-0.0068, 0.0163)	24.06
CUORE	21	0.0115 (-0.0216, 0.0446)	1.23
DESIR	3	-0.0084 (-0.0273, 0.0105)	0.18
FINE_IT	19	-0.0052 (-0.0779, 0.0676)	1.12
IKNS	81	-0.0127 (-0.0322, 0.0067)	4.75
ISRAEL	57	0.0249 (0.0106, 0.0391)	3.35
KIHD	33	0.0226 (-0.0131, 0.0584)	1.94
LEADER	26	0.0138 (-0.0189, 0.0466)	1.53
MESA	77	-0.0003 (-0.0126, 0.0120)	4.52
MRFIT	100	0.0066 (-0.0086, 0.0218)	5.87
OSAKA	40	0.0164 (0.0014, 0.0313)	2.35
PREVEND	46	-0.0021 (-0.0156, 0.0114)	2.70
PROCAM	11	0.0359 (0.0002, 0.0715)	0.65
REYK	50	0.0017 (-0.0205, 0.0238)	2.93
SHS	52	0.0131 (-0.0049, 0.0311)	3.05
TARFS	9	-0.0310 (-0.0659, 0.0039)	0.53
ULSAM	5	-0.0060 (-0.0356, 0.0236)	0.29
Overall (I-squared = 39.1%, p = 0.032)		0.0059 (0.0016, 0.0101)	100.00

Model 1 used baseline measures of systolic blood pressure, total cholesterol and HDL cholesterol. Model 2 used cumulative means of systolic blood pressure, total cholesterol and HDL cholesterol. Model 3 used individual-level random intercepts and slopes for systolic blood pressure, total cholesterol and HDL cholesterol. No difference in C-index between Model 2 and Model 1, and Model 3 and Model 1, could be calculated for the studies "CHARL" and "CAPS" (all participants in the validation sample were censored) and as such, these studies were excluded from the meta-analysis of change in C-index.

Web Figure 9A. Changes in cardiovascular disease risk discrimination in each study in participants aged 40-69 years

Model 2 compared to Model 1

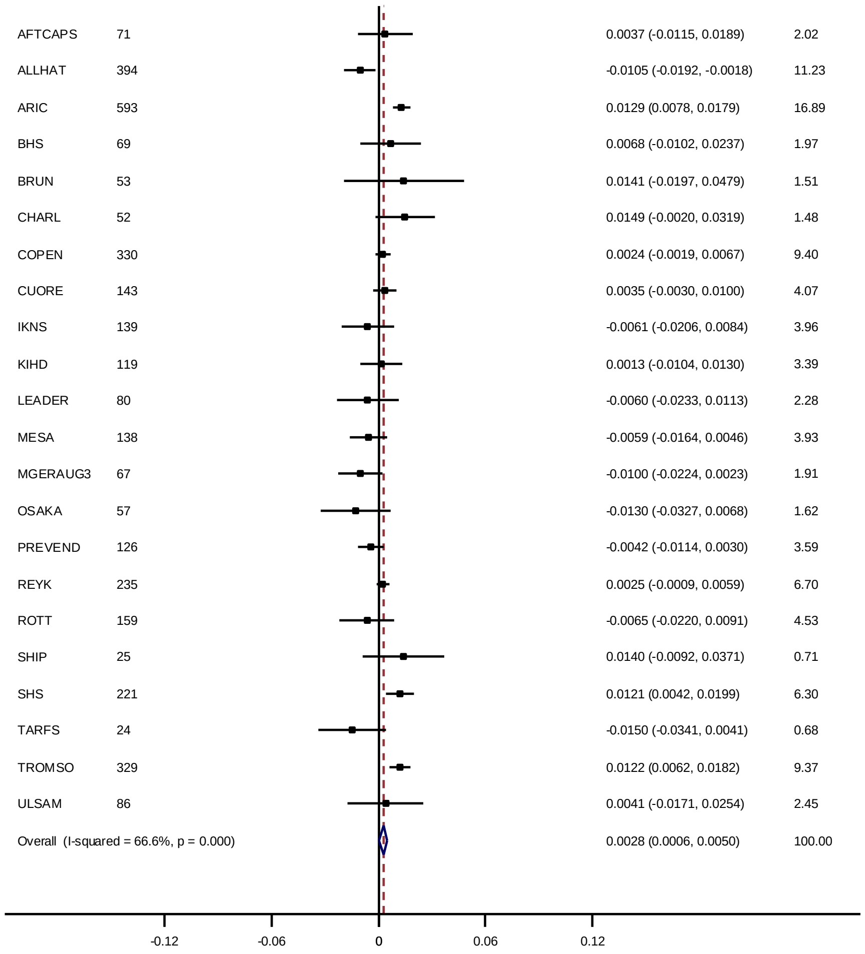

Study	No. events	Change in C-index (95% CI)	Weight (%)
AFTCAPS	71	0.0037 (-0.0115, 0.0189)	2.02
ALLHAT	394	-0.0105 (-0.0192, -0.0018)	11.23
ARIC	593	0.0129 (0.0078, 0.0179)	16.89
BHS	69	0.0068 (-0.0102, 0.0237)	1.97
BRUN	53	0.0141 (-0.0197, 0.0479)	1.51
CHARL	52	0.0149 (-0.0020, 0.0319)	1.48
COPEN	330	0.0024 (-0.0019, 0.0067)	9.40
CUORE	143	0.0035 (-0.0030, 0.0100)	4.07
IKNS	139	-0.0061 (-0.0206, 0.0084)	3.96
KIHD	119	0.0013 (-0.0104, 0.0130)	3.39
LEADER	80	-0.0060 (-0.0233, 0.0113)	2.28
MESA	138	-0.0059 (-0.0164, 0.0046)	3.93
MGERAUG3	67	-0.0100 (-0.0224, 0.0023)	1.91
OSAKA	57	-0.0130 (-0.0327, 0.0068)	1.62
PREVEND	126	-0.0042 (-0.0114, 0.0030)	3.59
REYK	235	0.0025 (-0.0009, 0.0059)	6.70
ROTT	159	-0.0065 (-0.0220, 0.0091)	4.53
SHIP	25	0.0140 (-0.0092, 0.0371)	0.71
SHS	221	0.0121 (0.0042, 0.0199)	6.30
TARFS	24	-0.0150 (-0.0341, 0.0041)	0.68
TROMSO	329	0.0122 (0.0062, 0.0182)	9.37
ULSAM	86	0.0041 (-0.0171, 0.0254)	2.45
Overall (I-squared = 66.6%, p = 0.000)		0.0028 (0.0006, 0.0050)	100.00

Model 3 compared to Model 1

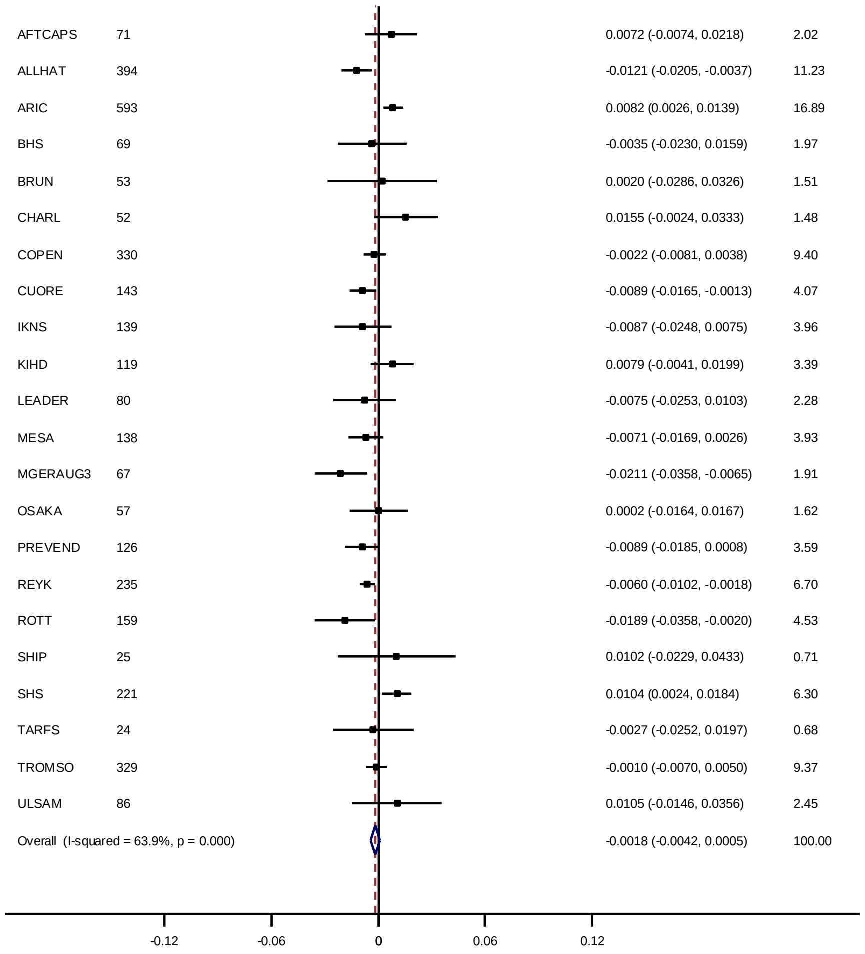

Study	No. events	Change in C-index (95% CI)	Weight (%)
AFTCAPS	71	0.0072 (-0.0074, 0.0218)	2.02
ALLHAT	394	-0.0121 (-0.0205, -0.0037)	11.23
ARIC	593	0.0082 (0.0026, 0.0139)	16.89
BHS	69	-0.0035 (-0.0230, 0.0159)	1.97
BRUN	53	0.0020 (-0.0286, 0.0326)	1.51
CHARL	52	0.0155 (-0.0024, 0.0333)	1.48
COPEN	330	-0.0022 (-0.0081, 0.0038)	9.40
CUORE	143	-0.0089 (-0.0165, -0.0013)	4.07
IKNS	139	-0.0087 (-0.0248, 0.0075)	3.96
KIHD	119	0.0079 (-0.0041, 0.0199)	3.39
LEADER	80	-0.0075 (-0.0253, 0.0103)	2.28
MESA	138	-0.0071 (-0.0169, 0.0026)	3.93
MGERAUG3	67	-0.0211 (-0.0358, -0.0065)	1.91
OSAKA	57	0.0002 (-0.0164, 0.0167)	1.62
PREVEND	126	-0.0089 (-0.0185, 0.0008)	3.59
REYK	235	-0.0060 (-0.0102, -0.0018)	6.70
ROTT	159	-0.0189 (-0.0358, -0.0020)	4.53
SHIP	25	0.0102 (-0.0229, 0.0433)	0.71
SHS	221	0.0104 (0.0024, 0.0184)	6.30
TARFS	24	-0.0027 (-0.0252, 0.0197)	0.68
TROMSO	329	-0.0010 (-0.0070, 0.0050)	9.37
ULSAM	86	0.0105 (-0.0146, 0.0356)	2.45
Overall (I-squared = 63.9%, p = 0.000)		-0.0018 (-0.0042, 0.0005)	100.00

Model 1 used baseline measures of systolic blood pressure, total cholesterol and HDL cholesterol. Model 2 used cumulative means of systolic blood pressure, total cholesterol and HDL cholesterol. Model 3 used individual-level random intercepts and slopes for systolic blood pressure, total cholesterol and HDL cholesterol. No difference in C-index between Model 2 and Model 1, and Model 3 and Model 1, were calculated for the studies “DRECE”, “GOH”, “ISRAEL”, “PROCAM”, “QUEBEC”, and “WHITE2” (all participants in the validation sample were censored in one of the age groups) and as such, these studies were excluded from the meta-analysis of change in C-index. The study “DESIR” was not included in the analysis as no participants were aged 70 years or older.

Web Figure 9B. Changes in cardiovascular disease risk discrimination in each study in participants aged ≥70 years

Model 2 compared to Model 1

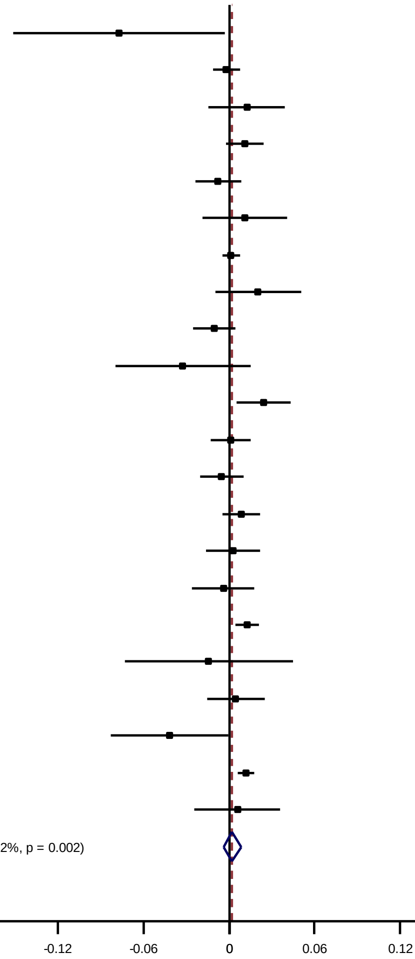

Study	No. events	Change in C-index (95% CI)	Weight (%)
AFTCAPS	71	-0.0775 (-0.1517, -0.0033)	2.02
ALLHAT	394	-0.0019 (-0.0114, 0.0077)	11.23
ARIC	593	0.0126 (-0.0142, 0.0394)	16.89
BHS	69	0.0109 (-0.0022, 0.0240)	1.97
BRUN	53	-0.0077 (-0.0240, 0.0086)	1.51
CHARL	52	0.0109 (-0.0190, 0.0409)	1.48
COPEN	330	0.0015 (-0.0048, 0.0077)	9.40
CUORE	143	0.0206 (-0.0095, 0.0506)	4.07
IKNS	139	-0.0102 (-0.0251, 0.0047)	3.96
KIHD	119	-0.0324 (-0.0796, 0.0149)	3.39
LEADER	80	0.0243 (0.0050, 0.0437)	2.28
MESA	138	0.0012 (-0.0128, 0.0151)	3.93
MGERAUG3	67	-0.0053 (-0.0204, 0.0098)	1.91
OSAKA	57	0.0086 (-0.0050, 0.0222)	1.62
PREVEND	126	0.0030 (-0.0159, 0.0218)	3.59
REYK	235	-0.0042 (-0.0264, 0.0180)	6.70
ROTT	159	0.0127 (0.0041, 0.0214)	4.53
SHIP	25	-0.0143 (-0.0733, 0.0447)	0.71
SHS	221	0.0045 (-0.0157, 0.0248)	6.30
TARFS	24	-0.0420 (-0.0833, -0.0007)	0.68
TROMSO	329	0.0119 (0.0062, 0.0176)	9.37
ULSAM	86	0.0058 (-0.0241, 0.0357)	2.45
Overall (I-squared = 52.2%, p = 0.002)		0.0023 (-0.0036, 0.0082)	100.00

Model 3 compared to Model 1

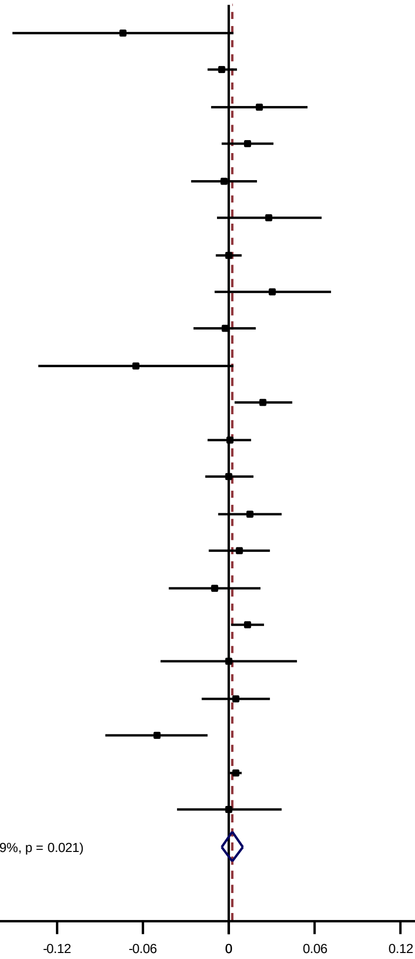

Study	No. events	Change in C-index (95% CI)	Weight (%)
AFTCAPS	71	-0.0734 (-0.1506, 0.0038)	2.02
ALLHAT	394	-0.0043 (-0.0144, 0.0057)	11.23
ARIC	593	0.0218 (-0.0119, 0.0555)	16.89
BHS	69	0.0133 (-0.0049, 0.0316)	1.97
BRUN	53	-0.0031 (-0.0263, 0.0201)	1.51
CHARL	52	0.0285 (-0.0079, 0.0649)	1.48
COPEN	330	-0.0000 (-0.0089, 0.0089)	9.40
CUORE	143	0.0308 (-0.0096, 0.0713)	4.07
IKNS	139	-0.0026 (-0.0240, 0.0188)	3.96
KIHD	119	-0.0647 (-0.1331, 0.0036)	3.39
LEADER	80	0.0245 (0.0046, 0.0443)	2.28
MESA	138	0.0007 (-0.0144, 0.0158)	3.93
MGERAUG3	67	0.0006 (-0.0164, 0.0175)	1.91
OSAKA	57	0.0150 (-0.0074, 0.0375)	1.62
PREVEND	126	0.0079 (-0.0134, 0.0292)	3.59
REYK	235	-0.0097 (-0.0415, 0.0221)	6.70
ROTT	159	0.0131 (0.0016, 0.0245)	4.53
SHIP	25	0.0000 (-0.0477, 0.0477)	0.71
SHS	221	0.0054 (-0.0183, 0.0290)	6.30
TARFS	24	-0.0499 (-0.0856, -0.0142)	0.68
TROMSO	329	0.0053 (0.0012, 0.0094)	9.37
ULSAM	86	0.0006 (-0.0356, 0.0368)	2.45
Overall (I-squared = 41.9%, p = 0.021)		0.0029 (-0.0046, 0.0103)	100.00

Model 1 used baseline measures of systolic blood pressure, total cholesterol and HDL cholesterol. Model 2 used cumulative means of systolic blood pressure, total cholesterol and HDL cholesterol. Model 3 used individual-level random intercepts and slopes for systolic blood pressure, total cholesterol and HDL cholesterol. No difference in C-index between Model 2 and Model 1, and Model 3 and Model 1, were calculated for the studies "DRECE", "GOH", "ISRAEL", "PROCAM" and "WHITE2" (all participants in the validation sample were censored in one of the age groups) and as such, these studies were excluded from the meta-analysis of change in C-index. The study "DESIR" was not included in the analysis as no participants were aged 70 years or older.

Web Figure 10. Changes in 10-year cardiovascular disease risk discrimination in each study, restricted to studies with 10 years of follow-up

Model 2 compared to Model 1

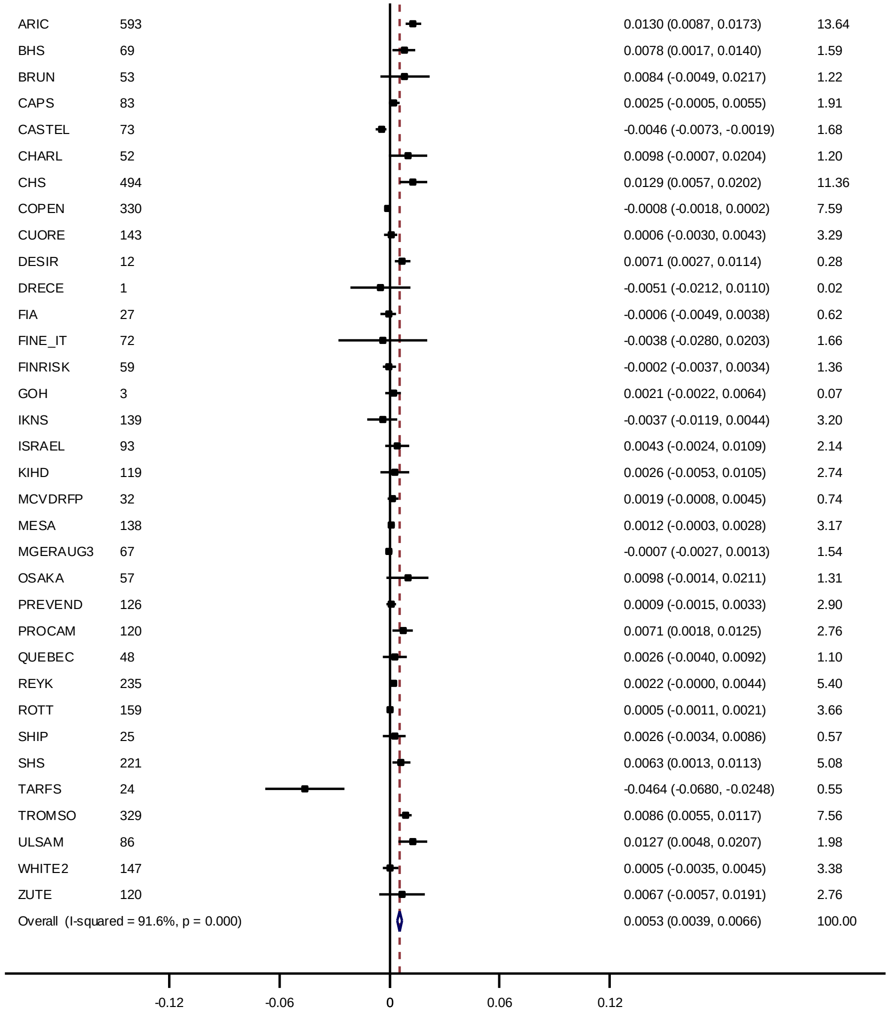

Study	No. events	Change in C-index (95% CI)	Weight (%)
ARIC	593	0.0130 (0.0087, 0.0173)	13.64
BHS	69	0.0078 (0.0017, 0.0140)	1.59
BRUN	53	0.0084 (-0.0049, 0.0217)	1.22
CAPS	83	0.0025 (-0.0005, 0.0055)	1.91
CASTEL	73	-0.0046 (-0.0073, -0.0019)	1.68
CHARL	52	0.0098 (-0.0007, 0.0204)	1.20
CHS	494	0.0129 (0.0057, 0.0202)	11.36
COPEN	330	-0.0008 (-0.0018, 0.0002)	7.59
CUORE	143	0.0006 (-0.0030, 0.0043)	3.29
DESIR	12	0.0071 (0.0027, 0.0114)	0.28
DRECE	1	-0.0051 (-0.0212, 0.0110)	0.02
FIA	27	-0.0006 (-0.0049, 0.0038)	0.62
FINE_IT	72	-0.0038 (-0.0280, 0.0203)	1.66
FINRISK	59	-0.0002 (-0.0037, 0.0034)	1.36
GOH	3	0.0021 (-0.0022, 0.0064)	0.07
IKNS	139	-0.0037 (-0.0119, 0.0044)	3.20
ISRAEL	93	0.0043 (-0.0024, 0.0109)	2.14
KIHD	119	0.0026 (-0.0053, 0.0105)	2.74
MCVDRFP	32	0.0019 (-0.0008, 0.0045)	0.74
MESA	138	0.0012 (-0.0003, 0.0028)	3.17
MGERAUG3	67	-0.0007 (-0.0027, 0.0013)	1.54
OSAKA	57	0.0098 (-0.0014, 0.0211)	1.31
PREVEND	126	0.0009 (-0.0015, 0.0033)	2.90
PROCAM	120	0.0071 (0.0018, 0.0125)	2.76
QUEBEC	48	0.0026 (-0.0040, 0.0092)	1.10
REYK	235	0.0022 (-0.0000, 0.0044)	5.40
ROTT	159	0.0005 (-0.0011, 0.0021)	3.66
SHIP	25	0.0026 (-0.0034, 0.0086)	0.57
SHS	221	0.0063 (0.0013, 0.0113)	5.08
TARFS	24	-0.0464 (-0.0680, -0.0248)	0.55
TROMSO	329	0.0086 (0.0055, 0.0117)	7.56
ULSAM	86	0.0127 (0.0048, 0.0207)	1.98
WHITE2	147	0.0005 (-0.0035, 0.0045)	3.38
ZUTE	120	0.0067 (-0.0057, 0.0191)	2.76
Overall (I-squared = 91.6%, p = 0.000)		0.0053 (0.0039, 0.0066)	100.00

Model 3 compared to Model 1

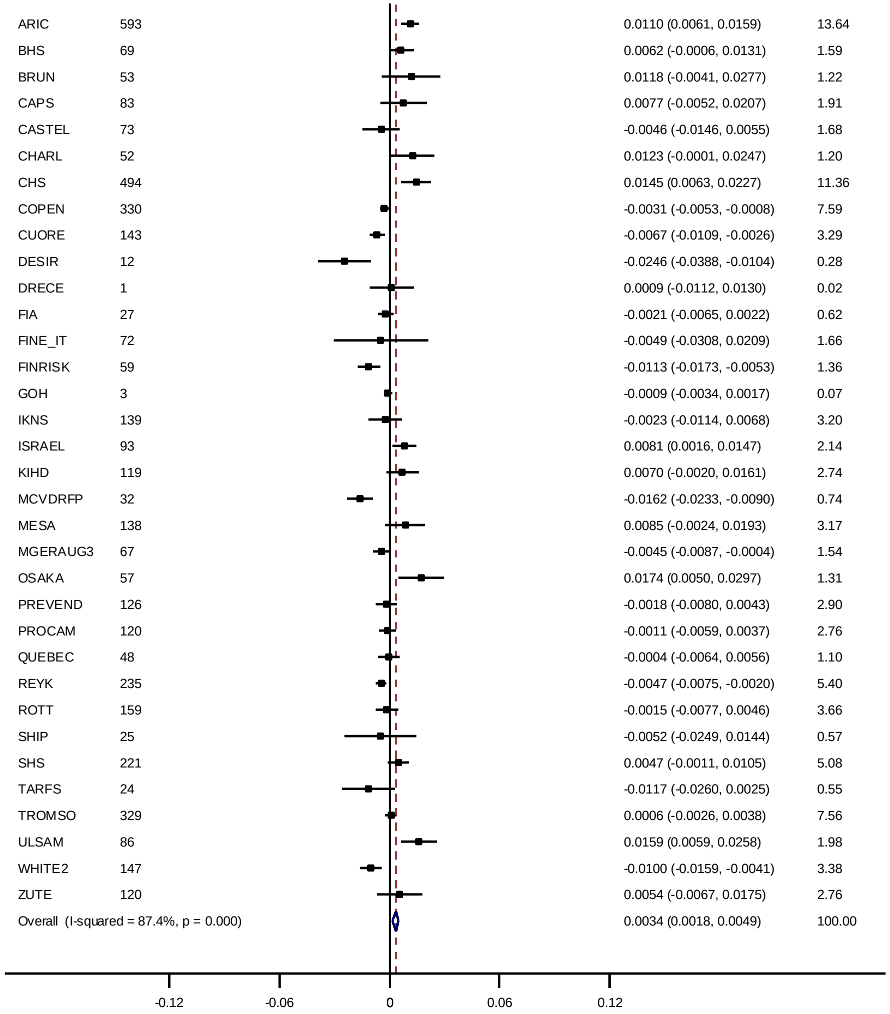

Study	No. events	Change in C-index (95% CI)	Weight (%)
ARIC	593	0.0110 (0.0061, 0.0159)	13.64
BHS	69	0.0062 (-0.0006, 0.0131)	1.59
BRUN	53	0.0118 (-0.0041, 0.0277)	1.22
CAPS	83	0.0077 (-0.0052, 0.0207)	1.91
CASTEL	73	-0.0046 (-0.0146, 0.0055)	1.68
CHARL	52	0.0123 (-0.0001, 0.0247)	1.20
CHS	494	0.0145 (0.0063, 0.0227)	11.36
COPEN	330	-0.0031 (-0.0053, -0.0008)	7.59
CUORE	143	-0.0067 (-0.0109, -0.0026)	3.29
DESIR	12	-0.0246 (-0.0388, -0.0104)	0.28
DRECE	1	0.0009 (-0.0112, 0.0130)	0.02
FIA	27	-0.0021 (-0.0065, 0.0022)	0.62
FINE_IT	72	-0.0049 (-0.0308, 0.0209)	1.66
FINRISK	59	-0.0113 (-0.0173, -0.0053)	1.36
GOH	3	-0.0009 (-0.0034, 0.0017)	0.07
IKNS	139	-0.0023 (-0.0114, 0.0068)	3.20
ISRAEL	93	0.0081 (0.0016, 0.0147)	2.14
KIHD	119	0.0070 (-0.0020, 0.0161)	2.74
MCVDRFP	32	-0.0162 (-0.0233, -0.0090)	0.74
MESA	138	0.0085 (-0.0024, 0.0193)	3.17
MGERAUG3	67	-0.0045 (-0.0087, -0.0004)	1.54
OSAKA	57	0.0174 (0.0050, 0.0297)	1.31
PREVEND	126	-0.0018 (-0.0080, 0.0043)	2.90
PROCAM	120	-0.0011 (-0.0059, 0.0037)	2.76
QUEBEC	48	-0.0004 (-0.0064, 0.0056)	1.10
REYK	235	-0.0047 (-0.0075, -0.0020)	5.40
ROTT	159	-0.0015 (-0.0077, 0.0046)	3.66
SHIP	25	-0.0052 (-0.0249, 0.0144)	0.57
SHS	221	0.0047 (-0.0011, 0.0105)	5.08
TARFS	24	-0.0117 (-0.0260, 0.0025)	0.55
TROMSO	329	0.0006 (-0.0026, 0.0038)	7.56
ULSAM	86	0.0159 (0.0059, 0.0258)	1.98
WHITE2	147	-0.0100 (-0.0159, -0.0041)	3.38
ZUTE	120	0.0054 (-0.0067, 0.0175)	2.76
Overall (I-squared = 87.4%, p = 0.000)		0.0034 (0.0018, 0.0049)	100.00

Model 1 used baseline measures of systolic blood pressure, total cholesterol and HDL cholesterol. Model 2 used cumulative means of systolic blood pressure, total cholesterol and HDL cholesterol. Model 3 used individual-level random intercepts and slopes for systolic blood pressure, total cholesterol and HDL cholesterol.
